# Supramolecular Carbohydrate Assemblies with Tunable Glycan Surfaces

**DOI:** 10.1002/anie.202515926

**Published:** 2025-11-23

**Authors:** Nives Hribernik, Marlene C. S. Dal Colle, Junki Fujihara, Jacobus P. van Trijp, Kai Ludwig, Yu Ogawa, Katharina Ribbeck, Martina Delbianco

**Affiliations:** ^1^ Department of Biomolecular Systems Max Planck Institute of Colloids and Interfaces Am Mühlenberg 1 14476 Potsdam Germany; ^2^ Department of Chemistry and Biochemistry Freie Universität Berlin Arnimallee 22 14195 Berlin Germany; ^3^ Forschungszentrum für Elektronenmikroskopie and Core Facility BioSupraMol Institut für Chemie und Biochemie Freie Universität Berlin Fabeckstr. 36a 14195 Berlin Germany; ^4^ CNRS CERMAV Univ. Grenoble Alpes Grenoble 38000 France; ^5^ Department of Sustainable and Bio‐inspired Materials Max Planck Institute of Colloids and Interfaces Am Mühlenberg 1 14476 Potsdam Germany; ^6^ Department of Biological Engineering Massachusetts Institute of Technology Cambridge MA 02139 USA

**Keywords:** *Candida albicans*, Glycans, Hydrogels, Nanomaterials

## Abstract

The self‐assembly of molecular building blocks into ordered supramolecular structures enables the creation of nanomaterials that can display ligands on their surfaces with molecular precision. However, many of these supramolecular scaffolds face challenges in incorporating bulky or hydrophilic ligands, such as carbohydrates. This issue often requires the co‐assembly of ligand‐containing blocks with non‐functionalized ones, diluting ligand presentation and compromising their precise spatial arrangement. Herein, we present carbohydrate oligomers that assemble into supramolecular nanomaterials featuring a molecularly controlled, dense presentation of carbohydrate ligands on their surfaces. This modular system accommodates a variety of carbohydrate ligands while maintaining consistent bulk material properties. Using this approach, we have engineered a series of supramolecular hydrogels, whose nanostructure displays specific carbohydrate residues with high density that act as biological cues to influence the morphology of *Candida albicans*.

## Graphical abstract

Synthetic cellulose‐based oligomers assemble into supramolecular nanomaterials with a molecularly controlled, dense presentation of carbohydrates on their surfaces, serving as biological cues.

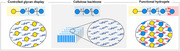

## Introduction

Engineering the surface of nanomaterials with atomic precision could give rise to unique physical and chemical properties.^[^ ^1^, ^2^ ^]^ Materials with a patterned presentation of ligands on their surface have been exploited to mimic the multivalent interactions that regulate biological processes, like cell adhesion and signaling.^[^ ^3^, ^4^, ^5^ ^]^ In this context, nanoparticles (NPs),^[^ ^6^, ^7^, ^8^, ^9^, ^10^ ^]^ polymers,^[^ ^11^, ^12^ ^]^ or metal complexes^[^ ^13^, ^14^, ^15^ ^]^ are commonly employed as scaffolds for ligand presentation. However, these scaffolds can only guarantee a statistical ligand distribution on their surface (NPs and polymers)^[^ ^16^, ^17^, ^18^ ^]^ or can accommodate a relatively small number of ligands (metal complexes).^[^ ^19^, ^20^ ^]^

An alternative approach to control ligand spatial distribution on nanomaterials is through self‐assembly, following the strategic design of molecular building blocks that arrange into ordered structures.^[^ ^21^ ^]^ Self‐assembling peptides,^[^ ^22^, ^23^, ^24^, ^25^ ^]^ small molecule amphiphiles,^[^ ^26^, ^27^ ^]^ and DNA origami^[^ ^28^, ^29^ ^]^ are representative scaffolds used to obtain a controlled presentation of ligands. However, in this case, the ligand modifications tend to affect significantly the bulk properties of the materials.^[^ ^30^, ^31^, ^32^ ^]^ This challenge is particularly pronounced when presenting large or highly water‐soluble ligands, such as carbohydrates.^[^ ^33^, ^34^ ^]^ To overcome this issue, ligand‐presenting monomers are frequently co‐assembled with non‐functionalized ones, leading to a dilution of ligand density, thus losing information on the exact spatial presentation of ligands on the nanomaterial surface.^[^ ^35^, ^36^, ^37^, ^38^ ^]^ Precise presentation of carbohydrate ligands, including density, spacing, and orientation, is crucial for their recognition by the carbohydrate‐binding proteins.^[^ ^39^, ^40^, ^41^ ^]^ Platforms that can enforce periodic, oriented glycan presentation, would therefore yield enhanced and more predictable biological potency.

Here, we show that glycan oligomers can self‐assemble into crystalline nanomaterials with controlled surfaces, providing a suitable platform for tailored ligand presentation. Cellulose oligomers serve as backbone to guide the assembly process,^[^ ^42^ ^]^ generating nanocrystals that can sustain a dense presentation of glycan epitopes on their surfaces. This supramolecular approach can be further extended to create hydrogel matrices, whose nanostructure displays specific carbohydrate residues with high density. The surface functionalization with various monosaccharide epitopes hardly affect the bulk properties of the material, but offers important biological cues that dramatically influence cell differentiation, as exemplified in a filamentation assay with *C. albicans*, a fungus whose morphology and virulence factors are known to be regulated by glycan epitopes present in its environment.^[^ ^43^ ^]^

## Results and Discussion

### Cellulose Nanocrystals with Patterned Surfaces

Cellulose oligomers have been previously exploited for the generation of glyco‐amphiphiles that assembled into nanomaterials.^[^ ^44^, ^45^, ^46^ ^]^ These structures are normally obtained from the degradation of natural polymers or by enzymatic synthesis and therefore exist as ill‐defined mixtures.^[^ ^44^, ^47^ ^]^ In contrast, we focused on fully synthetic oligomers produced by automated glycan assembly (AGA) (experimental details in Supporting Information).^[^ ^48^ ^]^ This strategy allowed us to control the sequence, the length, and the modification of our oligomers, including the incorporation of glycan epitopes.

**Figure 1:**
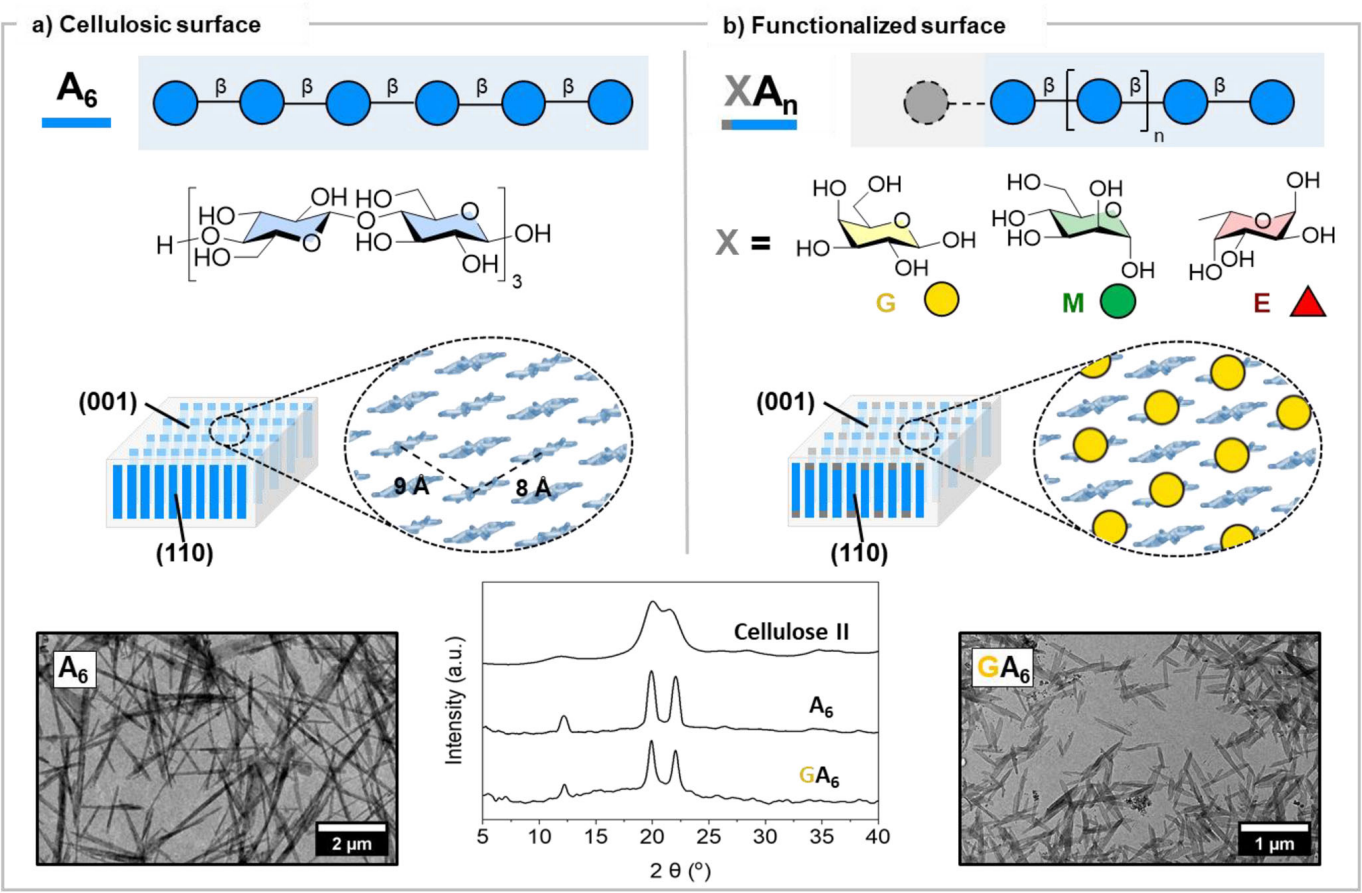
Cellulose‐based nanomaterials with patterned surfaces. a): Structure and assembly of cellulose hexamer **A_6_**. b): SNFG^[^ ^51^ ^]^ structure representation and assembly of functionalized cellulose oligomers **XA_n_**, exemplified for **GA_6_**. Comparison of XRD and TEM of crystallites obtained from the assembly of **A_6_** and **GA_6_** confirming the cellulose II type of structure and morphological similarities between the two assemblies.

Synthetic cellulose hexasaccharides (**A_6_**) spontaneously self‐assemble into nanocrystals in aqueous solution.^[^ ^42^, ^49^ ^]^ Within the crystals, the oligoglucoside chains stack in an antiparallel fashion, forming a cellulose II crystal structure (Figure 1a). In this arrangement, the (0 0 1) crystal surfaces on the top and bottom side of the lamellar plane present a regular display of alternating reducing and non‐reducing end glucose (D‐Glc) units (Figure 1a, the distances were obtained from the crystal structure of cellulose II).^[^ ^50^ ^]^ Thus, the incorporation of a different monosaccharide at one terminus of the cellulose oligomer should result in nanomaterials with a controlled presentation of non‐glucosidic ligands on their surfaces.

To explore the tolerance of the assembly process for cellulose oligomers incorporating non‐glucose moieties in their structure, we synthesized **GA_6_** (Figure 1b), a cellulose‐based heptamer carrying a β‐linked D‐galactose (D‐Gal) moiety at the non‐reducing end. Transmission electron microscopy (TEM) and atomic force microscopy (AFM) analyses confirmed that **GA_6_** assembled into nanocrystals with length of around 100 nm that form bundles upon drying (Figures 1 and S5), as observed for the non‐modified cellulose oligomer **A_6_**.^[^ ^42^ ^]^ An annealing step further promoted the assembly into longer rod‐like nanocrystals (Figures 1, S5 and S6).^[^ ^49^ ^]^ The X‐ray diffraction (XRD) analysis revealed aggregation into the cellulose II structure (Figure 1), confirming the antiparallel arrangement of the **GA_6_** oligomers. Thus, we concluded that **GA_6_** assembles into nanocrystals with a crystallite surface presenting an alternating pattern of reducing D‐Glc and non‐reducing D‐Gal units (Figure 1b).

We then tested the incorporation of other monosaccharides, such as the α‐linked D‐mannose (**M**) and the α‐linked L‐fucose (**E**), into the self‐assembling cellulose backbone (**MA_6_** and **EA_6_**, respectively). The **MA_6_** heptamer formed small nanocrystallites that could be promoted into longer rod‐like structures following an annealing step (Figures S5 and S6). However, the increased solubility of **EA_6_** disrupted the assembly of this oligomer (Figure S5). Extending the cellulose stacking core with an additional D‐Glc unit (**EA_7_**) successfully produced rod‐like structures (Figures S5 and S6). XRD analysis confirmed that the cellulose backbone drove the assembly into the cellulose II‐type crystal structure (Figure S4) even in the presence of more pronounced structural differences in the exposed monosaccharides (i.e., α‐glycosidic linkage and axial hydroxyl groups). Overall, these results confirmed that cellulose‐based oligomers can be functionalized with other monosaccharide epitopes, creating supramolecular assemblies with a precise control over glycan display on their surface.

### From Nanocrystals to Supramolecular Hydrogels

To further expand our nanocrystal portfolio, we tested the incorporation of a non‐natural 3,6‐methylated glucose unit (**C**). The XRD profile of heptamer **CA_6_** still showed the cellulose II structure and TEM confirmed the formation of nanocrystals (Figure S7). However, upon annealing, we did not observe a solid precipitate; instead, the nanocrystals were colloidally stable generating a gel‐like system. We hypothesized that the densely methylated surfaces could promote hydrophobic transient interactions between nanocrystals, generating extended networks and resulting in the formation of a gel‐like system.

**Figure 2:**
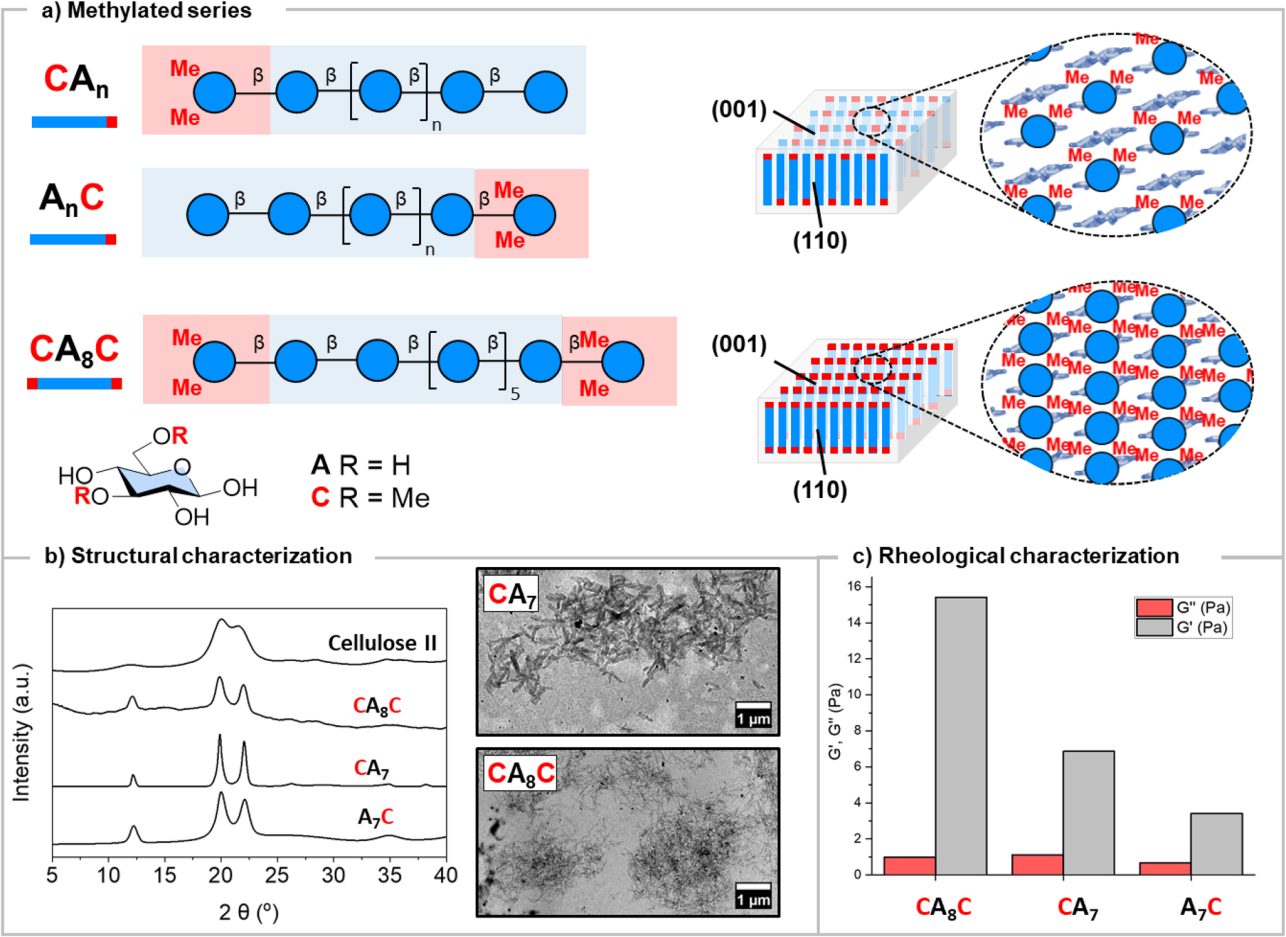
a): Structure and assembly of methylated oligomers **CA_n_**, **A_n_C** and **CA_8_C**. b): XRD analysis confirmed the cellulose II structure for all methylated analogues and TEM imaging of the assemblies of **CA_7_** and **CA_8_C** showed a network of nanocrystals. c): Comparison of G′ and G″ moduli of 2.0% (w/w) hydrogels from **CA_8_C**, **CA_7_**, and **A_7_C** in a frequency sweep experiment (25 °C).

To better understand this supramolecular analogue of methyl cellulose, a methylated derivative of cellulose commonly employed as gelator,^[^ ^52^ ^]^ we designed a collection of oligomers varying the backbone length (**A_n_**) and the position of the methylated unit (**C**). The methylated unit (**C**) was placed at the non‐reducing (**CA_7_**, **CA_6_**) or reducing (**A_7_C**, **A_6_C**) end or at both termini (**CA_8_C**), the latter producing nanocrystals with fully functionalized surfaces (Figure 2a).

The spontaneous formation of cellulose II type crystallites was confirmed for all oligosaccharides by XRD analysis and TEM imaging (Figures S8 and S9). Upon annealing, the 2.0% (w/w) suspensions transformed into opaque gel‐like samples. (Cryo‐)TEM and AFM observations indicated the formation of networks of fibrillar crystallites (Figures S8, S16, and S17).

Rheological analysis indicated a typical gel behavior for the 2.0% (w/w) samples of **CA_8_C**, **CA_7_** and **A_7_C**, with the storage moduli (G′) greater than the loss moduli (G″) in a frequency sweep experiment at 25 °C (Figures 2 and S10). The 2.0% (w/w) hydrogel of **CA_8_C** exhibited the highest storage (G′) modulus of approx. 15 Pa at 25 °C and a fast, self‐healing behavior in a recovery test where we alternated between intervals of low and high strain (Figure S11). In contrast, the 2.0% (w/w) **CA_6_** and **A_6_C** samples only showed weak gel‐like properties in the frequency sweep at the same temperature (Figure S10).

This screening indicated that a single **C** unit (either at the reducing or non‐reducing end), thus an alternating display of the methylated glucosides, is sufficient to form a hydrogel. In addition, the length of the cellulose backbone determined the hydrogel mechanical properties, with longer backbones leading to mechanically stronger hydrogels. The stiffness of the hydrogel could be further tuned with concentration (Figure S12) and temperature (Figure S13), showing similarities to the thermoresponsive behavior of methylcellulose.^[^ ^53^ ^]^ At a temperature around 5 °C the hydrogel turned into a liquid, and the gel‐like properties were restored above 10 °C, with a further increase of stiffness with increasing temperature (Figures S14 and S15).

### Densely Glycosylated Supramolecular Hydrogels

**Figure 3:**
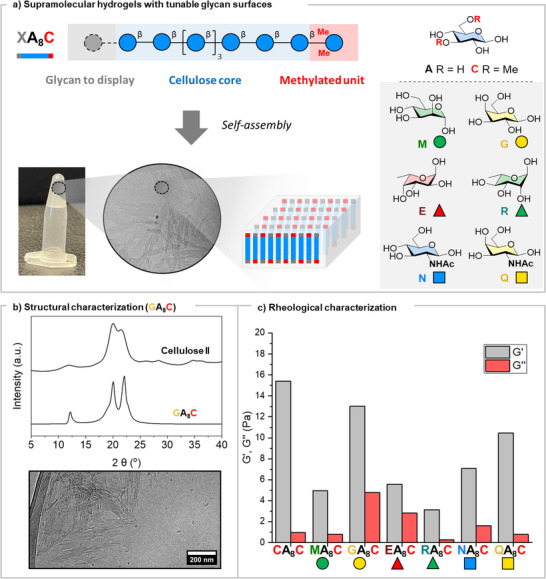
Supramolecular cellulose‐based hydrogels with dense presentation of glycan epitopes on their surface. a): Schematic representation of **XA_8_C** oligomers and their assemblies. b): Structural characterization of **GA_8_C** by XRD and cryo‐TEM. c): Comparison of storage (G′) and loss (G″) moduli of 2.0% (w/w) hydrogels obtained from different **XA_8_C** oligomers calculated as an average of the linear viscoelastic region in a frequency sweep experiment (25 °C).

The knowledge obtained from these two series of compounds suggested that careful engineering of the crystallite surface could result in supramolecular hydrogels with a dense display of glycan epitopes. To this end, we designed an octasaccharide cellulose core (**A_8_**) substituted with a 3,6‐dimethyl glucose unit (**C**) at the reducing end and a glycan epitope (**X**) at the non‐reducing end. In this design, the central cellulose core (**A_8_**) was planned to drive the assembly into crystallites displaying on their surfaces a regular pattern of unit **C**, to trigger hydrogel formation, and unit **X**, as functional glycan epitope (Figure 3a). Because the (0 0 1) crystal surface alternates reducing and non‐reducing termini, this pattern yields a glycan density of ∼1.5 glycan nm^−^^2^, with a regular inter‐glycan distance of ∼0.90 nm.^[^ ^54^ ^]^

As a proof of concept, we synthesized and analyzed the self‐assembly of a D‐Gal functionalized oligomer **GA_8_C**. XRD analysis confirmed the cellulose II‐type structure (Figure 3b), indicating the robustness of the cellulose core in accommodating the double modification while preserving the molecular organization and self‐assembly behavior of the unsubstituted analogue. Upon annealing, the 2.0% (w/w) suspension of **GA_8_C** in water transitioned into a soft hydrogel with stiffness (G′, storage modulus) of around 12 Pa (Figures 3c and S21). A network of nanocrystals with a thickness of approx. 6 nm was observed with (cryo‐)TEM and AFM imaging (Figures 3b and S20).

The system proved modular, allowing us to incorporate other monosaccharide units in place of the D‐Gal units. We tested D‐Man (**M**), L‐Rha (**R**), L‐Fuc (**E**), D‐GlcNAc (**N**), and D‐GalNAc (**Q**) (Figure 3a). Regardless of the epitope displayed, the morphology and bulk mechanical properties of the hydrogels remained mostly unaffected. Upon annealing, all oligomers formed a network of nanocrystallites (Figure S19) with soft gel‐like properties (Figures 3c and S21).

### The Displayed Glycans as Biological Cue

The toolbox of synthetic hydrogels displaying different glycan epitopes was tested in a biological context, to study whether the different displayed glycans can impact cell behavior. Indeed, glycans exposed on biological matrices are known biological cues that can influence cell growth and morphology.^[^ ^55^, ^56^, ^57^ ^]^

As a representative example, we looked into the morphology of *C. albicans*, an opportunistic fungal pathogen which is part of the human microbiota. *C. albicans* is embedded in mucus, a viscoelastic matrix formed by densely glycosylated glycoproteins (i.e., mucins) covering all non‐keratinized surfaces of the human body.^[^ ^43^ ^]^ *C. albicans* cells respond to environmental biochemical signals arising from the glycans in the environment,^[^ ^58^, ^59^ ^]^ including those displayed on mucins,^[^ ^43^ ^]^ evolving into distinct morphological states: from a unicellular round‐shaped budding yeast form, to a variety of growth forms that can be referred to as pseudo‐hyphae, until reaching the elongated true hyphae state with parallel‐sided walls.^[^ ^60^ ^]^ The filamentation of *C. albicans* from yeast form to hyphae is associated with its infectious state.^[^ ^43^ ^]^

Various backbones have previously been used to present glycan ligands in the context of mucin mimics, yet they either lack the fixed spacing and orientation,^[^ ^61^, ^62^, ^63^, ^64^ ^]^ or utilize a scaffold that remains conformationally dynamic in solution.^[^ ^65^ ^]^ In contrast, our carbohydrate‐based system displays glycans on crystalline cellulose‐II nanocrystal faces, enforcing a periodic, lattice‐defined orientation at high surface density while keeping bulk mechanics essentially constant across epitopes. This allowed us to vary epitope identity without diluting density or altering gel properties.

**Figure 4:**
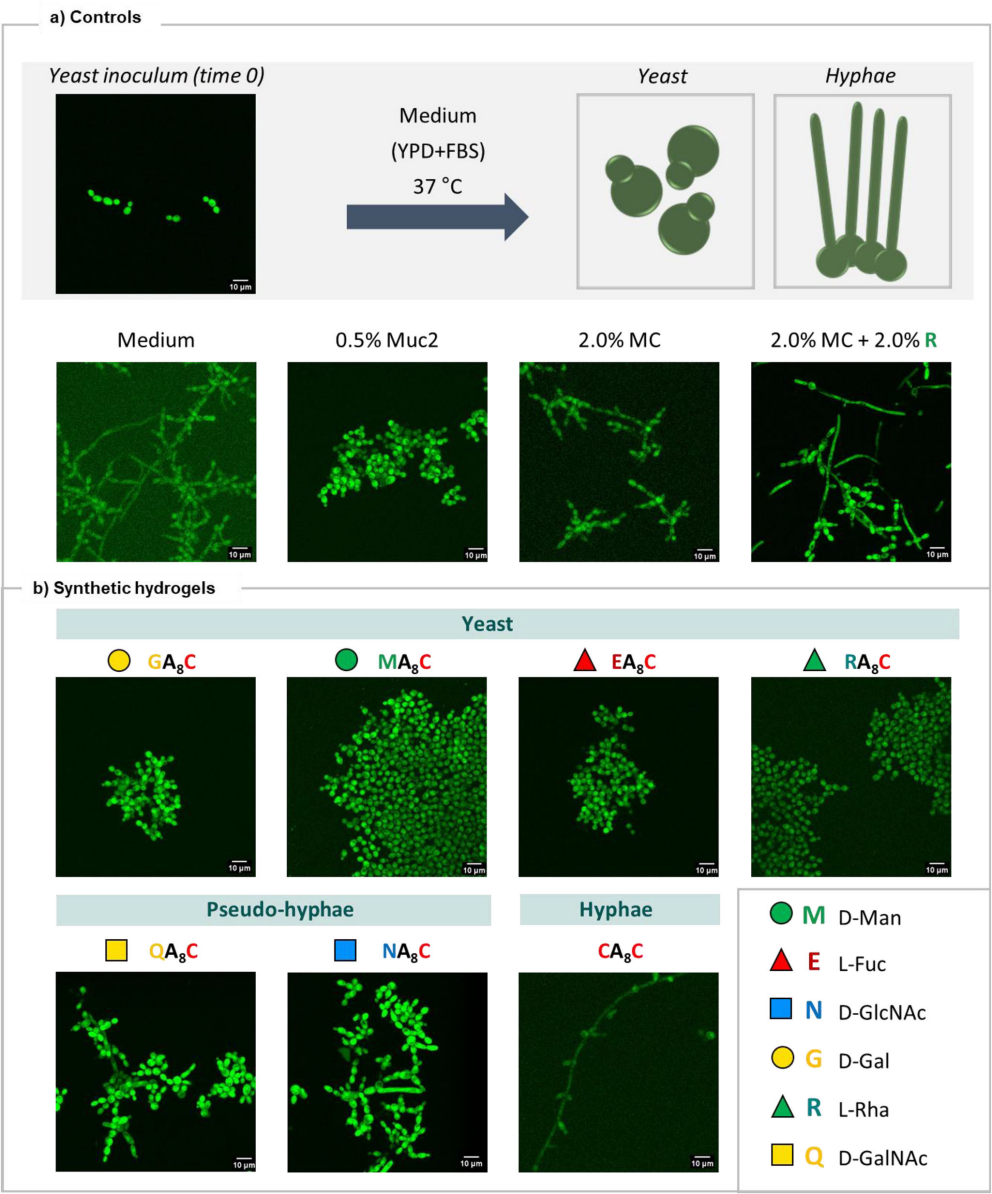
Fluorescence microscopy images of the filamentation of *C. albicans* cells after 8 h in the presence of a): controls (YPD medium with 10% FBS, 0.5% (w/w) Muc2, 2.0% (w/w) methylcellulose MC, 2.0% (w/w) MC + 2.0% (w/w) L‐Rha) and b): 2.0% (w/w) synthetic hydrogels **GA_8_C**, **MA_8_C**, **EA_8_C**, **RA_8_C**, **QA_8_C**, **NA_8_C**, **CA_8_C**. Scale bar 10 µm. Further experimental details are reported in Supporting Information section 3.

To study whether the morphology of *C. albican*s could be influenced by the glycans presented on our hydrogels, we performed a filamentation assay.^[^ ^43^ ^]^ In this assay, the *C. albicans* yeast cells were grown under conditions that promote filamentation, utilizing YPD + 10% FBS medium at 37 °C, and observed with a confocal microscope (details in Supporting Information section 3). Under these conditions, the yeast‐to‐hyphae transition occurred within the first 3 h. The hyphae form dominated also after 8, 24 and 48 h (Figures 4 and S22–S25). When the same assay was performed in the presence of 0.5% (w/w) Muc2, a densely glycosylated protein,^[^ ^66^ ^]^ *C. albicans* filamentation was prevented and the yeast form was observed at all time points (Figures 4 and S22–S25).^[^ ^43^ ^]^

Next, the filamentation assay was performed in the presence of our synthetic hydrogels, showing dramatically different results depending on the type of monosaccharide displayed. In the presence of **GA_8_C**, **MA_8_C**, **EA_8_C**, and **RA_8_C** hydrogels, mainly the yeast form was observed over short (3 and 8 h) and long (24 and 48 h) time periods (Figures 4 and S22–S25). The presence of **QA_8_C** and **NA_8_C** hydrogels stopped filamentation at the pseudo‐hyphae state, never fully transitioning into hyphae even after 48 h (Figures 4 and S22–S25). In contrast, the presence of the **CA_8_C** hydrogel, lacking functional monosaccharide epitopes, did not significantly inhibit the filamentation of *C. albicans*, and hyphae were the predominant forms observed at all time points (Figures 4 and S22–S25). The viability assay showed that the differences in morphology of *C. albicans* cells were not due to cytotoxicity (Figure S26).

Additional control studies were performed to confirm the prominent role of the displayed glycan epitopes on the morphological changes of *C. albicans* cells. Filamentation assays in the presence of 0.5% (w/w) and 2.0% (w/w) methylcellulose (MC) resulted in hyphae formation at all time points (Figures 4a and S22–S25), excluding the major contribution of the increased stiffness of the cell environment. Similarly, the hyphae form was predominant when the assay was repeated in the presence of 2.0% (w/w) L‐Rha monosaccharide dissolved in the medium (Figures 4a and S22–S25) as well as with the addition of 2.0% (w/w) L‐Rha and 2.0% (w/w) MC (Figures 4a and S22–S25). Taken together, these results demonstrated the importance of multivalent epitope presentation on the self‐assembling scaffold to direct *C. albicans* differentiation.

## Conclusion

In this study, we demonstrated that cellulose oligomers can serve as a versatile scaffold for constructing supramolecular nanomaterials with a dense arrangement of glycan epitopes on their surface. The cellulose backbone ensures precise molecular organization, allowing for complete control over the nanomaterial surface. This system is adaptable and robust to accommodate various monosaccharide units which are displayed on the nanocrystallite surface in a multivalent fashion. Furthermore, we extended this strategy to develop supramolecular hydrogels that integrate dynamic viscoelastic properties with a dense multivalent glycan presentation, mimicking key features of natural extracellular matrices like mucus.^[^ ^67^, ^68^ ^]^ The ability of this system to influence cellular behavior was evidenced by its impact on the morphology of *C. albicans*, triggering or preventing filamentation depending on the displayed glycan. A transcriptomic analysis of *C. albicans* exposed to our synthetic hydrogels is planned for future work and could guide the rational design of anti‐virulence hydrogels against this microorganism. Further, the implementation of more complex carbohydrate epitopes is currently underway. A unique opportunity of our supramolecular system is the possibility of hetero‐multivalent display; by co‐assembling oligomers with various terminal units, we can create materials that simultaneously present an array of epitopes, holding promise for advancements in antimicrobial treatments,^[^ ^69^, ^70^ ^]^ wound healing,^[^ ^71^, ^72^, ^73^ ^]^ and tissue engineering.^[^ ^74^, ^75^ ^]^

## Supporting Information

The authors have cited additional references within the Supporting Information.^[^ ^76^, ^77^, ^78^, ^79^, ^80^, ^81^, ^82^, ^83^, ^84^, ^85^, ^86^, ^87^, ^88^, ^89^, ^90^ ^]^

## Author Contributions

NH and MD conceived the project. NH synthesized the compounds, performed XRD analysis, rheometric experiments, AFM imaging and filamentation assays with *C. albicans*. MDC synthesized the compounds and performed TEM imaging. JF synthesized the compounds and performed AFM imaging. JT synthesized the compounds. YO and KL performed (cryo‐)TEM analysis. MD and KR supervised the project. NH and MD wrote the paper with contributions from all authors.

## Conflict of Interests

The authors declare no conflict of interest.

## Supporting information



Supporting Information

## Data Availability

All experimental details regarding building block synthesis, AGA, NMR, XRD, TEM, AFM, rheology and bioassays are reported in the Supporting Information. Raw data for NMR analysis, XRD and rheology can be downloaded from https://doi.org/10.17617/3.AINRHN, Edmond.
