# Supplementary material for: Supramolecular Carbohydrate Assemblies with Tunable Glycan Surfaces
Source: Angew Chem Int Ed Engl. 2025 Nov 23;65(3):e15926. doi: 10.1002/anie.202515926 (PMC12811653; doi:10.1002/anie.202515926)
Supplement: Supplementary file 1 — Supporting Information [file ANIE-65-e15926-s001.pdf]

Supporting information

# Supramolecular Carbohydrate Assemblies with Tunable Glycan Surfaces

Nives Hribernik, Marlene C. S. Dal Colle, Junki Fujihara, Jacobus P. van Trijp, Kai Ludwig, Yu Ogawa, Katharina Ribbeck, Martina Delbianco

# Table of Contents


1 Synthesis ..... 4
1.1 General materials and methods ..... 4
1.2 Building blocks ..... 5
1.3 Automated Glycan Assembly ..... 6
1.3.1 General materials and methods ..... 6
1.3.2 Preparation of stock solutions ..... 6
1.3.3 Modules for automated synthesis ..... 6
1.3.4 Post-AGA manipulations ..... 9
1.4 Oligosaccharide synthesis ..... 10
1.4.1 Synthesis of $GA_6$ ..... 12
1.4.2 Synthesis of $MA_6$ ..... 15
1.4.4 Synthesis of $EA_6$ ..... 18
1.4.5 Synthesis of $EA_7$ ..... 21
1.4.6 Synthesis of $CA_7$ ..... 24
1.4.8 Synthesis of $CA_6$ ..... 27
1.4.10 Synthesis of $A_7C$ ..... 30
1.4.11 Synthesis of $A_6C$ ..... 33
1.4.12 Synthesis of $MA_8C$ ..... 36
1.4.13 Synthesis of $GA_8C$ ..... 39
1.4.14 Synthesis of $NA_8C$ ..... 42
1.4.15 Synthesis of $QA_8C$ ..... 45
1.4.16 Synthesis of $EA_8C$ ..... 48
1.4.17 Synthesis of $RA_8C$ ..... 51
2 Self-assembly and hydrogel formation ..... 54
2.1 General methods ..... 54
2.1.1 XRD ..... 54
2.1.2 TEM imaging ..... 54
2.1.3 Cryo-TEM ..... 54
2.1.4 AFM imaging ..... 54
2.1.5 Rheology ..... 54
2.2 XRD of monosaccharide functionalized oligomers ..... 55
2.3 TEM images of monosaccharide functionalized oligomers before and after annealing ..... 56
2.4 AFM imaging of monosaccharide functionalized oligomers after annealing ..... 56
2.5 XRD of oligomers modified with C unit ..... 57
2.6 TEM of oligomers modified with C unit before and after annealing ..... 58
2.7 AFM imaging of oligomers modified with C unit after annealing ..... 59

2.8 Frequency sweeps of methylated cellulose oligomers ... 59
2.9 Recovery test of $CA_8C$ hydrogel ... 60
2.10 Frequency sweeps of $CA_8C$ hydrogels at different concentrations at different temperatures ... 60
2.11 Comparison of storage and loss moduli of $CA_8C$ hydrogels at different temperatures ... 61
2.12 Rheology profiles of $CA_8C$ 1.0 % (w/w) hydrogel at different temperatures ... 61
2.13 Rheology profiles of $CA_8C$ 2.0% (w/w) hydrogel at different temperatures ... 62
2.14 TEM of $CA_8C$ hydrogel at different concentrations. ... 62
2.15 Cryo-TEM of $CA_8C$ hydrogel ... 63
2.16 XRD of $XA_8C$ oligomers ... 64
2.17 AFM imaging of $XA_8C$ oligomers ... 65
2.18 Frequency sweep experiments of functionalized hydrogels ... 66
3 Biological assays ... 67
3.1.1 *C. albicans* strains and media ... 67
3.1.2 Filamentation assay ... 67
3.1.3 Viability assay ... 67
3.2 Filamentation assay (1-3 h) ... 67
3.3 Filamentation assay (6-8 h) ... 68
3.4 Filamentation assay 24 h ... 68
3.5 Filamentation assay (48 h) ... 69
3.6 Viability assay ... 69
4 References ... 70

# 1 Synthesis

## 1.1 General materials and methods

All chemicals used were reagent grade and used as supplied unless otherwise noted. The automated syntheses were performed on a home-built synthesizer developed at the Max Planck Institute of Colloids and Interfaces[1] or on commercial synthesizer Glyconeer 3.1 (GlycoUniverse, Germay). Analysis and purification by normal phase HPLC were performed by using an Agilent 1200 series. Products were lyophilized using a Christ Alpha 2-4 LD plus freeze dryer. $^{1}$H, $^{13}$C and HSQC NMR spectra were recorded on a Varian 400-NMR (400 MHz), or Varian 600-NMR (600 MHz) spectrometer. Spectra were recorded in $D_2O$ using the solvent as the internal standard in $^{1}$H NMR (D2O: 4.79 ppm $^{1}$H), concentration of samples was approx. 1 mg/mL. $^{1}$H NMR integrals of the resonances corresponding to residues at the reducing end are reported as non-integer numbers and the sum of the integrals of $\alpha$ and $\beta$ anomers, H-1 $\alpha$ and H-1 $\beta$ respectively, is set to 1. Residual solvent peaks in $^{1}$H NMR spectra: 8.4 ppm (formic acid), 1.8 ppm (acetonitrile). Weak intensity $^{13}$C resonances that were due to low solubility of compounds were derived from the respective HSQC cross peaks. High resolution mass spectra were obtained using a 6210 ESI-TOF mass spectrometer (Agilent) and a MALDI-TOF autoflex™ (Bruker).

## 1.2 Building blocks

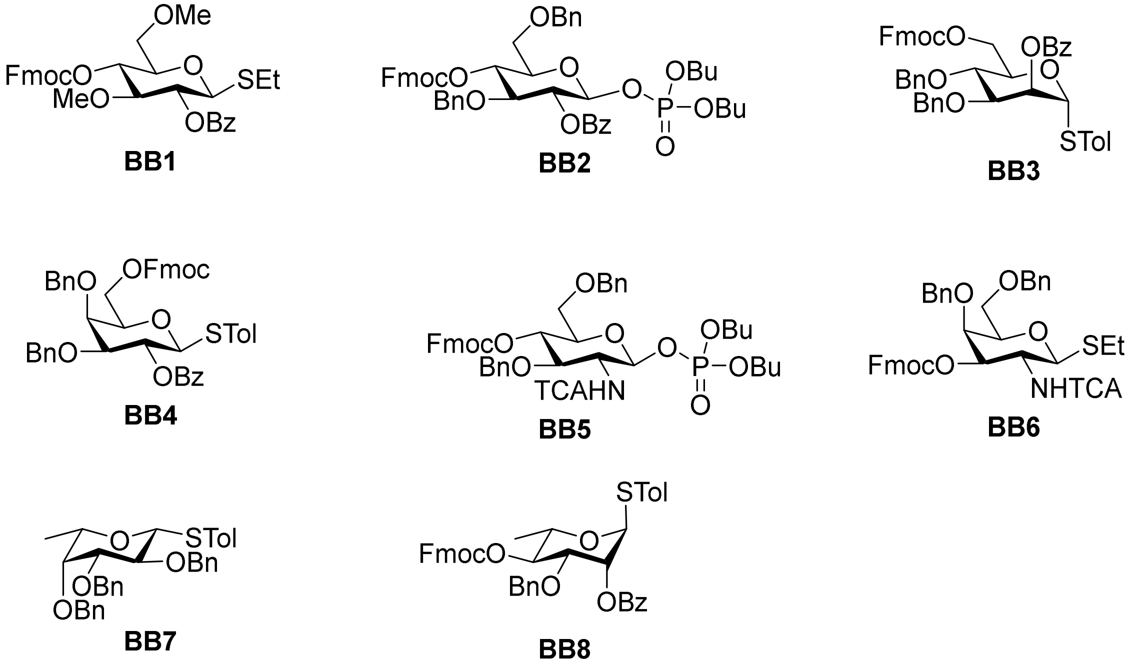


Figure S1 Structures of building blocks utilized for oligosaccharide synthesis.

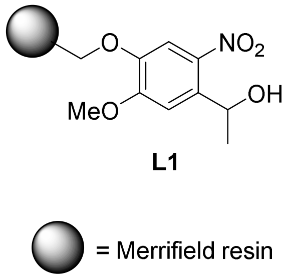


Figure S2 Structure of Merrifield resin equipped with a linker L1.

Building blocks BB1[2], BB2[3], BB5[4] and BB8[5] were synthesized according to previous literature procedures. Building blocks BB3, BB4, BB6 and BB7 were purchased from GlycoUniverse. Merrifield resin equipped with a photocleavable linker (L1, loading 0.35 mmol/g) was prepared according to previous literature.[6]

### 1.3 Automated Glycan Assembly

#### 1.3.1 General materials and methods

The automated syntheses were performed on a home-built synthesizer developed at the Max Planck Institute of Colloids and Interfaces[1] or on commercial synthesizer Glyconeer 3.1[7] (GlycoUniverse, Germay). All solvents used were HPLC-grade. The solvents used for the building blocks, activator, TMSOTf and capping solutions were taken from an anhydrous solvent system (J.C. Meyer). The building blocks were co-evaporated three times with toluene and dried on high vacuum before use. Oven-heated, argon-flushed flasks were used to prepare all moisture-sensitive solutions. Activator, capping, deprotection, acidic wash and building block solutions were freshly prepared and kept under argon during the automation run. All yields of products obtained by AGA were calculated on the basis of resin loading. Resin loading was determined following previously established procedures.[8]

#### 1.3.2 Preparation of stock solutions

- Building block solution: 0.06 mmol or 0.1 mmol of building block (see Module C) was dissolved in DCM (1 mL).
- NIS/TfOH activator solution: 1.35 g (6.0 mmol) of recrystallized NIS was dissolved in 40 mL of a 2:1 v/v mixture of anhydrous DCM and anhydrous dioxane. Then triflic acid (55 μL, 0.6 mmol) was added. The solution was kept at 0 °C (ice bath) for the duration of the automation run.
- Fmoc deprotection solution: a solution of 20% (v/v) piperidine in DMF was prepared.
- TMSOTf solution: TMSOTf (0.45 mL, 2.49 mmol) was added to DCM (40 mL).
- Capping solution: a solution of 10% (v/v) acetic anhydride and 2% (v/v) methanesulfonic acid in DCM was prepared.

#### 1.3.3 Modules for automated synthesis

Module A: Resin preparation

All automated syntheses were performed on 0.0125 mmol scales. Resin (L1, 36 mg) is placed in the reaction vessel and swollen in DCM for 20 min at RT prior to the synthesis. During this time, all reagent lines needed for the synthesis are washed and primed. After the swelling, the resin is washed with DMF, THF, and DCM (three times each with 2 mL for 25 s).

Module B: Acidic wash with TMSOTf solution (20 min)

The resin is swollen in 2 mL DCM and the temperature of the reaction vessel adjusted to -20 °C. Upon reaching the low temperature, TMSOTf solution (1 mL) is added dropwise to the reaction vessel. After bubbling for 3 min, the acidic solution is drained and the resin washed with 2 mL DCM for 25 s.

| Action | Cycles | Solution | Amount | T (°C) | Incubation time |
|---|---|---|---|---|---|
| Cooling | - | - | - | -20 | (15 min)* |
| Deliver | 1 | DCM | 2 mL | -20 | - |
| Deliver | 1 | TMSOTf solution | 1 mL | -20 | 3 min |
| Wash | 1 | DCM | 2 mL | -20 | 25 sec |

*Time required to reach the desired temperature.

Module C1: Thioglycoside Glycosylation (35 min)

The building block solution (0.10 mmol of BB in 1 mL of DCM per glycosylation) is delivered to the reaction vessel. After the set temperature is reached, the reaction is started by drop wise addition of the NIS/TfOH activator solution (1.0 mL, excess). The glycosylation conditions ($T_1$, $T_2$, $t_1$, and $t_2$) are building block dependent and are reported in a table below. After completion of the reaction, the solution is drained and the resin is

washed with DCM, DCM:dioxane (1:2, 3 mL for 20 s) and DCM (two times, each with 2 mL for 25 s). The temperature of the reaction vessel is increased to 25 °C for the next module.

| Action | Cycles | Solution | Amount | T (°C) | Incubation time |
|---|---|---|---|---|---|
| Cooling | - | - | - | $T_1$ | - |
| Deliver | 1 | BB solution | 1 mL | $T_1$ | - |
| Deliver | 1 | NIS/TfOH activator solution | 1 mL | $T_1$ | - |
| Reaction time (BB dependent) | 1 | - | - | $T_1$ to $T_2$ | $t_1$ $t_2$ |
| Wash | 1 | DCM | 2 mL | $T_2$ | 5 sec |
| Wash | 1 | DCM : Dioxane (1:2) | 2 mL | $T_2$ | 20 sec |
| Heating | - | - | - | 25 | - |
| Wash | 2 | DCM | 2 mL | > 0 | 25 sec |

The AGA glycosylation conditions employed for thioglycoside BBs were previously reported.[9]

| BB | Equiv. | $t_1$ (min) | $T_1$ (°C) | $t_2$ (min) | $T_2$ (°C) |
|---|---|---|---|---|---|
| BB1 | 6.5 | 5 | -20 | 20 | 0 |
| BB3 | 6.5 | 5 | -20 | 20 | 0 |
| BB4 | 6.5 | 5 | -20 | 20 | 0 |
| BB6 | 6.5 | 30 | -20 | 50 | 0 |
| BB7 | 6.5 | 5 | -40 | 20 | -20 |
| BB8 | 6.5 | 5 | -20 | 20 | 0 |

Module C2: Phosphate Glycosylation (45 min)

The building block solution (0.06 mmol of BB in 1 mL of DCM per glycosylation) is delivered to the reaction vessel. After the set temperature is reached, the reaction is started by drop wise addition of the TMSOTf solution (1.0 mL, stoichiometric). After completion of the reaction, the solution is drained and the resin washed with DCM (six times, each with 2 mL for 25 s). The temperature of the reaction vessel is increased to 25 °C for the next module.

| Action | Cycles | Solution | Amount | T (°C) | Incubation time |
|---|---|---|---|---|---|
| Cooling | - | - | - | $T_1$ | - |
| Deliver | 1 | BB solution | 1 mL | $T_1$ | - |
| Deliver | 1 | TMSOTf solution | 1 mL | $T_1$ | - |
| Reaction time (BB dependent) | 1 | | | $T_1$ to $T_2$ | $t_1$ $t_2$ min |
| Wash | 1 | DCM | 2 mL | $T_2$ | 5 sec |
| Heating | - | - | - | 25 | - |

| | | | | | |
|---|---|---|---|---|---|
| Wash | 6 | DCM | 2 mL | > 0 | 25 sec |

The AGA glycosylation conditions employed for the phosphate BB were previously reported. [9a]

| BB | Equiv. | $t_1$ (min) | $T_1$ (°C) | $t_2$ (min) | $T_2$ (°C) |
|---|---|---|---|---|---|
| BB2 | 3 | 5 | -30 | 40 | -10 |
| BB5 | 5 | 5 | -35 | 40 | -15 |

Module D: Capping (30 min)

The resin is washed with DMF (two times with 2 mL for 25 s) and the temperature of the reaction vessel adjusted to 25 °C. A pyridine solution (2 mL, 10%$_{v/v}$ in DMF) is delivered into the reaction vessel. After 1 min, the reaction solution is drained and the resin washed with DCM (three times with 3 mL for 25 s). Capping solution (4 mL) is delivered into the reaction vessel. After 20 min, the reaction solution is drained and the resin washed with DCM (three times with 3 mL for 25 s).

| Action | Cycles | Solution | Amount | T (°C) | Incubation time |
|---|---|---|---|---|---|
| Heating | - | - | - | 25 | (5 min)* |
| Wash | 2 | DMF | 2 mL | 25 | 25 sec |
| Deliver | 1 | 10% Pyridine in DMF | 2 mL | 25 | 1 min |
| Wash | 3 | DCM | 2 mL | 25 | 25 sec |
| Deliver | 1 | Capping Solution | 4 mL | 25 | 20 min |
| Wash | 3 | DCM | 2 mL | 25 | 25 sec |

*Time required to reach the desired temperature.

Module E1: Fmoc deprotection (9 min)

The resin is washed with DMF (three times with 2 mL for 25 s) and the temperature of the reaction vessel adjusted to 25 °C. Fmoc deprotection solution (2mL) is delivered to the reaction vessel and kept under Ar bubbling. After 5 min, the reaction solution is drained and the resin washed with DMF (three times with 3 mL for 25 s) and DCM (five times each with 2 mL for 25 s). The temperature of the reaction vessel is decreased to -20 °C for the next module.

| Action | Cycles | Solution | Amount | T (°C) | Incubation time |
|---|---|---|---|---|---|
| Wash | 3 | DMF | 2 mL | 25 | 25 sec |
| Deliver | 1 | Fmoc depr. Solution | 2 mL | 25 | 5 min |
| Wash | 1 | DMF | 2 mL | | |
| Cooling | - | - | - | -20 | - |
| Wash | 3 | DMF | 2 mL | < 25 | 25 sec |
| Wash | 5 | DCM | 2 mL | < 25 | 25 sec |

### 1.3.4 Post-AGA manipulations

#### Module F: On-resin methanolysis

The resin is suspended in THF (4 mL). MeONa in MeOH (0.5 M, 0.4 mL) is added and the suspension is gently shaken at room temperature. After micro-cleavage (see *Module G2*) indicates the complete removal of benzoyl groups, the resin is repeatedly washed with MeOH (3 x 2 mL) and DCM (3 x 2 mL).

#### Module F1: On-resin *N*-acetylation ($QA_8C$)

The resin is suspended in a 4 mL solution of acetic anhydride in DMF (15% v/v) and the mixture gently shaken at rt for 3 h, after which time the resin is repeatedly washed with DMF (5 x 4 mL), MeOH (5 x 4 mL) and $CH_2Cl_2$ (5 x 4 mL). The resin is suspended in THF (4 mL). MeONa in MeOH (0.5 M, 0.4 mL) is added and the suspension is gently shaken at room temperature for 2 h to selectively cleave any residual *O*-acetyls.[10]

#### Module G1: Cleavage from solid support

The oligosaccharides are cleaved from the solid support using a continuous-flow photoreactor as described previously.[11]

#### Module G2: Micro-cleavage from solid support

Trace amount of resin (around 20 beads) is dispersed in DCM (0.1 mL) and irradiated with a UV lamp (6 W, 356 nm) for 10 minutes. ACN (10 µL) is then added to the resin and the resulting solution analyzed by MALDI.

#### Module H: Hydrogenolysis at ambient pressure

The crude compound obtained from *Module G1* is dissolved in 2 mL of EtOAc:*t*BuOH:$H_2O$ (2:1:1). 100% by weight Pd/C (10% (w/w)) is added to the stirred flask, the reaction purged for 5 min with a $N_2$ balloon, and then equipped with a $H_2$ balloon. The reaction progress is monitored to avoid undesired side products formation (*i.e.* degradation of reducing end).[9b] Upon completion, the reaction is filtered (PTFE 0.45 µm 25 mm syringe filter, Fisher scientific) and washed with EtOAc, $H_2O$, and ACN (4 mL each). The filtrates are concentrated *in vacuo.*

#### Module I: Purification

The purification of the crudes was conducted using a $C_{18}$ silica column (Method C) or reverse phase HPLC (Agilent 1200 Series, Method B). The pure compounds were analyzed using analytical HPLC (Agilent 1200 Series, Method A).

- Method A: (Synergi Hydro RP18 column, Phenomenex, 250 x 4.6 mm), flow rate of 1.0 mL/min with $H_2O$ (0.1% formic acid) and ACN as eluents [isocratic (5 min), linear gradient to 30% ACN (30 min), linear gradient to 100% ACN (5 min), isocratic 100% ACN (5 min)].
- Method B: (Synergi Hydro RP18 column, Phenomenex, 250 x 10 mm) flow rate of 4.0 mL/min with $H_2O$ (0.1% formic acid) and ACN as eluents [isocratic (5 min), linear gradient to 30% ACN (30 min), linear gradient to 100% ACN (5 min), isocratic 100% ACN (5 min)].
- Method C: (Manual reverse phase $C_{18}$ silica gel column chromatography): $H_2O$ (0.1% formic acid, 10 mL), 3% MeOH (10 mL), 6% MeOH (10 mL), 9% MeOH (10 mL), 15% MeOH (10 mL).

Following final purification, all deprotected products are lyophilized on a Christ Alpha 2-4 LD plus freeze dryer prior to characterization.

## 1.4 Oligosaccharide synthesis

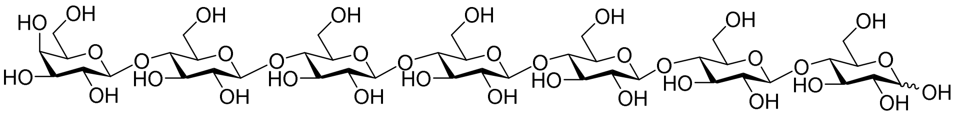

**GA$_6$**

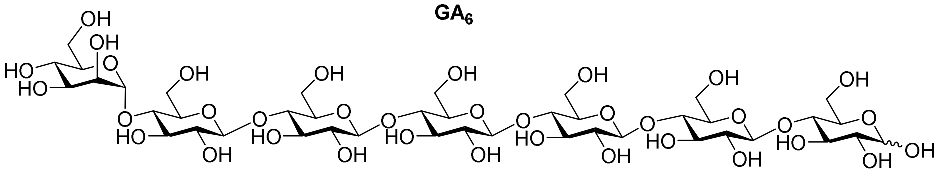

**MA$_6$**

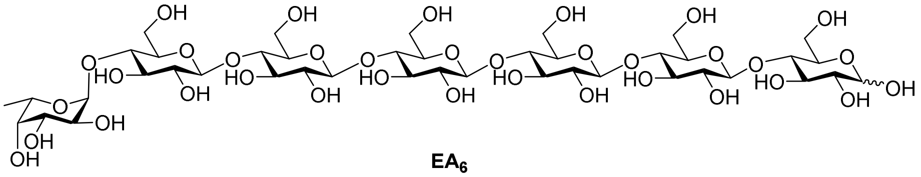

**EA$_6$**

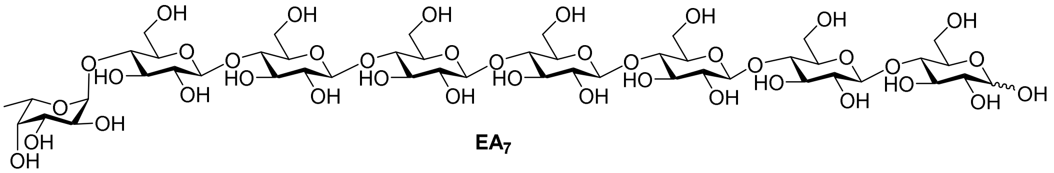

**EA$_7$**

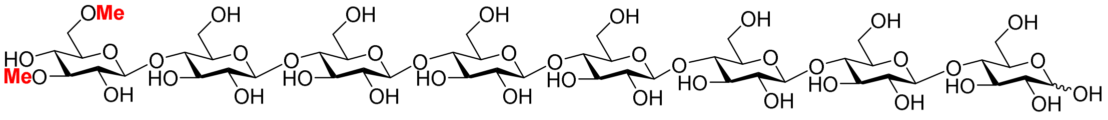

**CA$_7$**

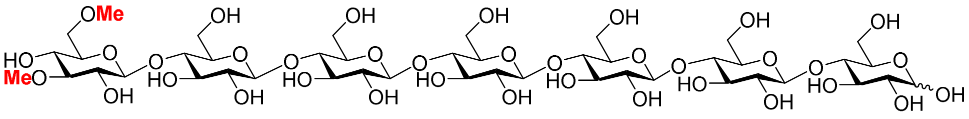

**CA$_6$**

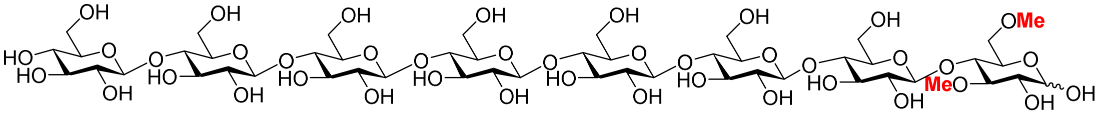

**A$_7$C**

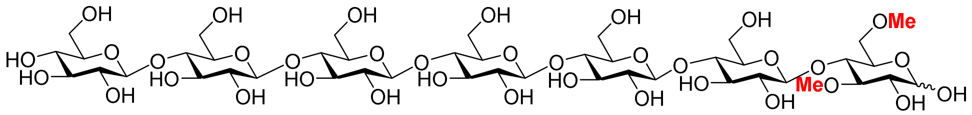

**A$_6$C**

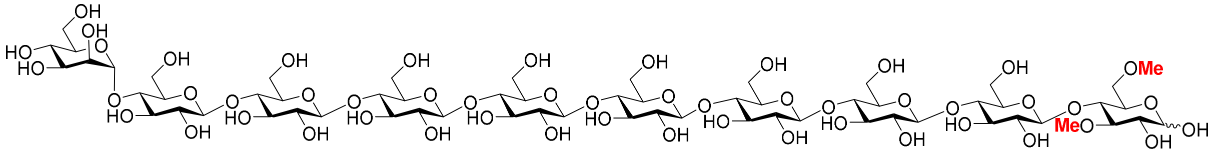

**MA$_8$C**

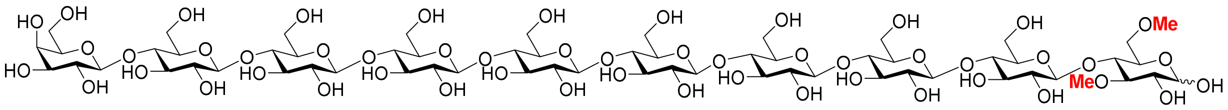

**GA$_8$C**

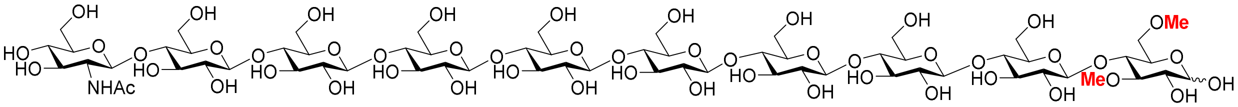

**NA$_8$C**

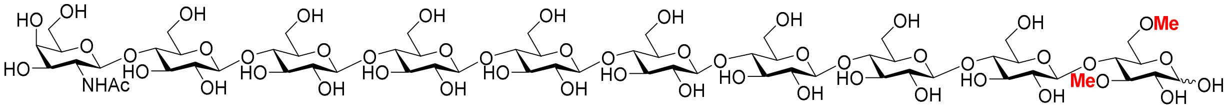

**QA$_8$C**

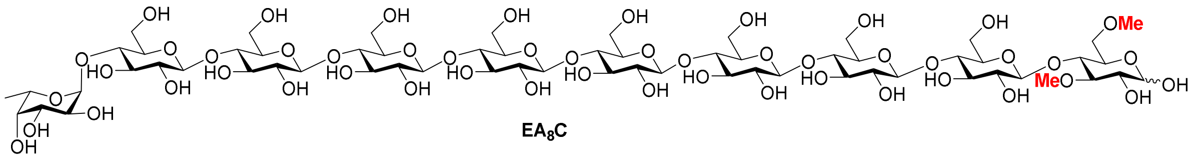

**EA$_8$C**

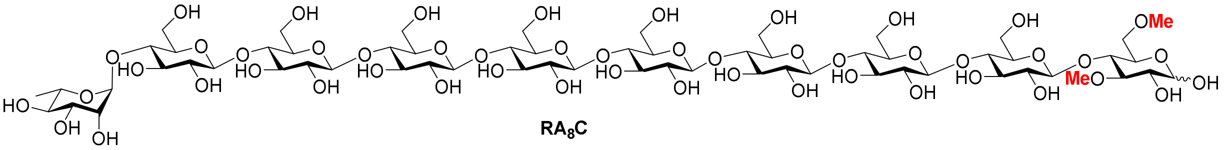

**RA$_8$C**

Figure S3 Structures of oligosaccharides synthesized by AGA in this work. Cellulose hexamer A$_6$[12] and decamer CA$_8$C[13] were synthesized as previously reported.

### 1.4.1 Synthesis of $GA_6$

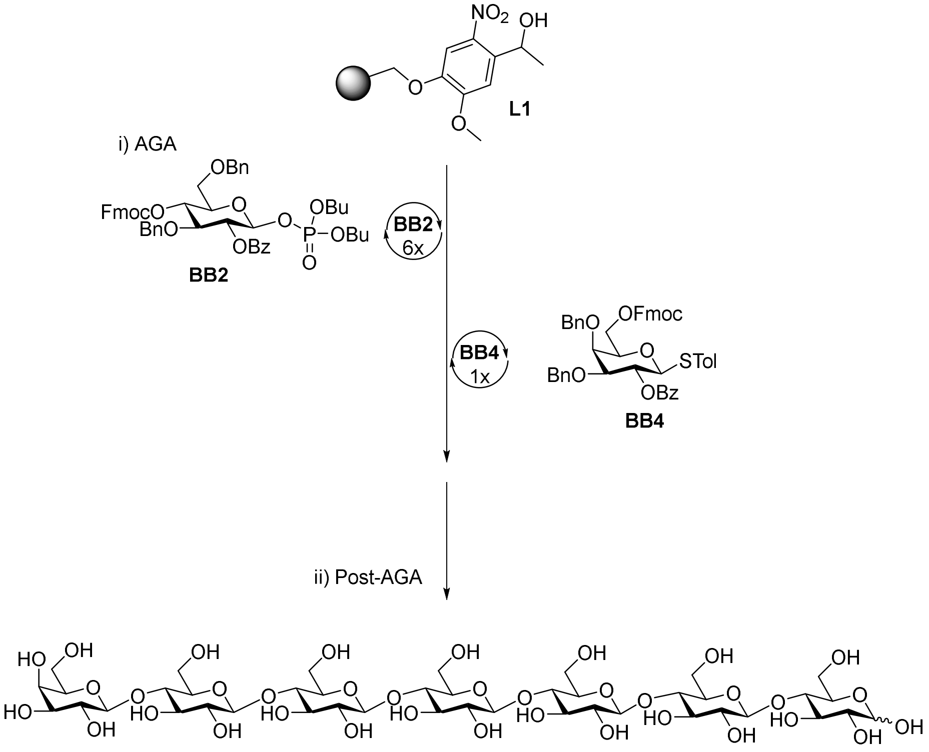


**$GA_6$**

| Step | BB | Modules | Notes |
|---|---|---|---|
| AGA | | A | L1 swelling |
| | (BB1)x6 | (B, C1, D, E1)x6 | C2 (BB1, -30°C for 5 min, -10°C for 40 min) |
| | BB4 | B, C1, D, E1 | C1: (BB4, -20°C for 5 min, 0°C for 20 min) |
| Post-AGA | | F, G, H, I | F: (24 h)<br>H: (16 h)<br>I: C |

Automated synthesis, global deprotection, and purification afforded compound $GA_6$ as a white solid (3.3 mg, 23% overall yield).

Analytical data for $GA_6$:

$^{1}H$ NMR (600 MHz, $D_2O$) $\delta$ 5.21 (d, $J$ = 3.8 Hz, 0.4H, H1-$\alpha$), 4.65 (d, $J$ = 8.0 Hz, 0.6H, H1-$\beta$), 4.52 (d, $J$ = 8.1 Hz, 5H), 4.44 (d, $J$ = 7.8 Hz, 1H), 3.99 - 3.90 (m, 7H), 3.86 - 3.70 (m, 10H), 3.69 - 3.57 (m, 18H), 3.55 – 3.48 (m, 1H), 3.37 - 3.25 (m, 6H). $^{13}C$ NMR (151 MHz, $D_2O$) $\delta$ 102.30, 78.18, 75.31, 74.77, 74.06, 73.93, 72.87, 59.78. (ESI-HRMS) $m/z$ 1153.387 $[M + H]^+$ ($C_{42}H_{73}O_{36}$ requires 1153.387).

RP-HPLC of $GA_6$ (ELSD trace, Method A, $t_R$ = 14.4 min)

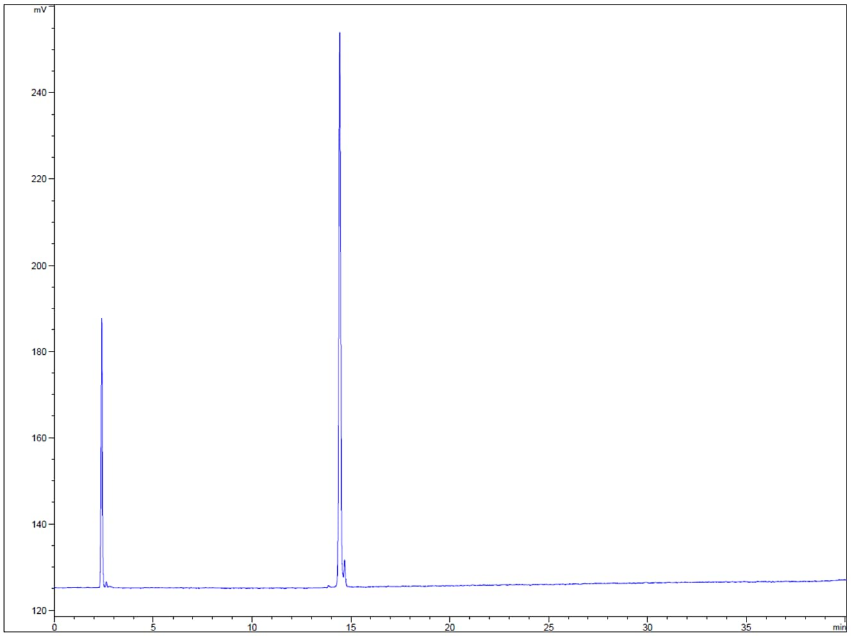


$^{1}$H NMR of $GA_6$ (600 MHz, $D_2O$)

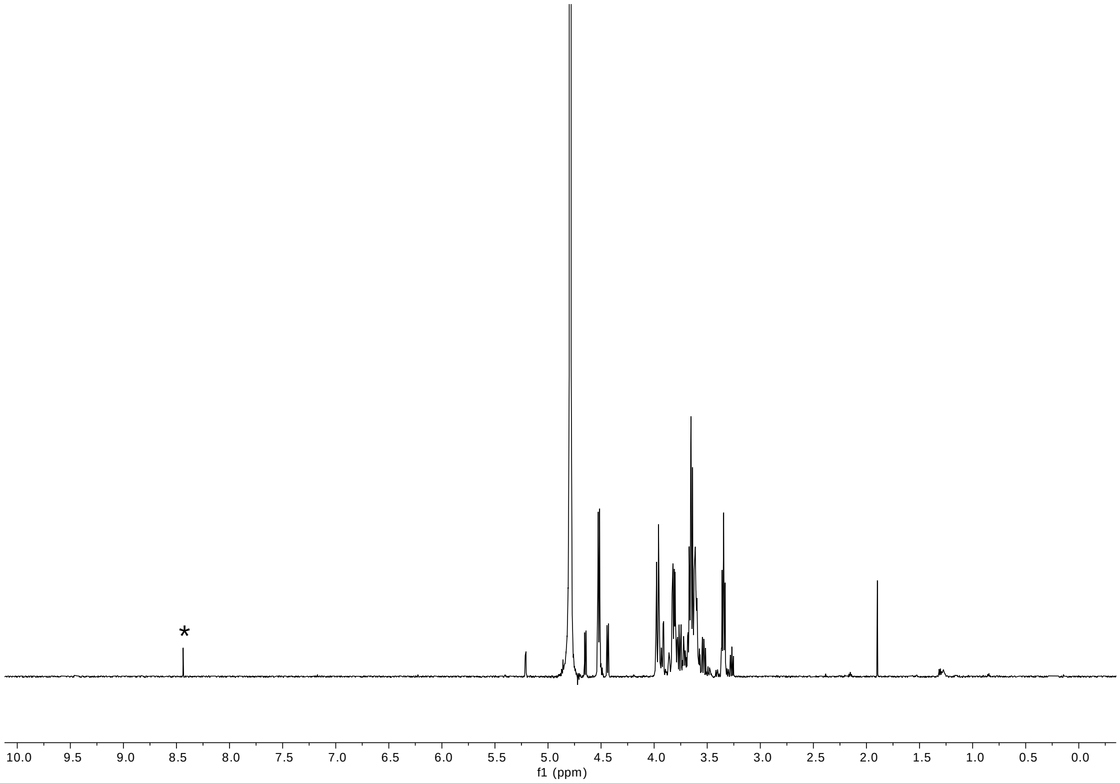


*Residual formic acid

HSQC NMR of GA$_6$ ($D_2O$)

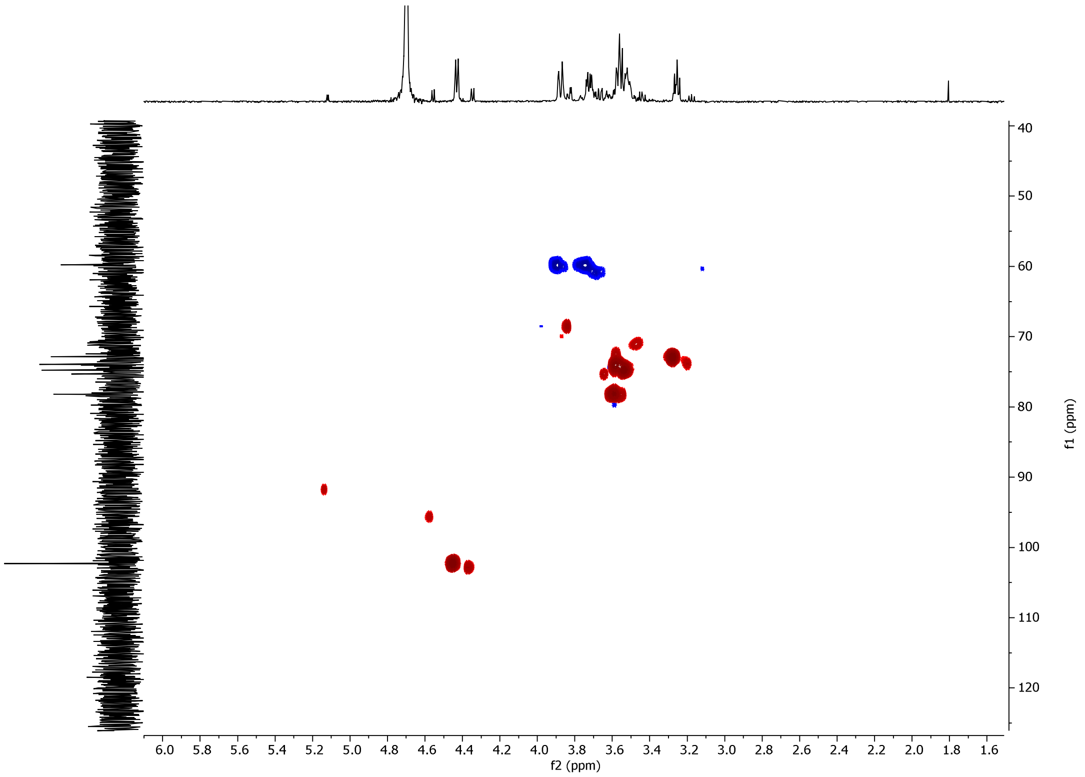

### 1.4.2 Synthesis of $MA_6$

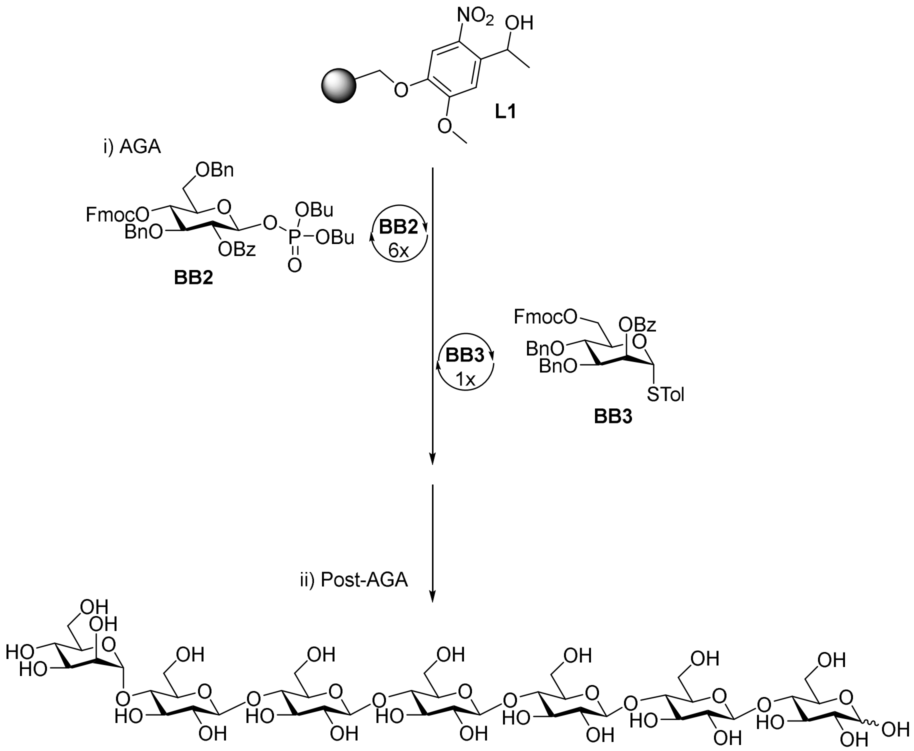


$MA_6$

| Step | BB | Modules | Notes |
|---|---|---|---|
| AGA | | A | L1 swelling |
| | (BB1)x6 | (B, C1, D, E1)x6 | C2 (BB1, -30°C for 5 min, -10°C for 40 min) |
| | BB3 | B, C1, D, E1 | C1: (BB3, -40°C for 5 min, -20°C for 20 min) |
| Post-AGA | | F, G, H, I | F: (24 h) |
| | | | H: (16 h) |
| | | | I: C |

Automated synthesis, global deprotection, and purification afforded compound $MA_6$ as a white solid (6.1 mg, 53% overall yield).

Analytical data for $MA_6$:

$^1$H NMR (600 MHz, $D_2O$) δ 5.24 (d, $J$ = 1.8 Hz, 1H), 5.18 (d, $J$ = 3.8 Hz, 0.4H, H1-α), 4.61 (d, $J$ = 8.0 Hz, 0.6H, H1-β), 4.49 (d, $J$ = 7.9 Hz, 4H), 4.45 (d, $J$ = 7.9 Hz, 1H), 4.01 (dd, $J$ = 3.3, 1.9 Hz, 1H), 3.95 - 3.91 (m, 4H), 3.89 (dd, $J$ = 7.7, 2.3 Hz, 1H), 3.86 (dd, $J$ = 8.9, 1.9 Hz, 1H), 3.83 (d, $J$ = 2.0 Hz, 1H), 3.80 - 3.69 (m, 8H), 3.65 - 3.52 (m, 21H), 3.33 - 3.29 (m, 4H), 3.28 - 3.22 (m, 1H). $^{13}$C NMR (151 MHz, $D_2O$) δ 102.37, 102.30, 101.30, 78.18, 75.92, 74.77, 74.60, 73.94, 73.68, 73.22, 72.88, 70.20, 66.48, 60.86, 59.79. (ESI-HRMS) m/z 1153.384 [M + H]$^+$ ($C_{42}H_{73}O_{36}$ requires 1153.387).

RP-HPLC of $MA_6$ (ELSD trace, Method A, $t_R$ = 15.7 min)

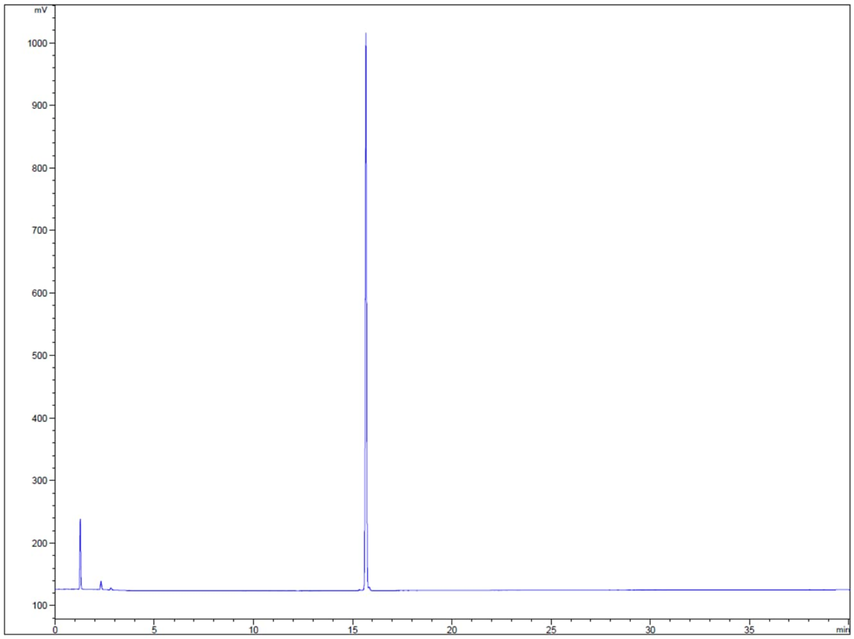


$^1H$ NMR of $MA_6$ (600 MHz, $D_2O$)

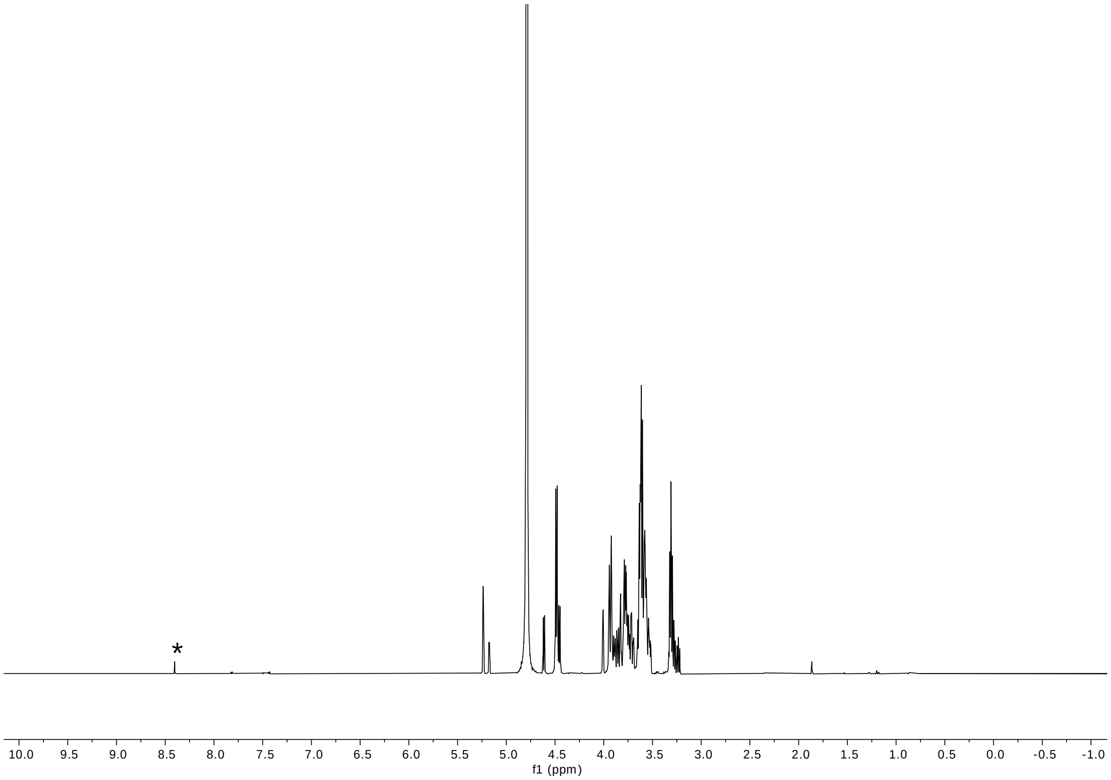


*Residual formic acid

HSQC NMR of $MA_6$ ($D_2O$)

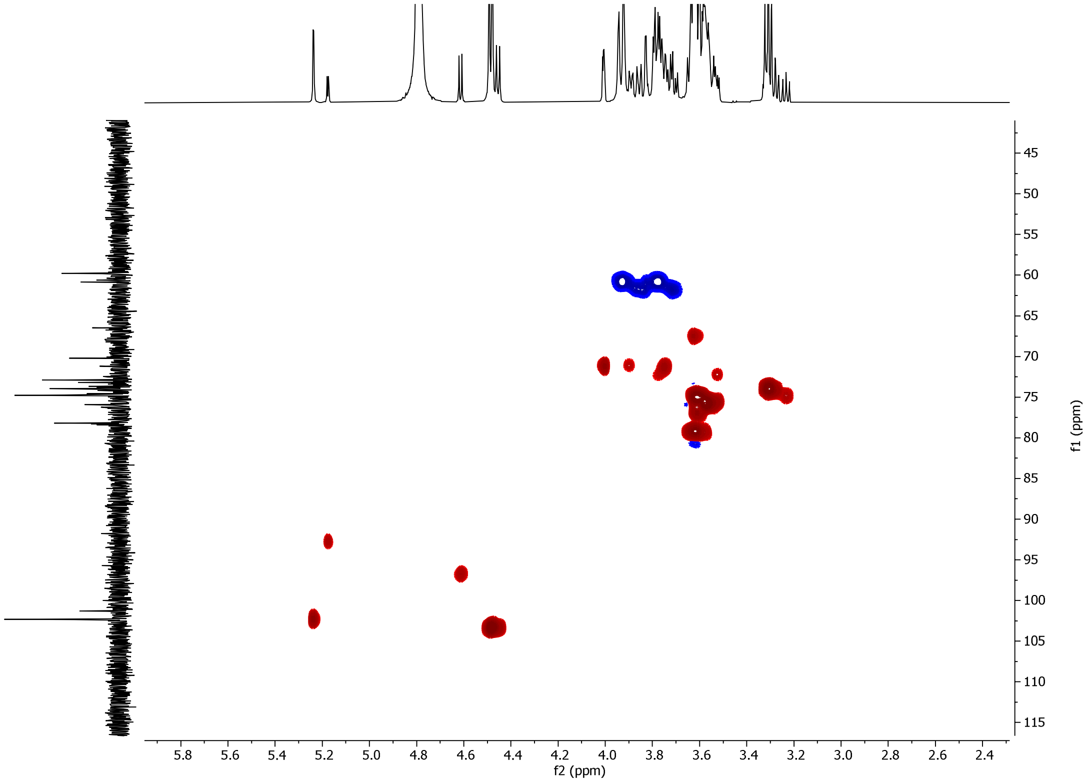

### 1.4.4 Synthesis of $EA_6$

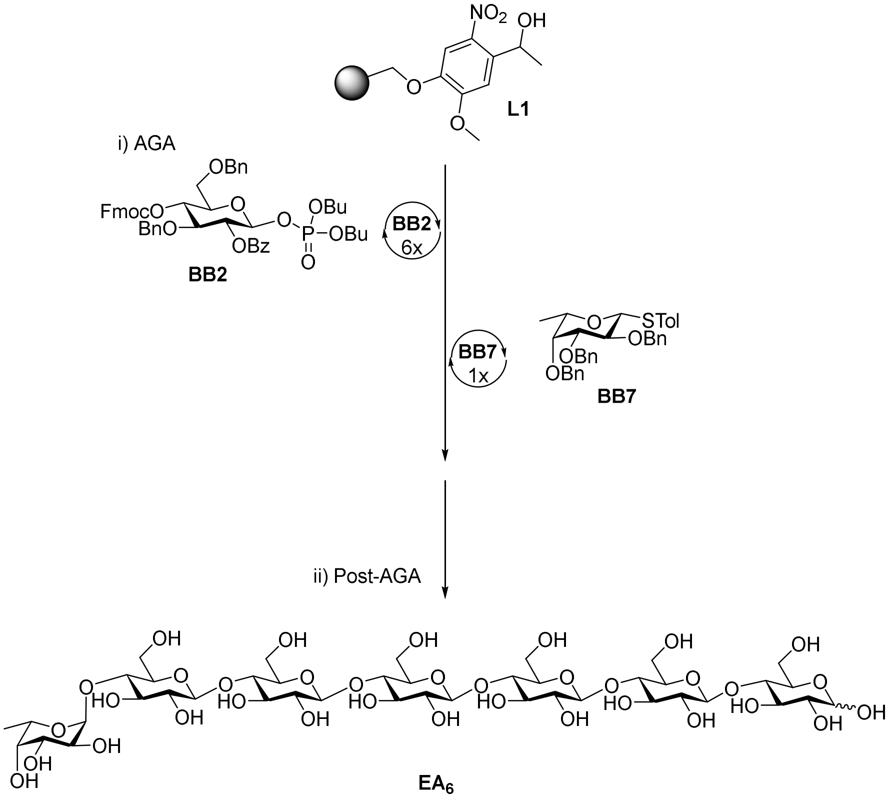


**$EA_6$**

| Step | BB | Modules | Notes |
|---|---|---|---|
| AGA | | A | L1 swelling |
| | (BB1)x6 | (B, C1, D, E1)x6 | C2 (BB1, -30°C for 5 min, -10°C for 40 min) |
| | BB7 | B, C1, D, E1 | C1: (BB7, -40°C for 5 min, -20°C for 20 min) |
| Post-AGA | | F, G, H, I | F: (24 h)<br>H: (16 h)<br>I: C |

Automated synthesis, global deprotection, and purification afforded compound $EA_6$ as a white solid (7.5 mg, 52% overall yield).

Analytical data for $EA_6$:

$^1$H NMR (600 MHz, $D_2O$) δ 5.18 (d, $J$ = 3.7 Hz, 0.3H, H1-α), 4.89 (d, $J$ = 4.1 Hz, 1H), 4.61 (d, $J$ = 8.0 Hz, 0.7H, H1-β), 4.51 - 4.45 (m, 5H), 4.36 - 4.30 (m, 1H), 3.96 - 3.88 (m, 6H), 3.83 - 3.72 (m, 10H), 3.65 - 3.47 (m, 18H), 3.34 - 3.20 (m, 6H), 1.13 (d, $J$ = 6.6 Hz, 3H). $^{13}$C NMR (151 MHz, $D_2O$) δ 102.36, 102.27, 99.45, 95.68, 91.74, 78.48, 78.33, 78.22, 78.12, 76.66, 75.22, 74.73, 74.15, 74.11, 73.89, 73.80, 73.46, 72.84, 71.80, 71.21, 71.13, 70.03, 69.28, 67.97, 66.89, 59.88, 59.73, 15.13. (ESI-HRMS) m/z 1137.391 [M + H]$^+$ ($C_{42}H_{73}O_{35}$ requires 1137.392).

RP-HPLC of $EA_6$ (ELSD trace, Method A, $t_R$ = 15.6 min)

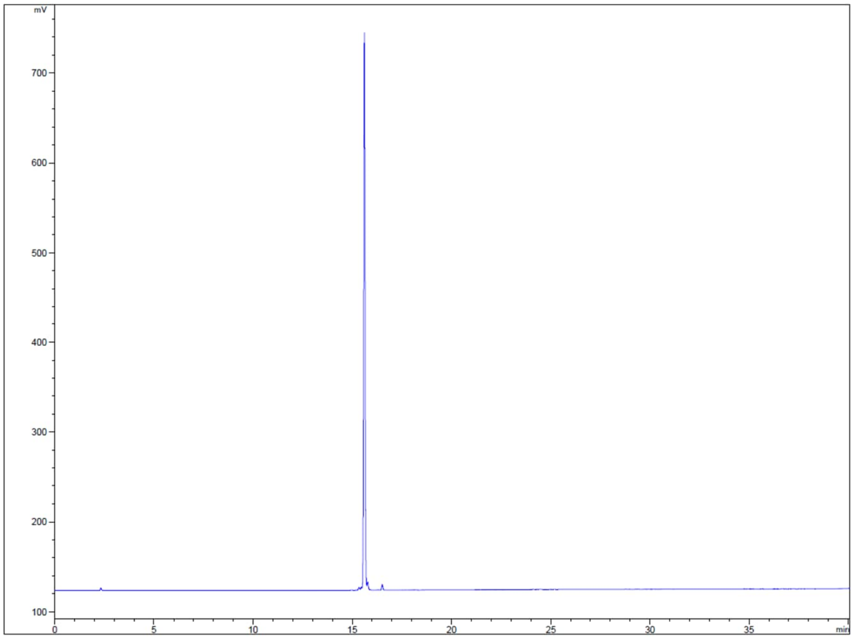


$^1H$ NMR of $EA_6$ (600 MHz, $D_2O$)

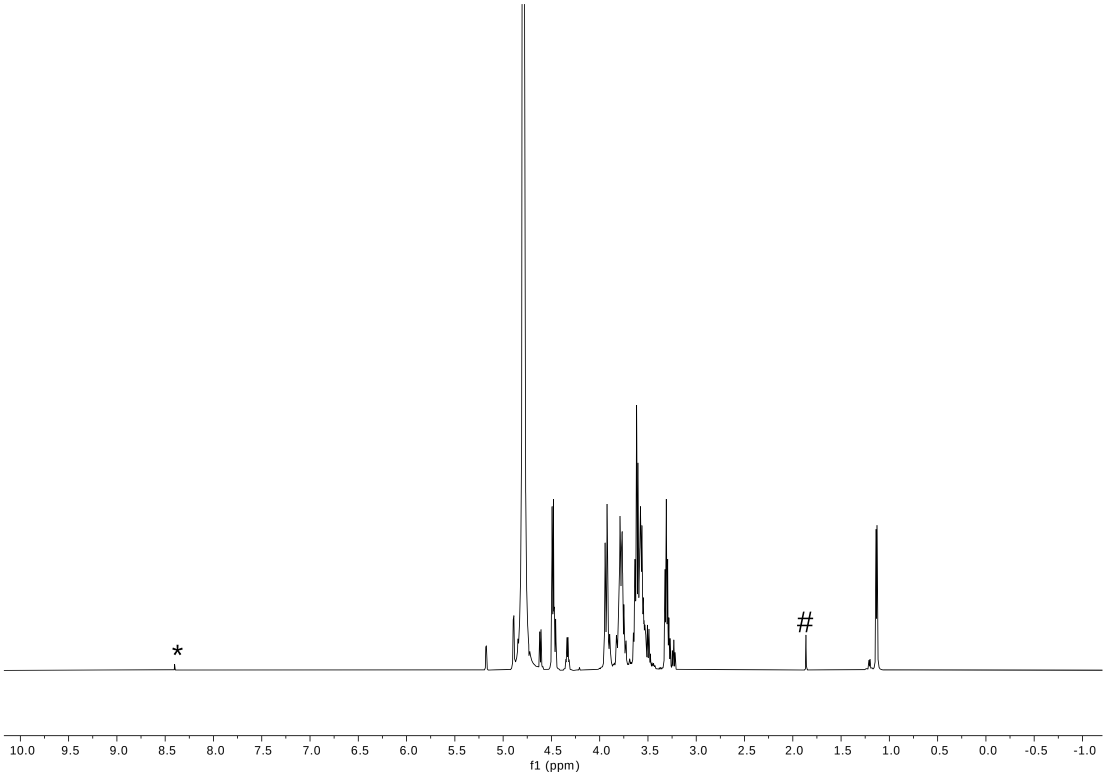


*Residual formic acid

# Residual acetonitrile

HSQC NMR of $EA_6$ ($D_2O$)

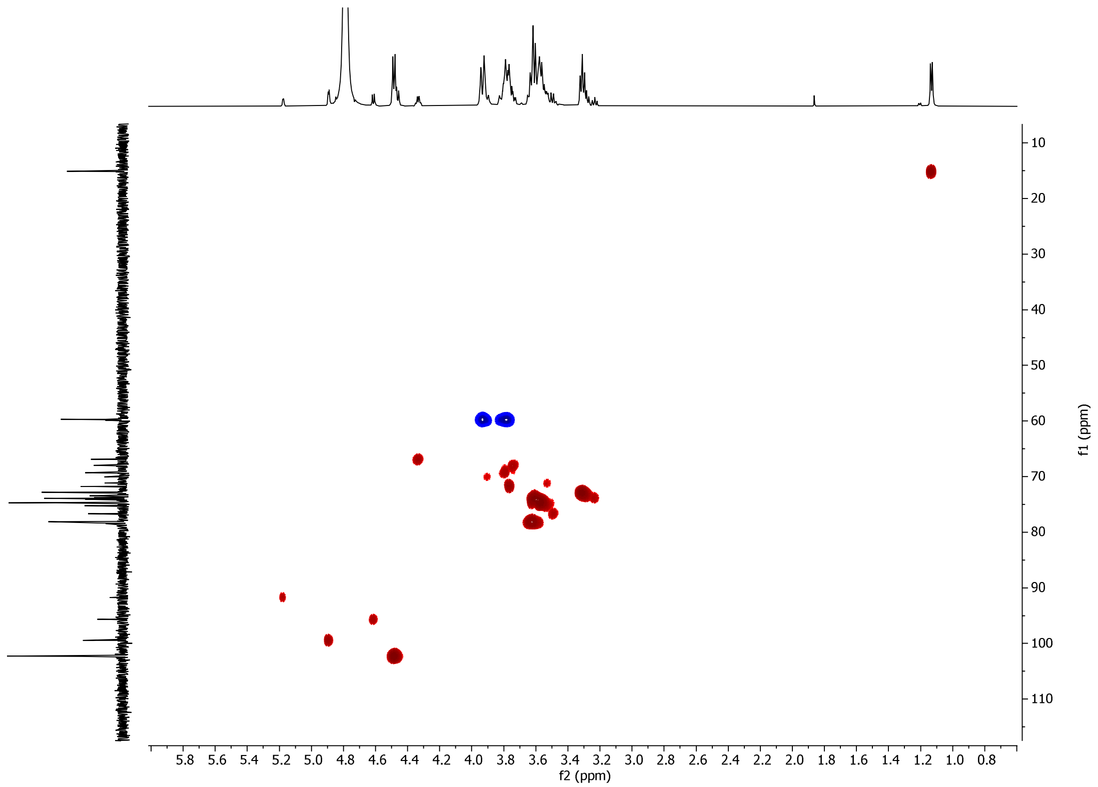

### 1.4.5 Synthesis of $EA_7$

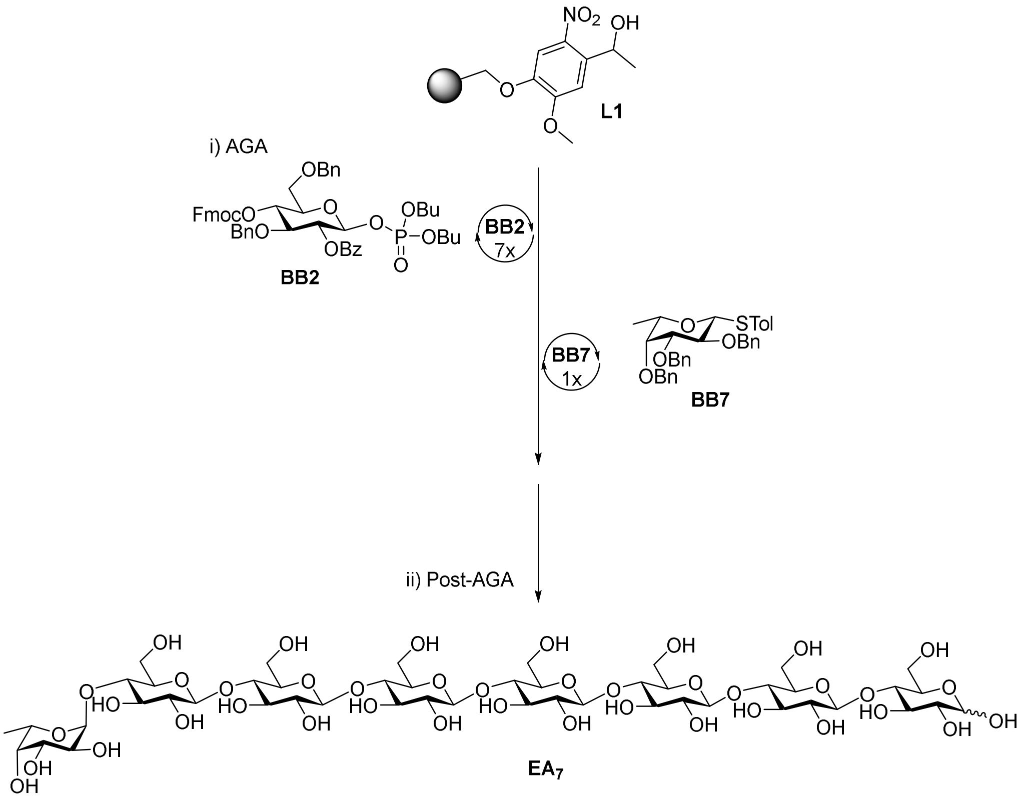


| Step | BB | Modules | Notes |
|---|---|---|---|
| AGA | | A | L1 swelling |
| | (BB1)x7 | (B, C1, D, E1)x7 | C2 (BB1, -30°C for 5 min, -10°C for 40 min) |
| | BB7 | B, C1, D, E1 | C1: (BB7, -40°C for 5 min, -20°C for 20 min) |
| Post-AGA | | F, G, H, I | F: (24 h)<br>H: (16 h)<br>I: C |

Automated synthesis, global deprotection, and purification afforded compound $EA_7$ as a white solid (4.2 mg, 26% overall yield).

Analytical data for $EA_7$:

$^{1}$H NMR (400 MHz, $D_2O$) δ 5.23 (d, *J* = 3.7 Hz, 0.4H, H1-α), 4.95 (d, *J* = 3.9 Hz, 1H), 4.67 (d, *J* = 8.0 Hz, 0.6H, H1-β), 4.53 (d, *J* = 8.6 Hz, 6H), 4.38 (m, 1H), 3.99 (m, 6H), 3.83 (m, 12H), 3.72 - 3.55 (m, 21H), 3.42 - 3.31 (m, 6H), 1.19 (d, J = 6.5 Hz, 3H). $^{13}$C NMR (100 MHz, $D_2O$) δ 102.28, 99.22, 78.11, 74.08, 72.95, 71.66, 68.28, 66.99, 59.74, 59.74, 15.10. (ESI-HRMS) m/z 1299.448 $[M + H]^+$ ($C_{48}H_{83}O_{40}$ requires 1299.445).

RP-HPLC of $EA_7$ (ELSD trace, Method A, $t_R$ = 14.9 min)

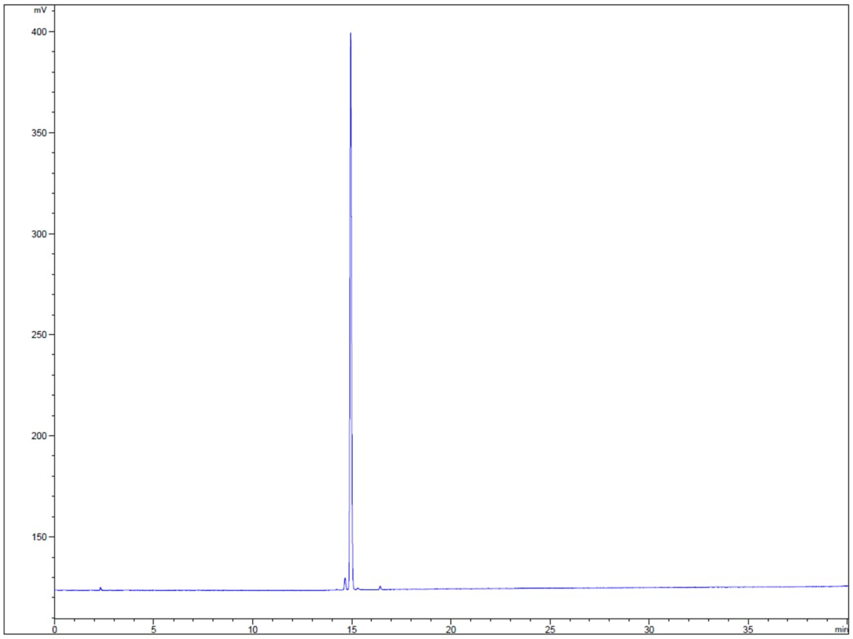

$^1H$ NMR of $EA_7$ (400 MHz, $D_2O$)

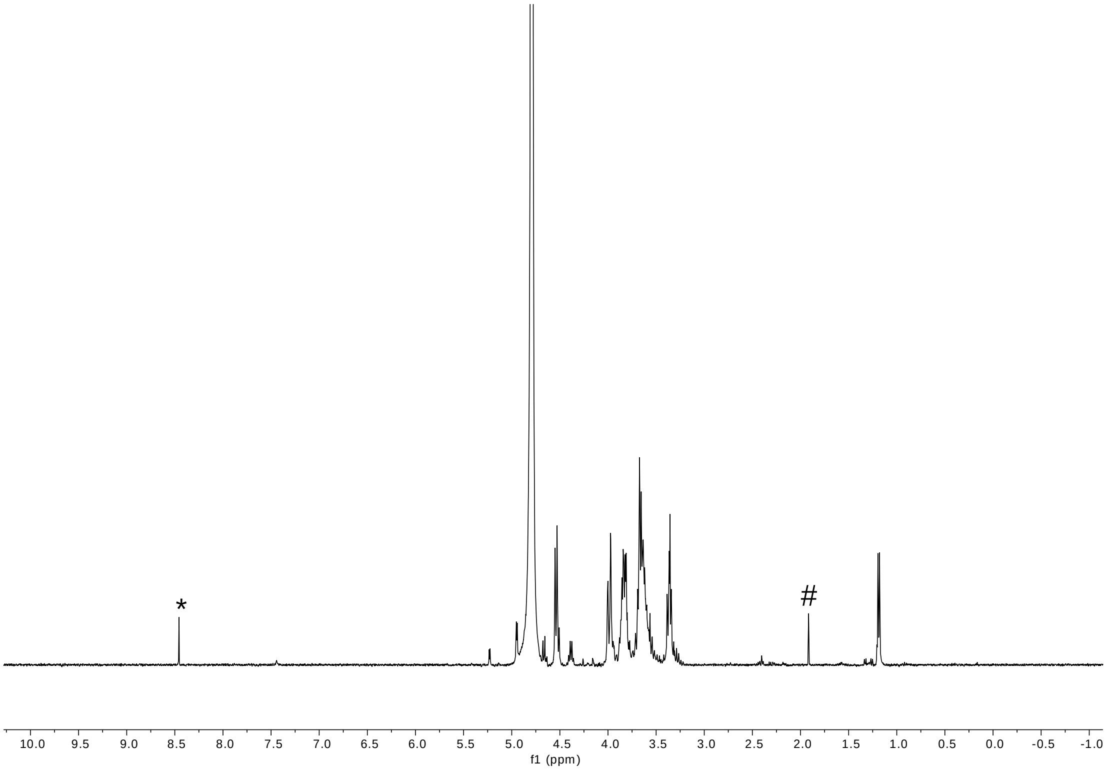


*Residual formic acid
# Residual acetonitrile

HSQC NMR of $EA_7$ ($D_2O$)

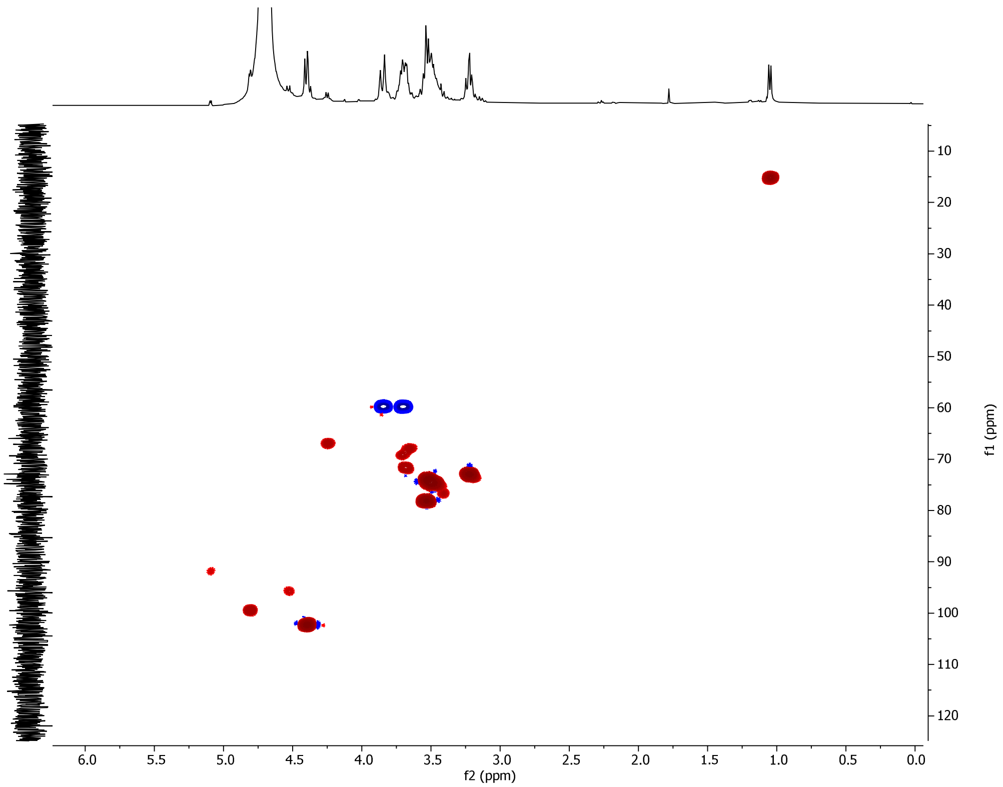

### 1.4.6 Synthesis of $CA_7$

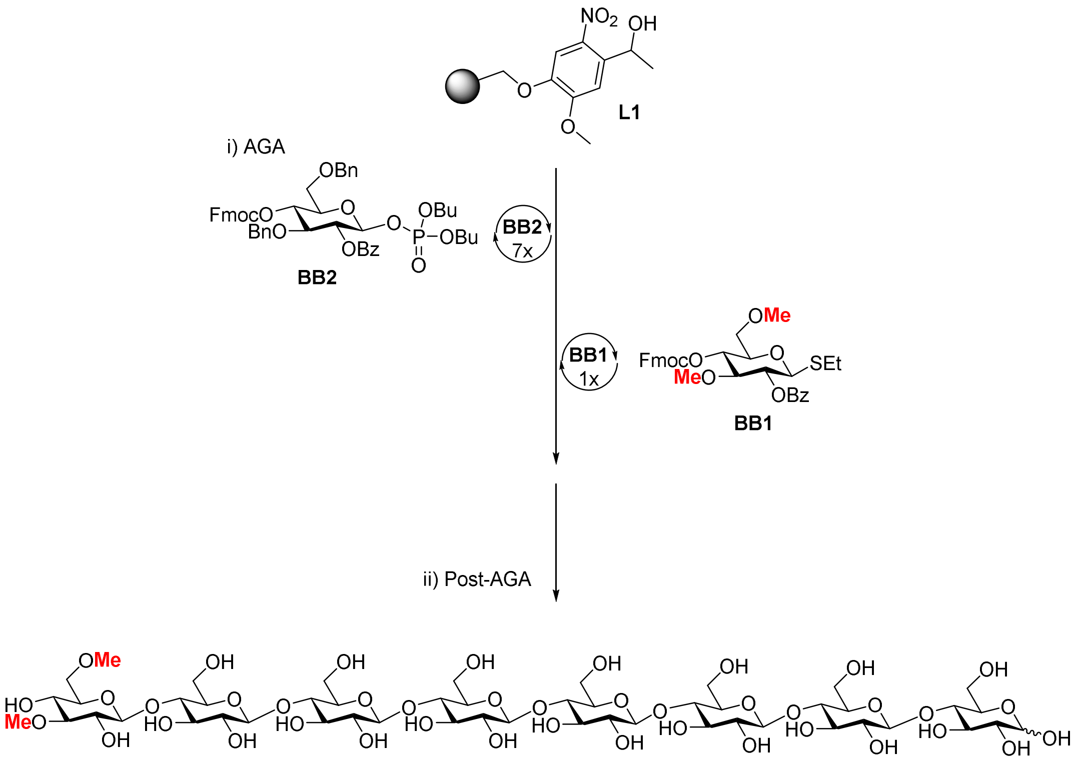


**$CA_7$**

| Step | BB | Modules | Notes |
|---|---|---|---|
| AGA | | A | L1 swelling |
| | (BB1)x7 | (B, C1, D, E1)x7 | C2 (BB1, -30°C for 5 min, -10°C for 40 min) |
| | BB2 | B, C1, D, E1 | C1: (BB2, -20°C for 5 min, 0°C for 40 min) |
| Post-AGA | | F, G, H, I | F: (24 h) |
| | | | H: (16 h) |
| | | | I: C |

Automated synthesis, global deprotection, and purification afforded compound $CA_7$ as a white solid (2.8 mg, 17% overall yield).

Analytical data for $CA_7$:

$^1$H NMR (400 MHz, $D_2O$) $\delta$ 5.16 (d, $J$ = 3.7 Hz, 0.4 H, H1-$\alpha$), 4.60 (d, $J$ = 8.0 Hz, 0.6 H, H1-$\beta$), 4.50 - 4.42 (m, 7H), 3.95 - 3.86 (m, 6H), 3.83 - 3.72 (m, 6H), 3.71 - 3.67 (m, 1H), 3.64 - 3.51 (m, 27H), 3.48 - 3.41 (m, 1H), 3.35 - 3.19 (m, 13H). $^{13}$C NMR (101 MHz, $D_2O$) $\delta$ 102.25, 95.64, 91.77, 78.06, 74.35, 74.03, 72.74, 70.81, 70.64, 68.87, 59.84, 59.84, 59.68, 58.55. (ESI-HRMS) m/z 1343.473 [M + H]$^+$ ($C_{50}H_{87}O_{41}$ requires 1343.471).

RP-HPLC of $CA_7$ (ELSD trace, Method A, $t_R$ = 18.1 min)

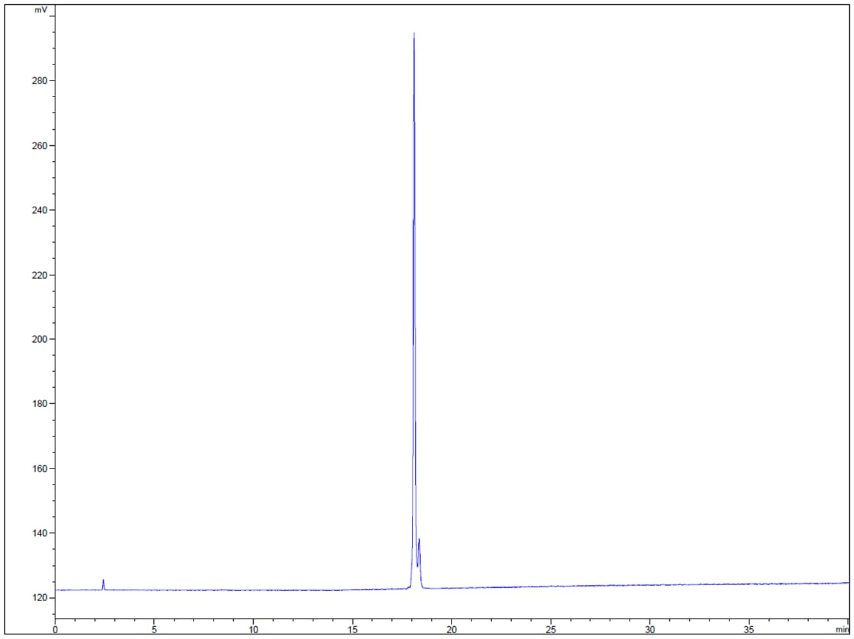


$^1H$ NMR of $CA_7$ (400 MHz, $D_2O$)

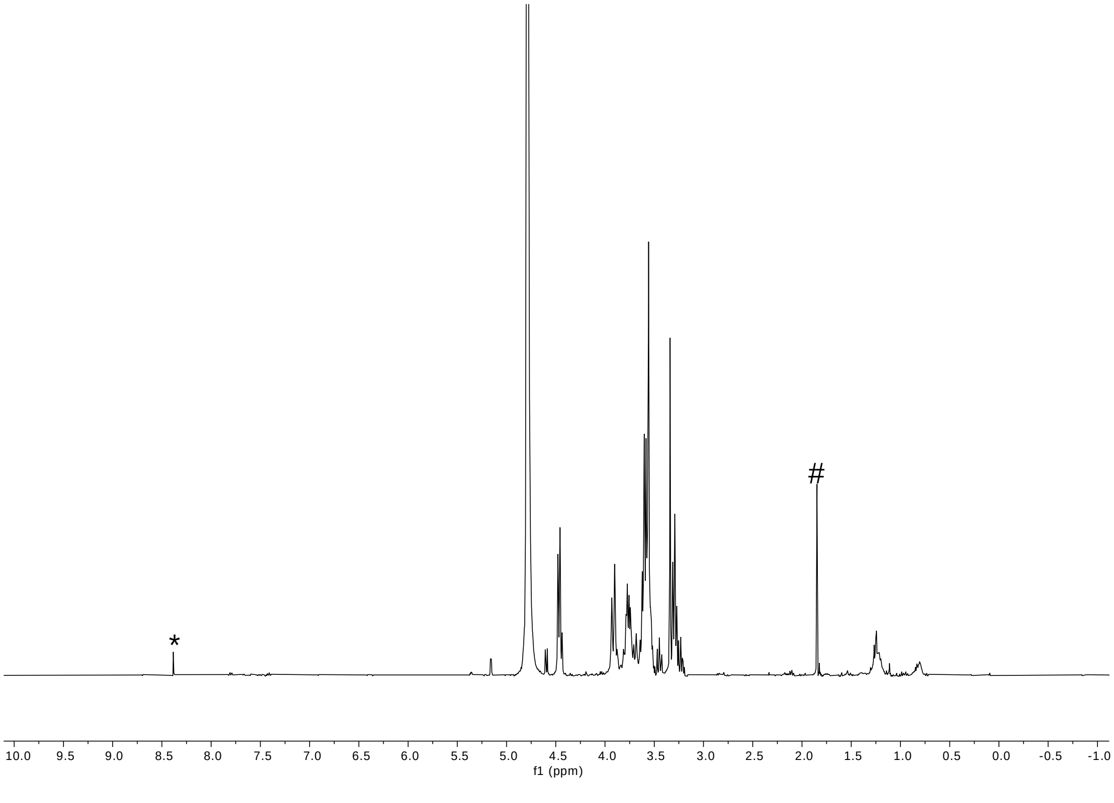


*Residual formic acid

# Residual acetonitrile

HSQC NMR of $CA_7$ ($D_2O$)

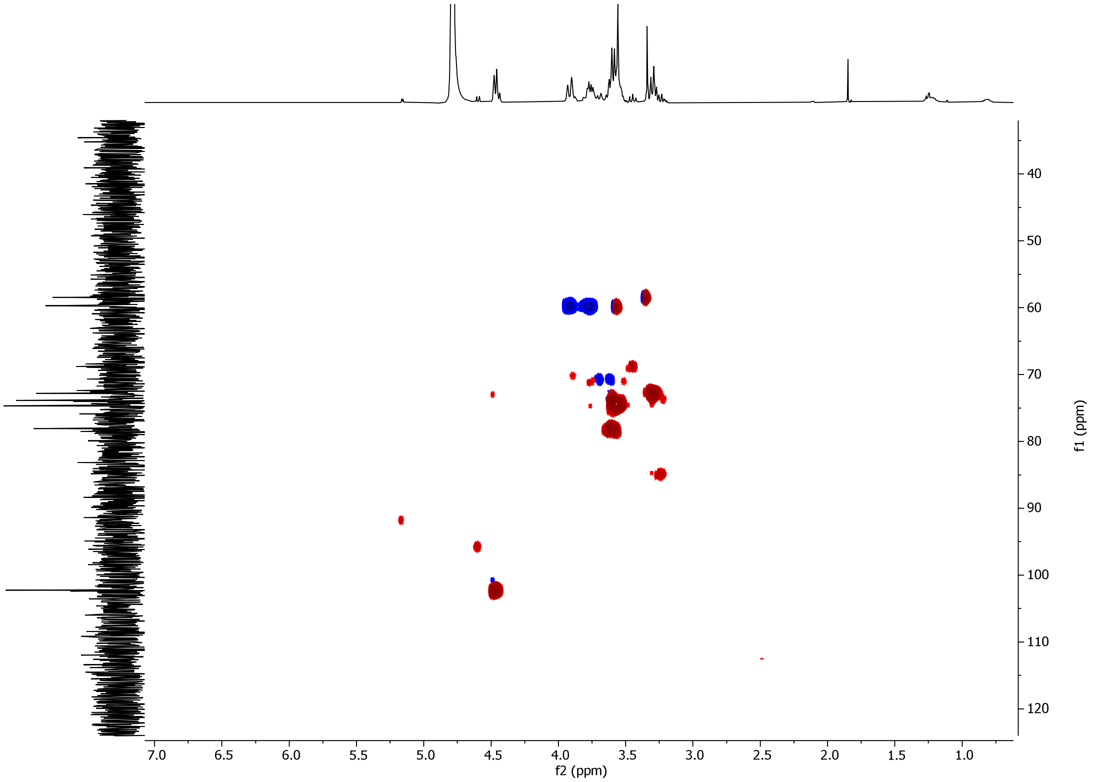

### 1.4.8 Synthesis of CA$_6$

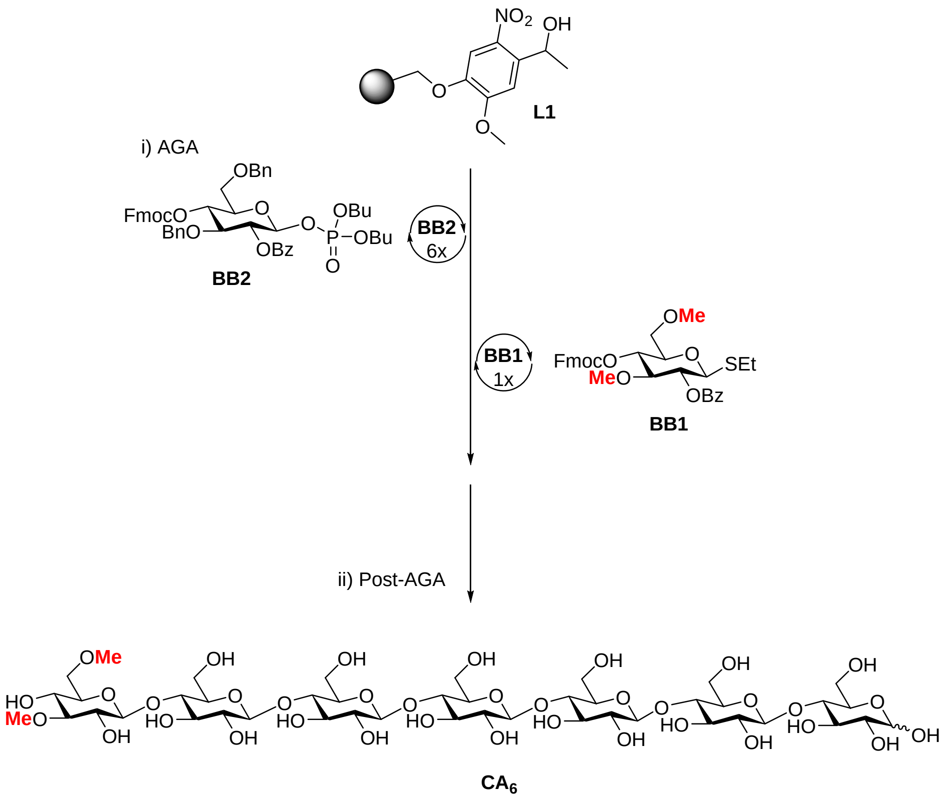


| Step | BB | Modules | Notes |
|---|---|---|---|
| AGA | | A | L1 swelling |
| | (BB1)x6 | (B, C1, D, E1)x6 | C2 (BB1, -30°C for 5 min, -10°C for 40 min) |
| | BB2 | B, C1, D, E1 | C1: (BB2, -20°C for 5 min, 0°C for 40 min) |
| Post-AGA | | F, G, H, I | F: (24 h) |
| | | | H: (16 h) |
| | | | I: C |

Automated synthesis, global deprotection, and purification afforded compound CA$_6$ as a white solid (4.5 mg, 31% overall yield).

Analytical data for CA$_6$:

$^1$H NMR (400 MHz, $D_2O$) δ 5.15 (d, $J$ = 3.8 Hz, 0.4 H, H1-α), 4.59 (d, $J$ = 7.9 Hz, 0.6 H, H1-β), 4.49 - 4.41 (m, 6H), 3.94 - 3.85 (m, 6H), 3.82 - 3.66 (m, 8H), 3.63 - 3.49 (m, 24H), 3.47 - 3.40 (m, 1H), 3.35 - 3.28 (m, 7H), 3.27 - 3.16 (m, 2H). $^{13}$C NMR (101 MHz, $D_2O$) δ 102.39, 102.25, 95.65, 91.72, 84.82, 78.52, 78.08, 74.72, 74.13, 73.86, 72.82, 71.18, 70.68, 70.01, 68.78, 59.70, 58.49, 48.73. (ESI-HRMS) m/z 1181.417 $[M + H]^+$ ($C_{44}H_{77}O_{36}$ requires 1181.420).

RP-HPLC of $CA_6$ (ELSD trace, Method A, $t_R$ = 16.8 min)

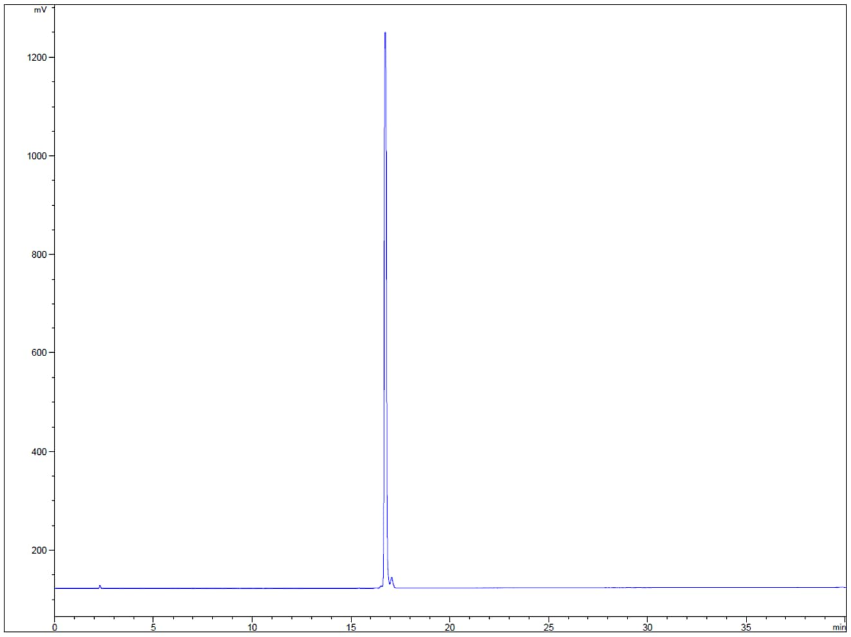

$^{1}$H NMR of $CA_6$ (400 MHz, $D_2O$)

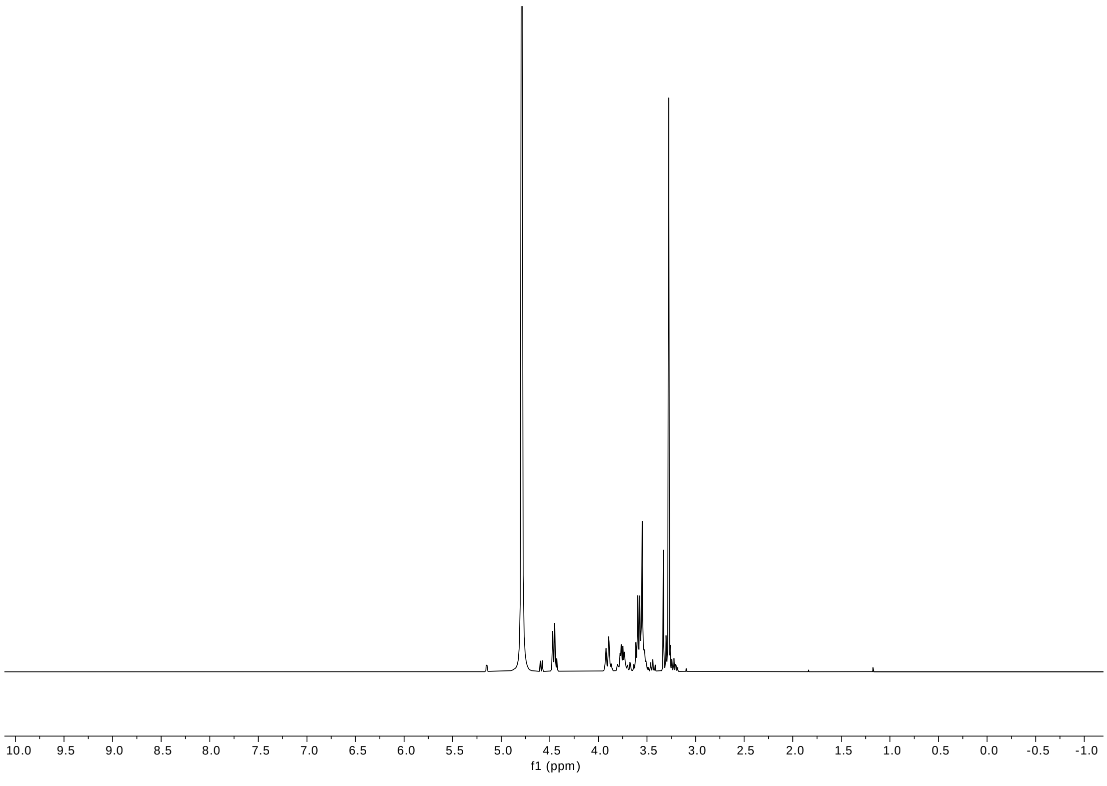

HSQC NMR of $CA_6$ ($D_2O$)

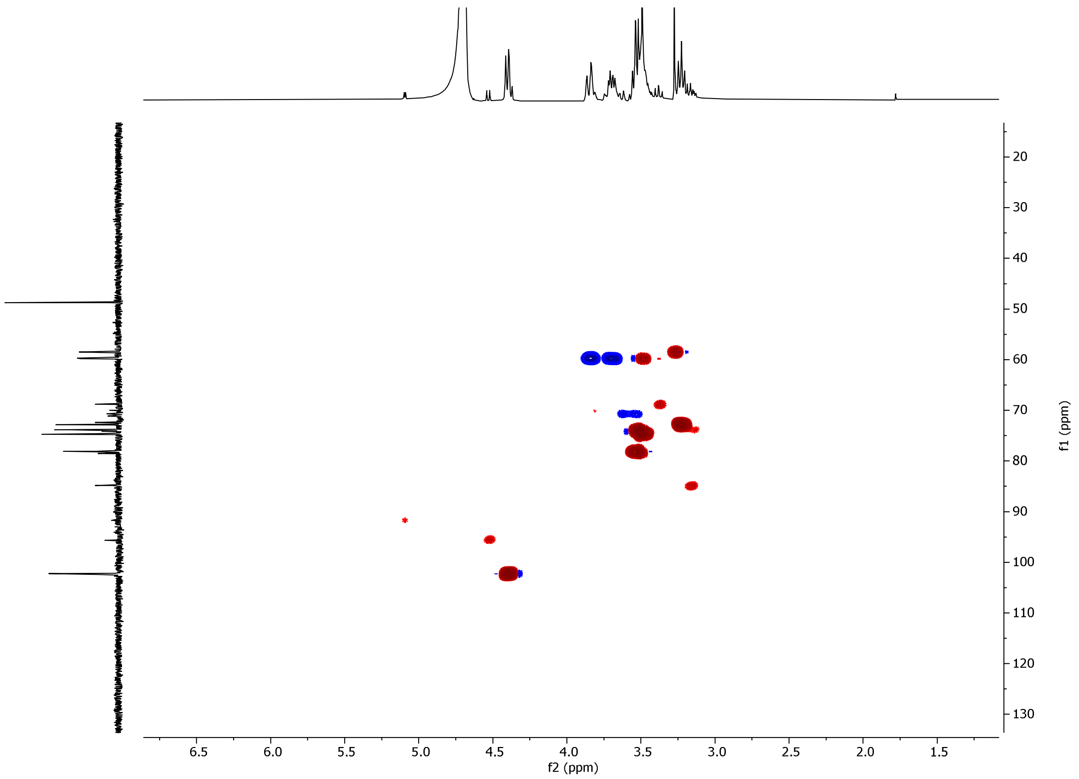

### 1.4.10 Synthesis of $A_7C$

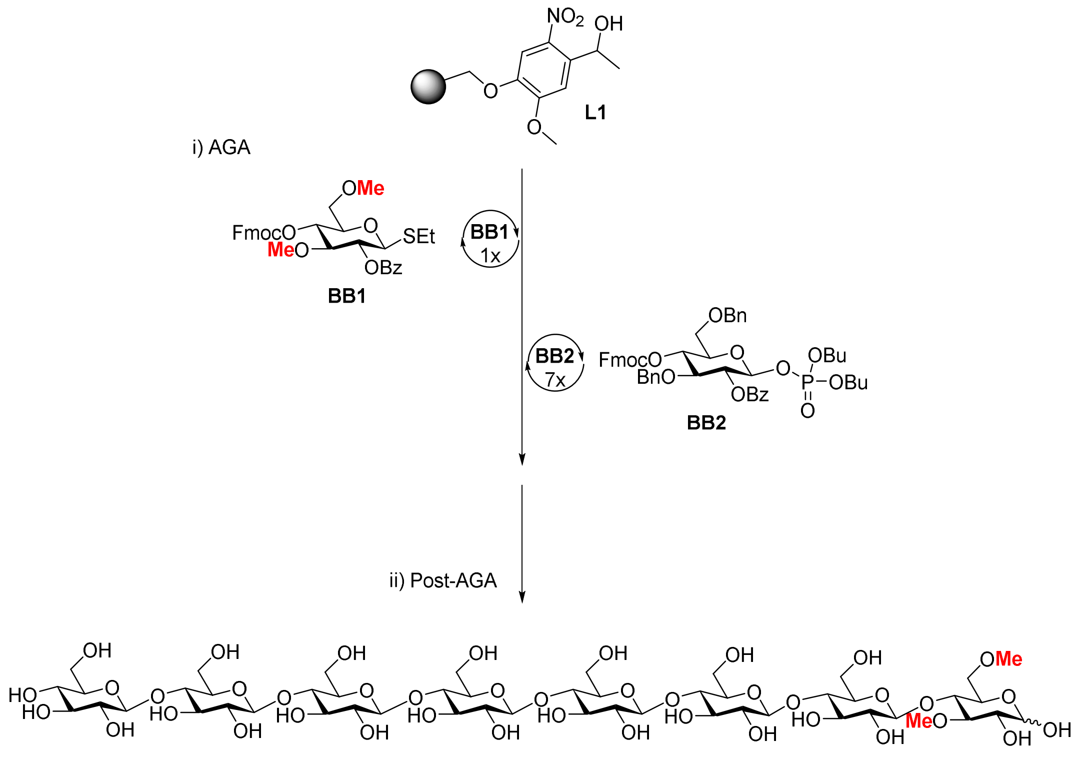


**$A_7C$**

| Step | BB | Modules | Notes |
|---|---|---|---|
| AGA | | A | L1 swelling |
| | BB2 | B, C1, D, E1 | C1: (BB2, -20°C for 5 min, 0°C for 40 min) |
| | (BB1)x7 | (B, C1, D, E1)x7 | C2 (BB1, -30°C for 5 min, -10°C for 40 min) |
| Post-AGA | | F, G, H, I | F: (24 h)<br>H: (16 h)<br>I: C |

Automated synthesis, global deprotection, and purification afforded compound $A_7C$ as a white solid (1.7 mg, 10% overall yield).

Analytical data for $A_7C$:

$^1$H NMR (600 MHz, $D_2O$) δ 5.18 (d, *J* = 3.5 Hz, 0.4H, H1-α), 4.63 (d, *J* = 7.9 Hz, 0.6H, H1-β), 4.51 (m, 6H), 4.42 (dd, *J* = 7.9, 5.1 Hz, 1H), 3.99 - 3.94 (m, 7H), 3.88 - 3.69 (m, 11H), 3.68 - 3.58 (m, 22H), 3.50 - 3.46 (m, 2H), 3.45 - 3.27 (m, 13H). $^{13}$C NMR (151 MHz, $D_2O$) δ 102.23, 102.23, 95.79, 91.76, 78.22, 75.64, 74.03, 72.91, 69.36, 60.50, 60.50, 59.85, 59.85, 59.85, 58.08. (ESI-HRMS) m/z 1343.475 $[M + H]^+$ ($C_{50}H_{87}O_{41}$ requires 1343.471).

RP-HPLC of $A_7C$ (ELSD trace, Method A, $t_R$ = 17.0 min)

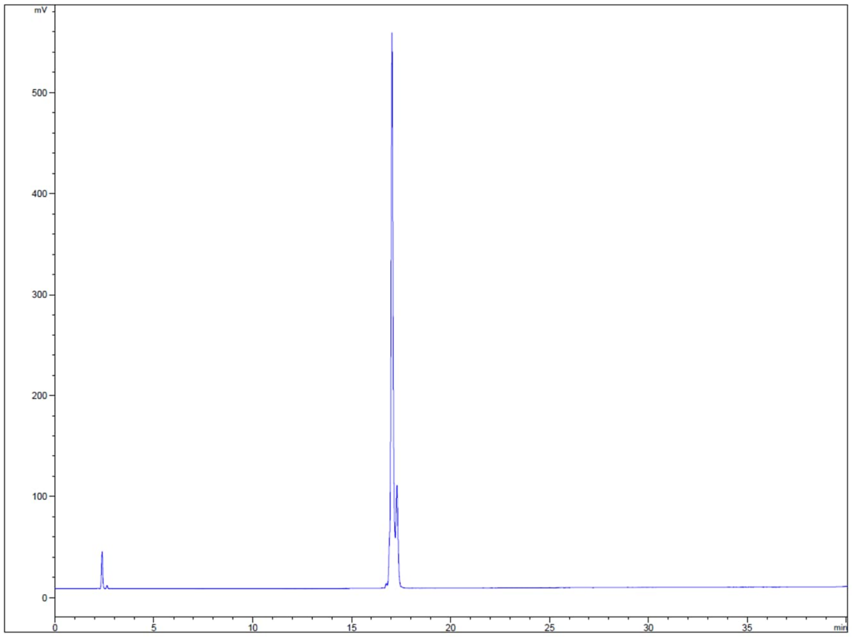


$^{1}$H NMR of $A_7C$ (600 MHz, $D_2O$)

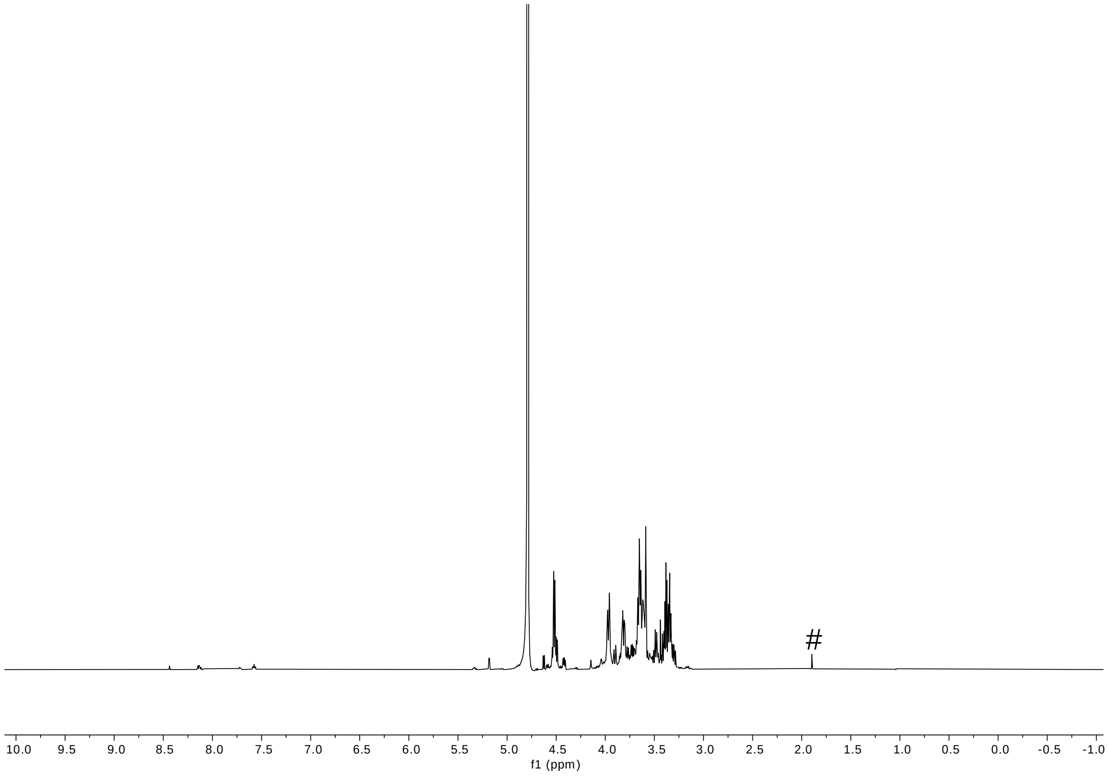


# Residual acetonitrile

HSQC NMR of $A_7C$ ($D_2O$)

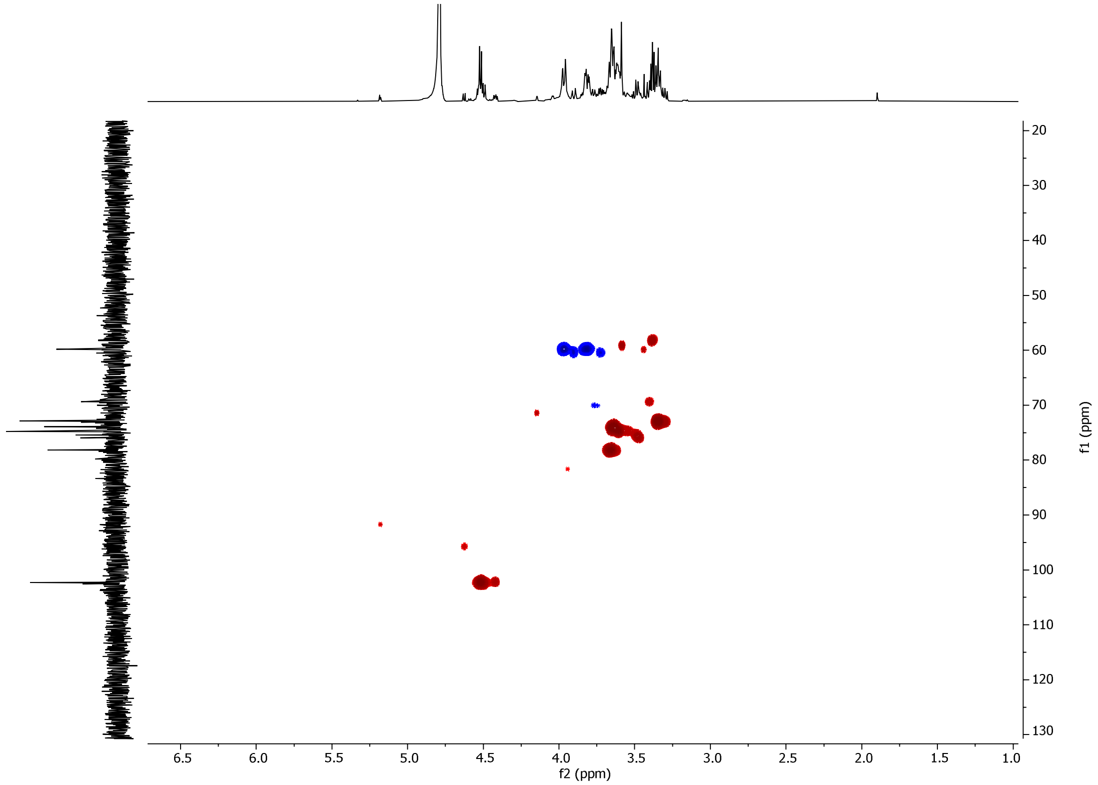

## 1.4.11 Synthesis of $A_6C$

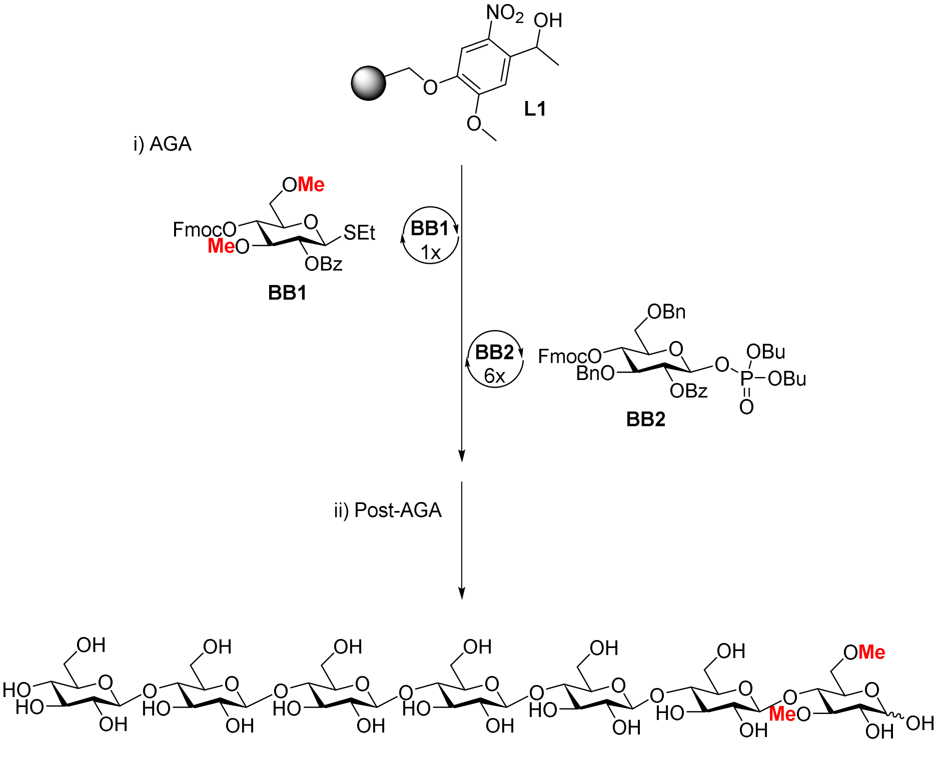


**$A_6C$**

| Step | BB | Modules | Notes |
|---|---|---|---|
| AGA | | A | L1 swelling |
| | BB2 | B, C1, D, E1 | C1: (BB2, -20°C for 5 min, 0°C for 40 min) |
| | (BB1)x6 | (B, C1, D, E1)x6 | C2 (BB1, -30°C for 5 min, -10°C for 40 min) |
| Post-AGA | | F, G, H, I | F: (24 h)<br>H: (16 h)<br>I: C |

Automated synthesis, global deprotection, and purification afforded compound $A_6C$ as a white solid (4.3 mg, 29% overall yield).

Analytical data for $A_6C$:

$^1$H NMR (600 MHz, $D_2O$) $\delta$ 5.20 (d, $J$ = 3.5 Hz, 0.4H, H1-$\alpha$), 4.65 (d, $J$ = 7.9 Hz, 0.6H H1-$\beta$), 4.57 - 4.50 (m, 5H), 4.44 (dd, $J$ = 7.9, 5.0 Hz, 1H), 4.06 - 3.96 (m, 5H), 3.92 (dd, $J$ = 12.4, 2.2 Hz, 1H), 3.89 - 3.71 (m, 9H), 3.71 - 3.59 (m, 20H), 3.58 - 3.47 (m, 3H), 3.47 - 3.29 (m, 10H). $^{13}$C NMR (151 MHz, $D_2O$) $\delta$ 102.54, 102.33, 95.84, 91.78, 83.36, 80.81, 78.35, 78.23, 75.96, 75.45, 74.80, 74.22, 73.97, 73.12, 72.91, 69.42, 60.54, 59.83, 59.46, 59.04, 58.25. (ESI-HRMS) m/z 1181.413 $[M + H]^+$ ($C_{44}H_{77}O_{36}$ requires 1181.419).

RP-HPLC of $A_6C$ (ELSD trace, Method A, $t_{R1}$ = 15.8 min, $t_{R2}$ = 16.1 min)
*Alpha/beta anomers at the free reducing end.

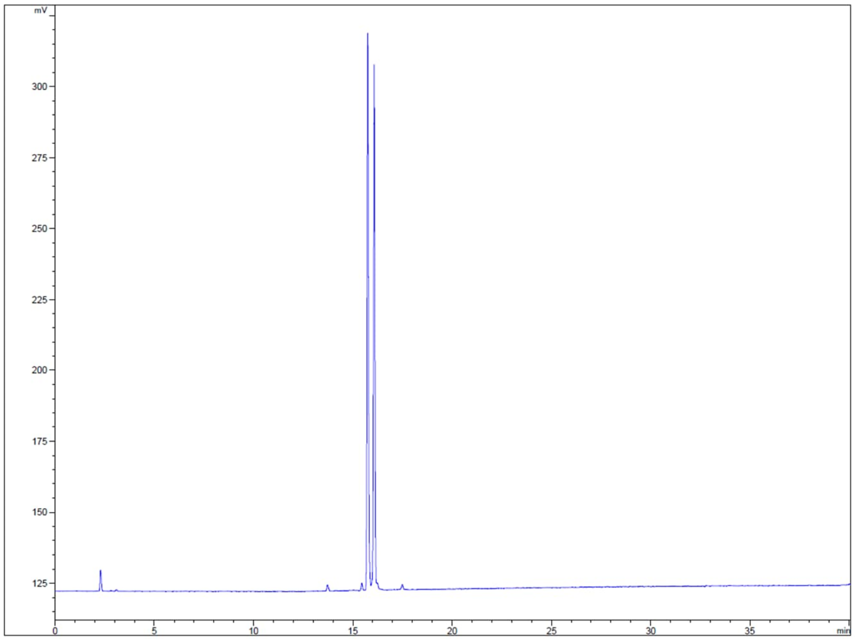


$^{1}$H NMR of $A_6C$ (400 MHz, $D_2O$)

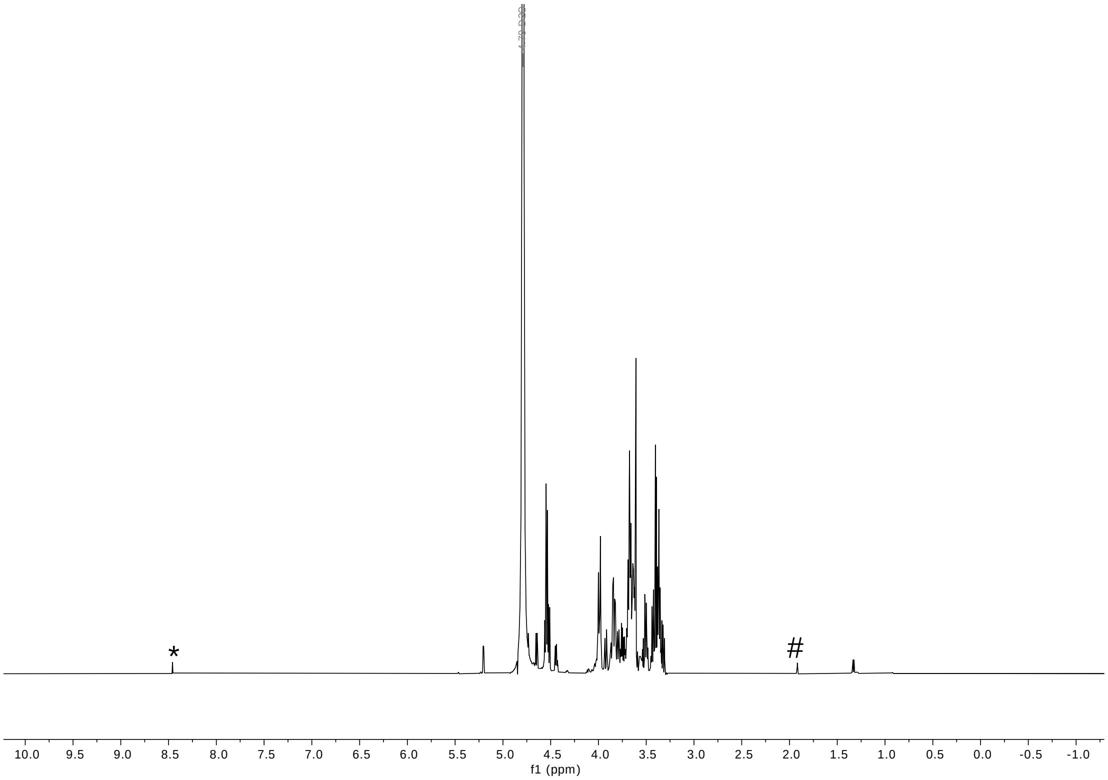


*Residual formic acid
# Residual acetonitrile

HSQC NMR of $A_6C$ ($D_2O$)

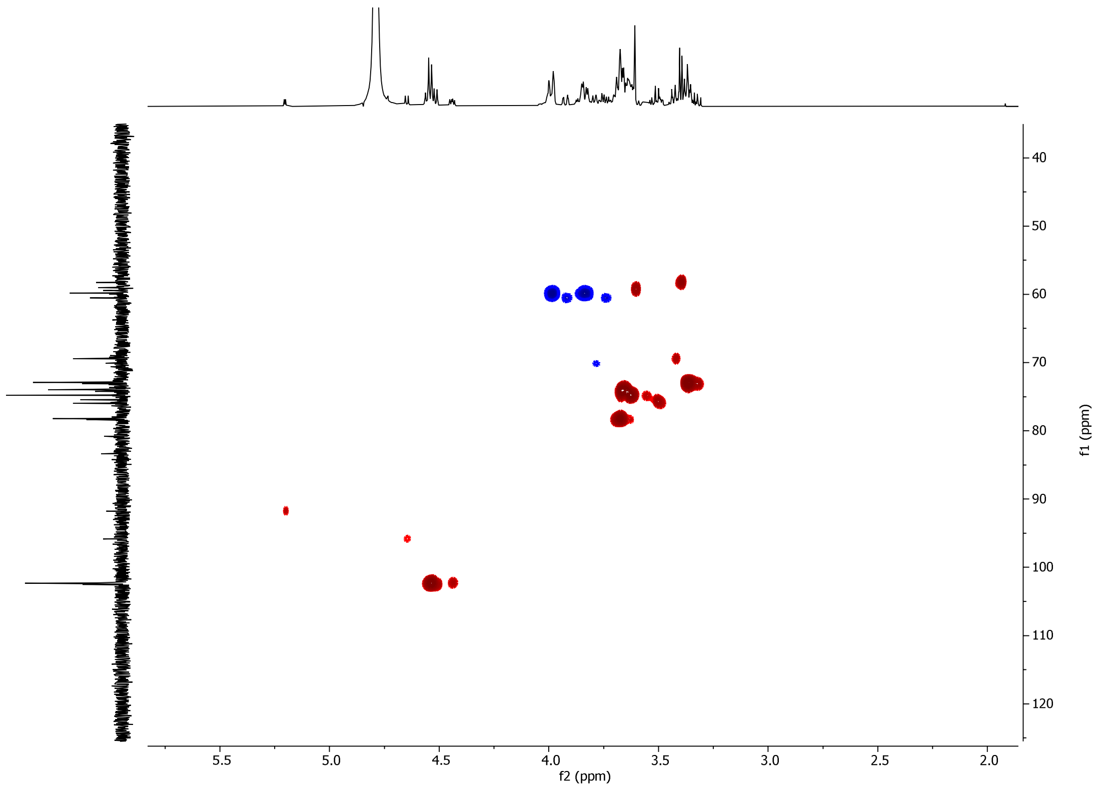

### 1.4.12 Synthesis of $MA_8C$

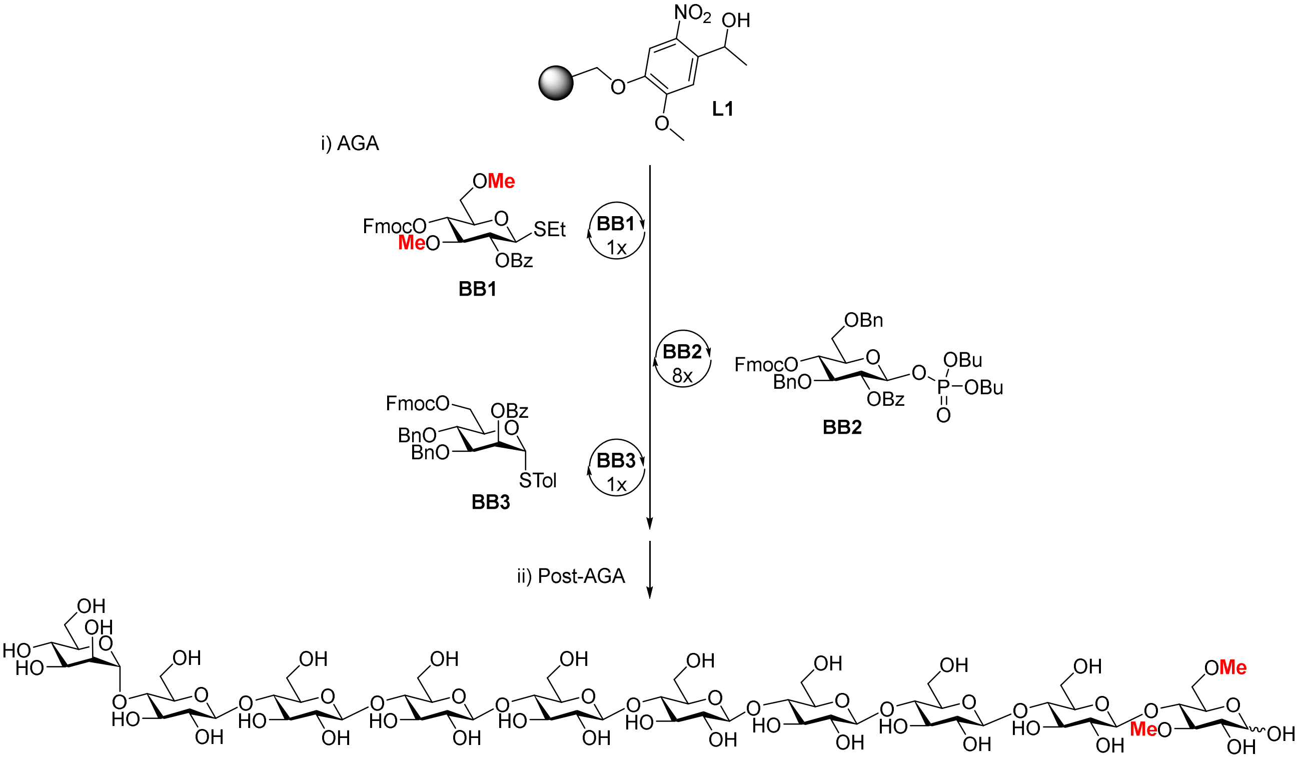


**$MA_8C$**

| Step | BB | Modules | Notes |
|---|---|---|---|
| AGA | | A | L1 swelling |
| | BB1 | B, C1, D, E1 | C1: (BB1, -20°C for 5 min, 0°C for 20 min) |
| | (BB2)x8 | (B, C1, D, E1)x8 | C2 (BB2, -30°C for 5 min, -10°C for 40 min) |
| | BB3 | B, C1, D, E1 | C1: (BB3, -20°C for 5 min, 0°C for 40 min) |
| Post-AGA | | F, G, H, I | F: (24 h)<br>H: (16 h)<br>I: C |

Automated synthesis, global deprotection, and purification afforded compound $MA_8C$ as a white solid (4.2 mg, 20% overall yield).

Analytical data for $MA_8C$:

$^1$H NMR (400 MHz, $D_2O$) δ 5.23 (d, $J$ = 1.8 Hz, 1H), 5.14 (d, $J$ = 3.2 Hz, 0.5H, H1-α), 4.59 (d, $J$ = 7.8 Hz, 0.5H, H1-β), 4.52 - 4.41 (m, 7H), 4.38 (dd, $J$ = 7.9, 3.6 Hz, 1H), 4.03 - 3.97 (m, 1H), 3.96 - 3.86 (m, 9H), 3.82 - 3.70 (m, 14H), 3.65 - 3.52 (m, 32H), 3.36 - 3.25 (m, 11H). $^{13}$C NMR (151 MHz, $D_2O$) δ = 102.38, 102.32, 101.31, 95.75, 91.96, 83.48, 78.21, 75.94, 74.79, 73.23, 72.90, 70.22, 66.50, 63.61, 60.63, 59.81, 58.24, (ESI-HRMS) m/z 1667.578 $[M + H]^+$ ($C_{62}H_{106}NaO_{51}$ requires 1667.577).

RP-HPLC of $MA_8C$ (ELSD trace, Method A, $t_{R1}$ = 16.7 min, $t_{R2}$ = 16.9 min)
*Alpha/beta anomers at the free reducing end.

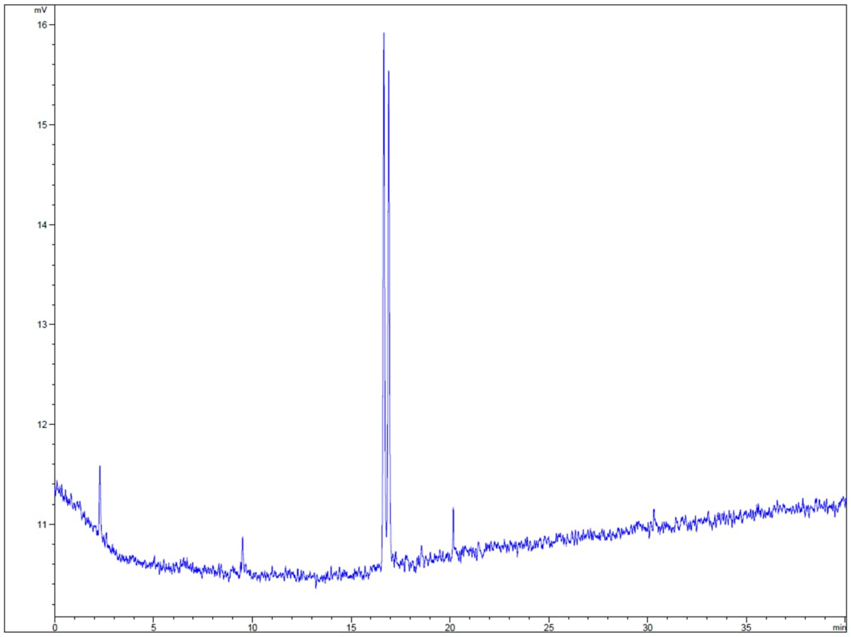


$^{1}$H NMR of $MA_8C$ (400 MHz, $D_2O$)

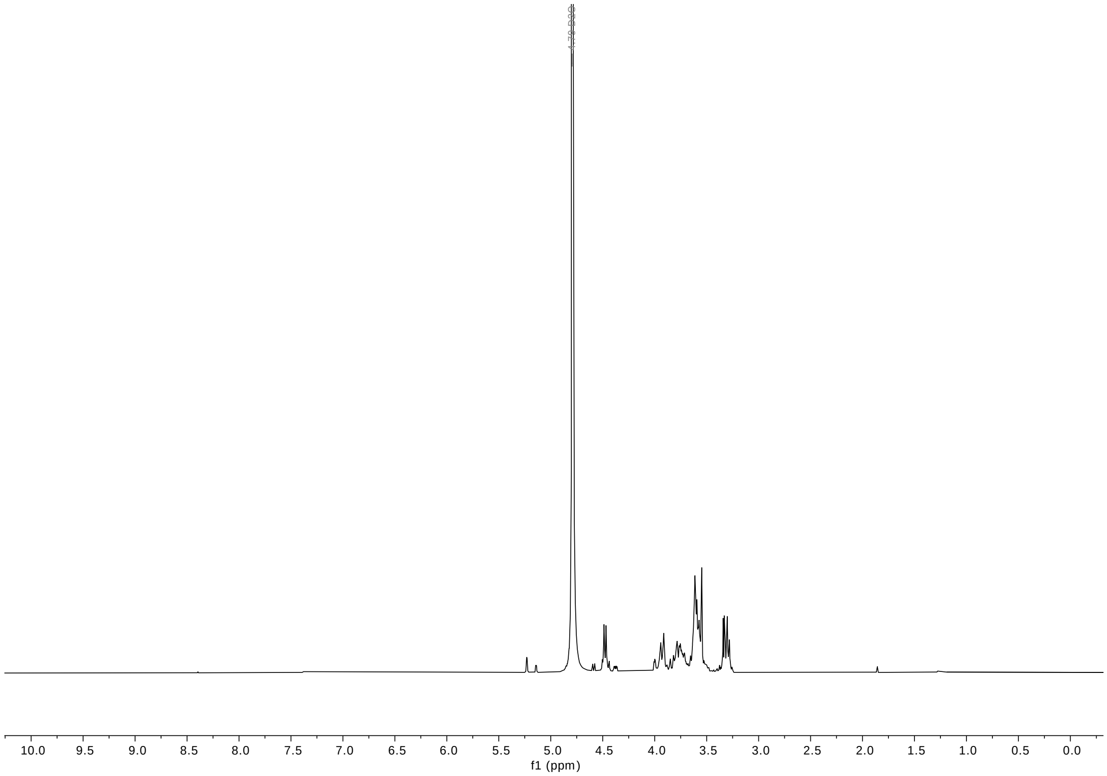

HSQC NMR of $MA_8C$ ($D_2O$)

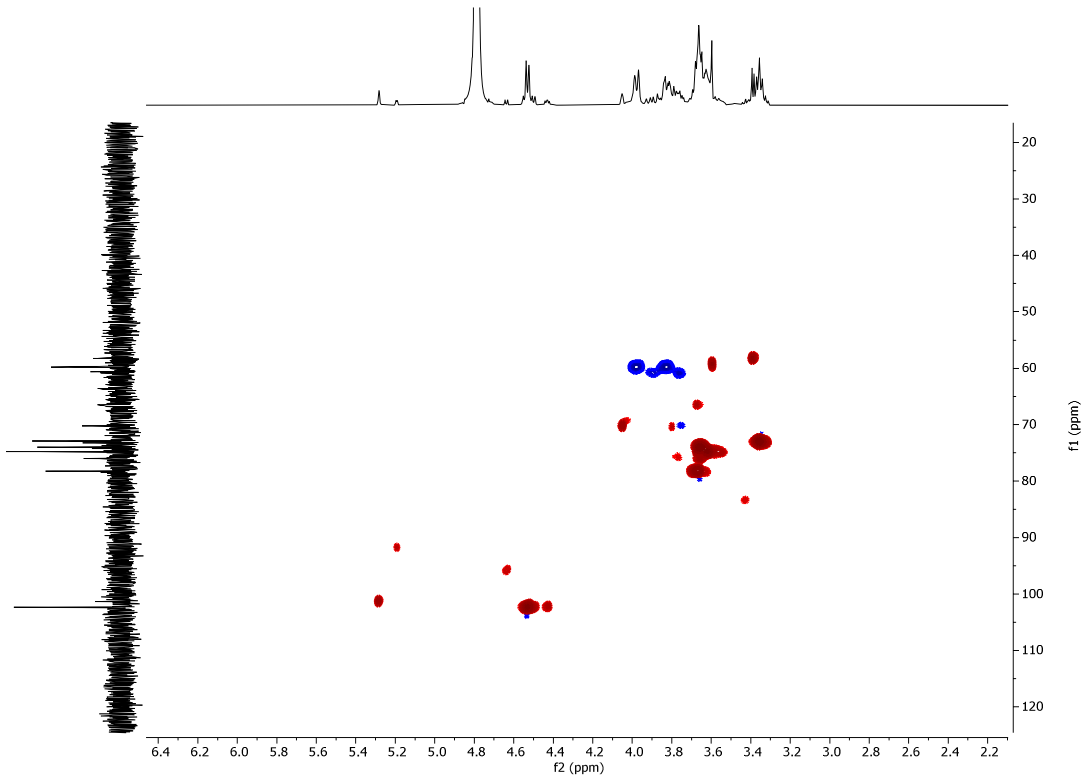

### 1.4.13 Synthesis of $GA_8C$

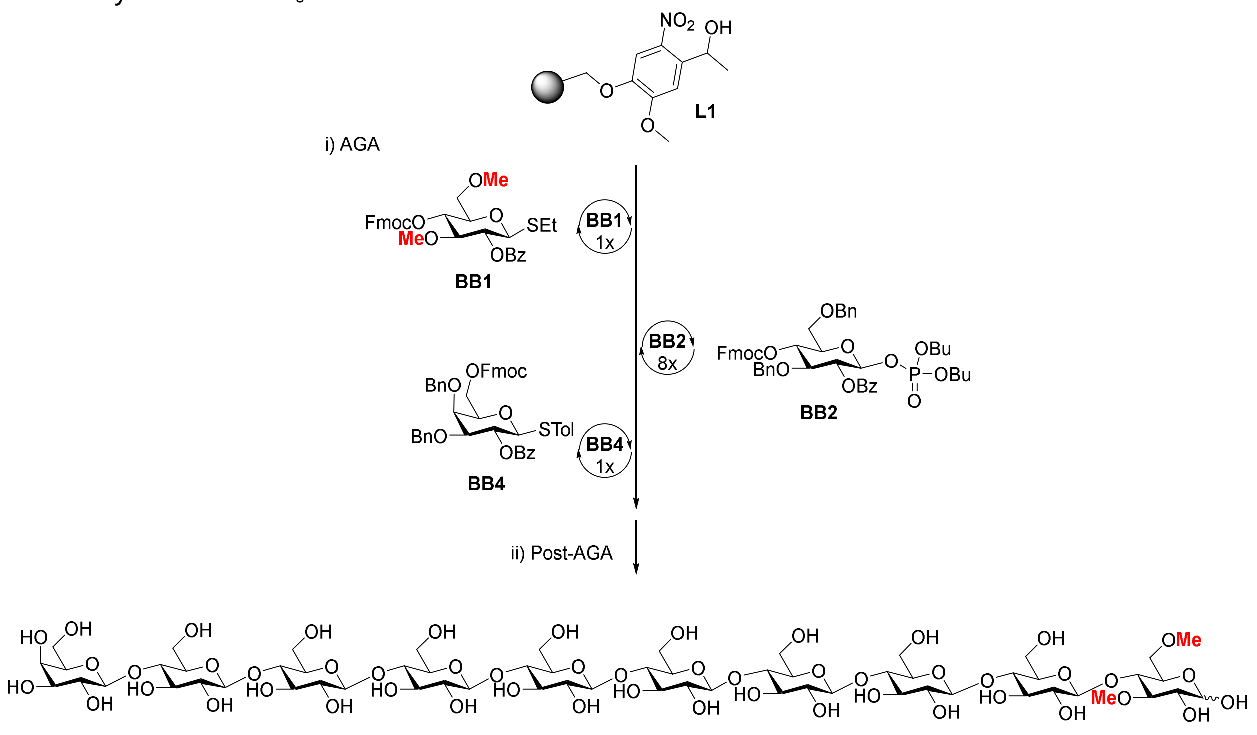


**$GA_8C$**

| Step | BB | Modules | Notes |
|---|---|---|---|
| AGA | | A | L1 swelling |
| | BB1 | B, C1, D, E1 | C1: (BB1, -20°C for 5 min, 0°C for 20 min) |
| | (BB2)x8 | (B, C1, D, E1)x8 | C2 (BB2, -30°C for 5 min, -10°C for 40 min) |
| | BB4 | B, C1, D, E1 | C1: (BB4, -20°C for 5 min, 0°C for 40 min) |
| Post-AGA | | F, G, H, I | F: (24 h)<br>H: (16 h)<br>I: C |

Automated synthesis, global deprotection, and purification afforded compound $GA_8C$ as a white solid (1.0 mg, 5% overall yield).

Analytical data for $GA_8C$:

$^1$H NMR (400 MHz, $D_2O$) δ 5.16 (d, $J$ = 3.2 Hz, 0.5H, H1-α), 4.61 (d, $J$ = 7.8 Hz, 0.5H, H1-β), 4.54 - 4.45 (m, 7H), 4.44 - 4.37 (m, 2H), 3.99 - 3.86 (m, 8H), 3.84 - 3.69 (m, 12H), 3.68 - 3.55 (m, 33H), 3.41 - 3.26 (m, 13H). $^{13}$C NMR (101 MHz, $D_2O$) δ 102.55, 102.39, 95.94, 91.91, 78.20, 75.94, 74.98, 74.17, 73.04, 68.69, 59.98, 59.82, 59.34, 58.37. (ESI-HRMS) m/z 1689.577 $[M + Na]^+$ ($C_{62}H_{106}NaO_{51}$ requires 1689.560).

RP-HPLC of $GA_8C$ (ELSD trace, Method A, $t_R$ = 18.2 min)

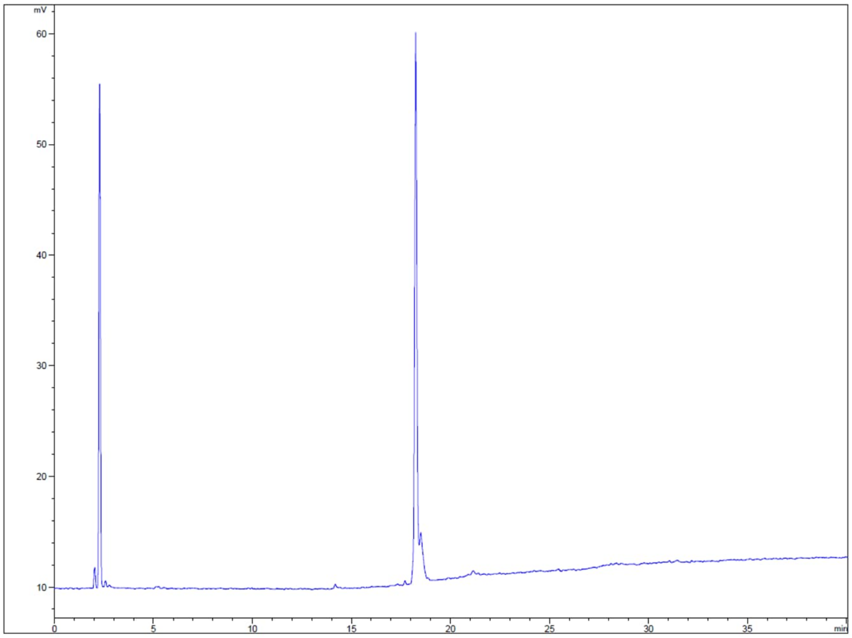


$^{1}$H NMR of $GA_8C$ (400 MHz, $D_2O$)

*                                                                 #

*Residual formic acid

# Residual acetonitrile

HSQC NMR of $GA_8C$ ($D_2O$)

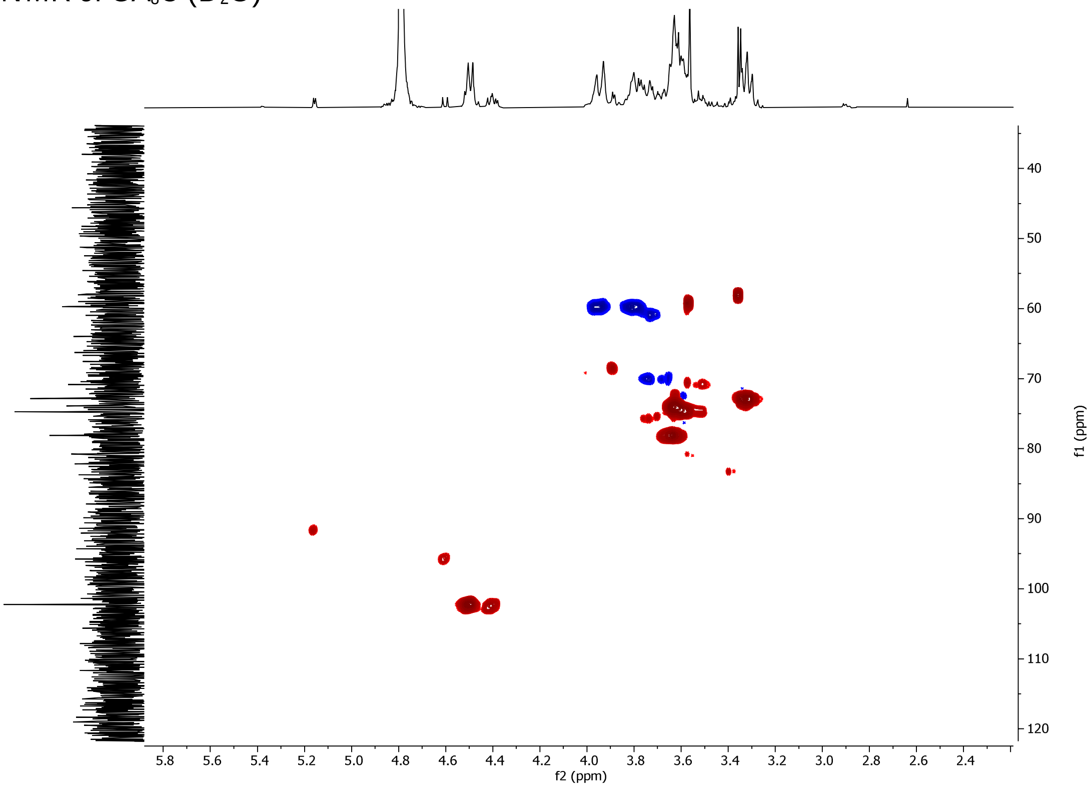

### 1.4.14 Synthesis of NA$_8$C

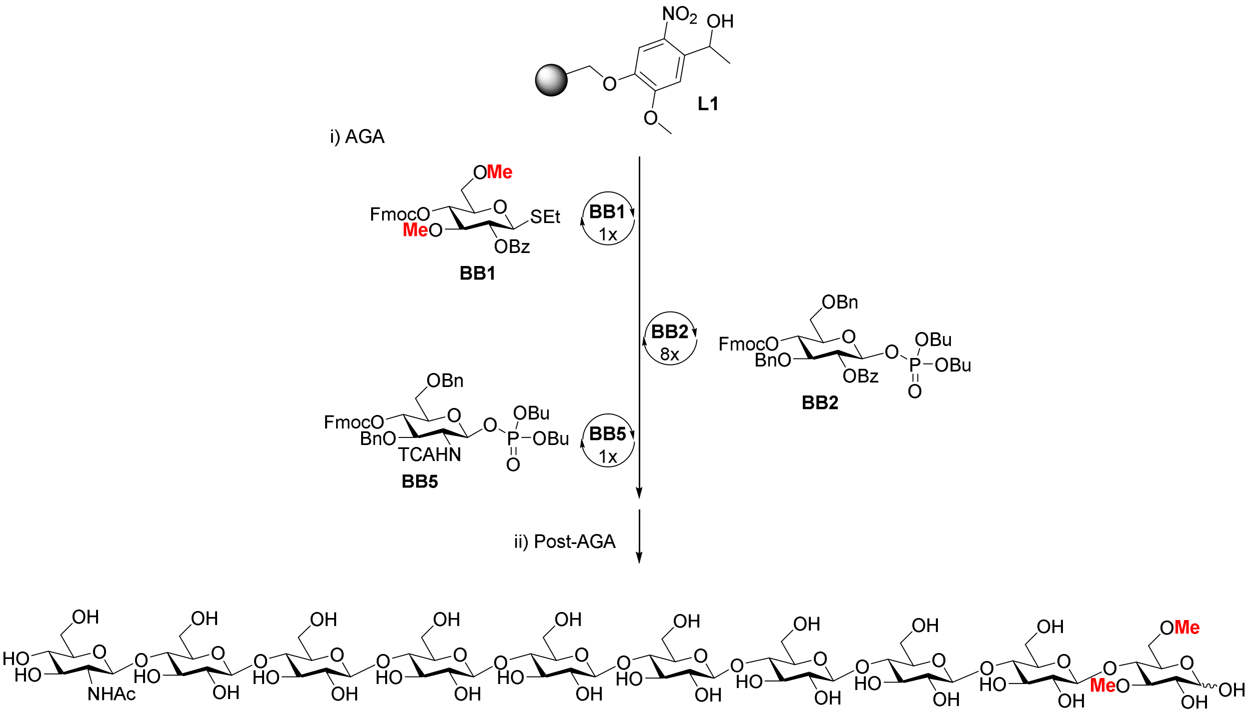


**NA$_8$C**

| Step | BB | Modules | Notes |
|---|---|---|---|
| AGA | | A | L1 swelling |
| | BB1 | B, C1, D, E1 | C1: (BB1, -20°C for 5 min, 0°C for 20 min) |
| | (BB2)x8 | (B, C1, D, E1)x8 | C2 (BB2, -30°C for 5 min, -10°C for 40 min) |
| | BB5 | B, C1, D, E1 | C1: (BB5, -35°C for 5 min, -15°C for 40 min) |
| Post-AGA | | F, G, H, I | F: (24 h)<br>H: (16 h)<br>I: C |

Automated synthesis, global deprotection, and purification afforded compound NA$_8$C as a white solid (3.1 mg, 15% overall yield).

Analytical data for NA$_8$C:

$^1$H NMR (400 MHz, $D_2O$) δ 5.13 (d, $J$ = 3.2 Hz, 0.4 H, H1-α), 4.58 (d, $J$ = 7.8 Hz, 0.6 H, H1-β), 4.52 - 4.43 (m, 9H), 4.37 (dd, $J$ = 8.0, 3.6 Hz, 1H), 3.96 - 3.87 (m, 8H), 3.81 - 3.69 (m, 12H), 3.64 - 3.52 (m, 32H), 3.41 (d, J = 7.2 Hz, 2H), 3.34 - 3.26 (m, 12H), 2.00 (s, 3H). $^{13}$C NMR (101 MHz, $D_2O$) δ 102.27, 95.80, 91.77, 78.10, 74.72, 73.87, 72.82, 59.71, 21.95. (ESI-HRMS) m/z 1708.604 $[M + H]^+$ ($C_{64}H_{110}NO_{51}$ requires 1708.604).

RP-HPLC of $NA_8C$ (ELSD trace, Method A, $t_{R1}$ = 16.7 min, $t_{R2}$ = 17.0 min)
*Alpha/beta anomers at the free reducing end.

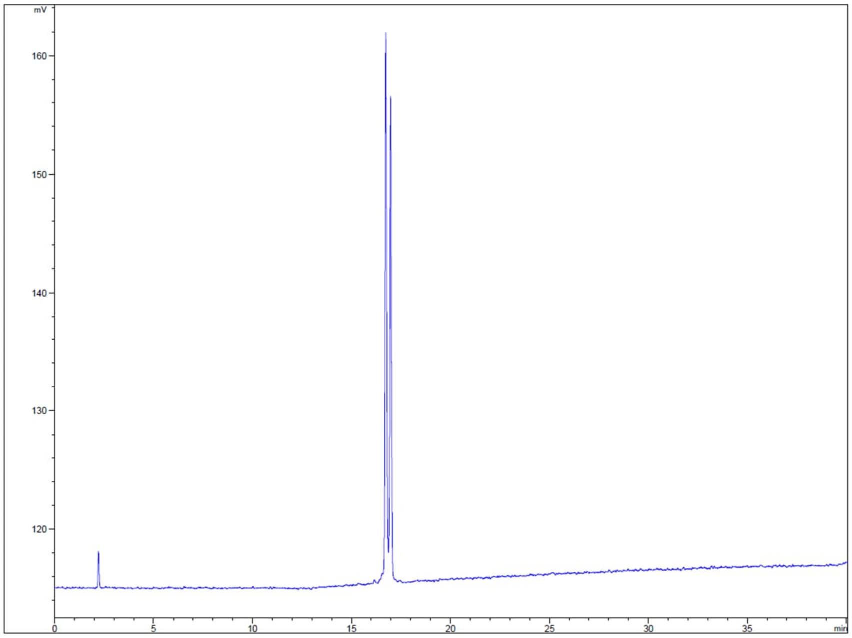


$^{1}$H NMR of $NA_8C$ (400 MHz, $D_2O$)

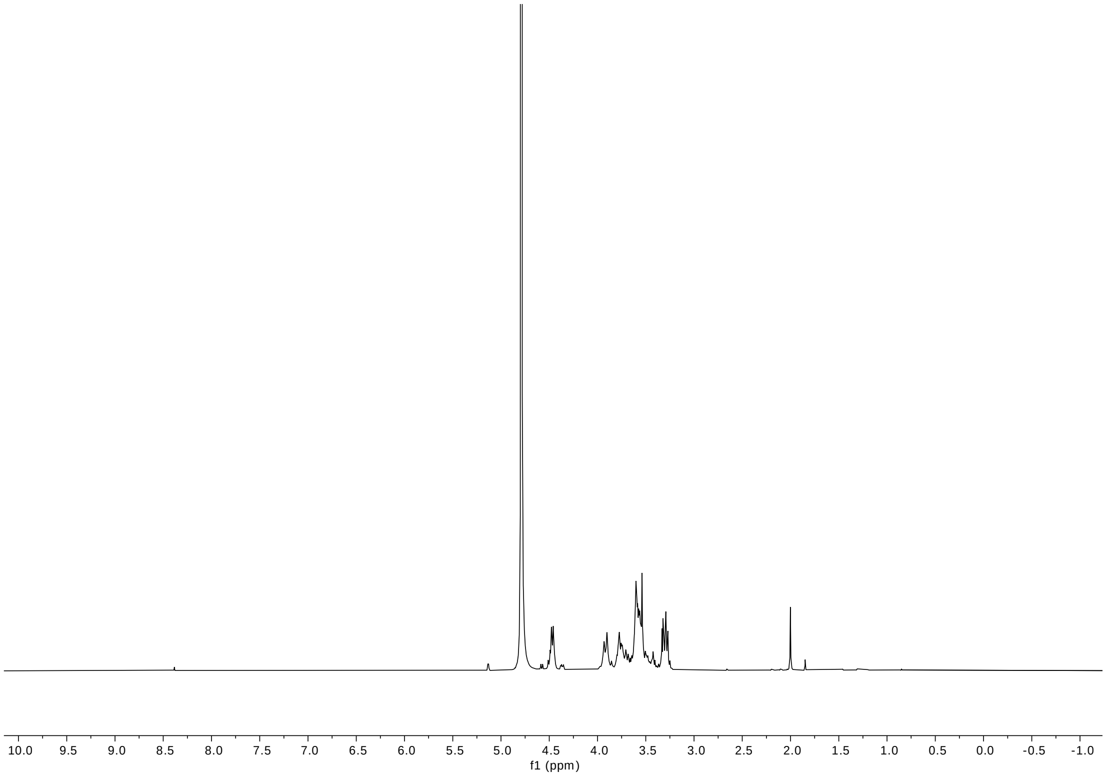

HSQC NMR of $NA_8C$ ($D_2O$)

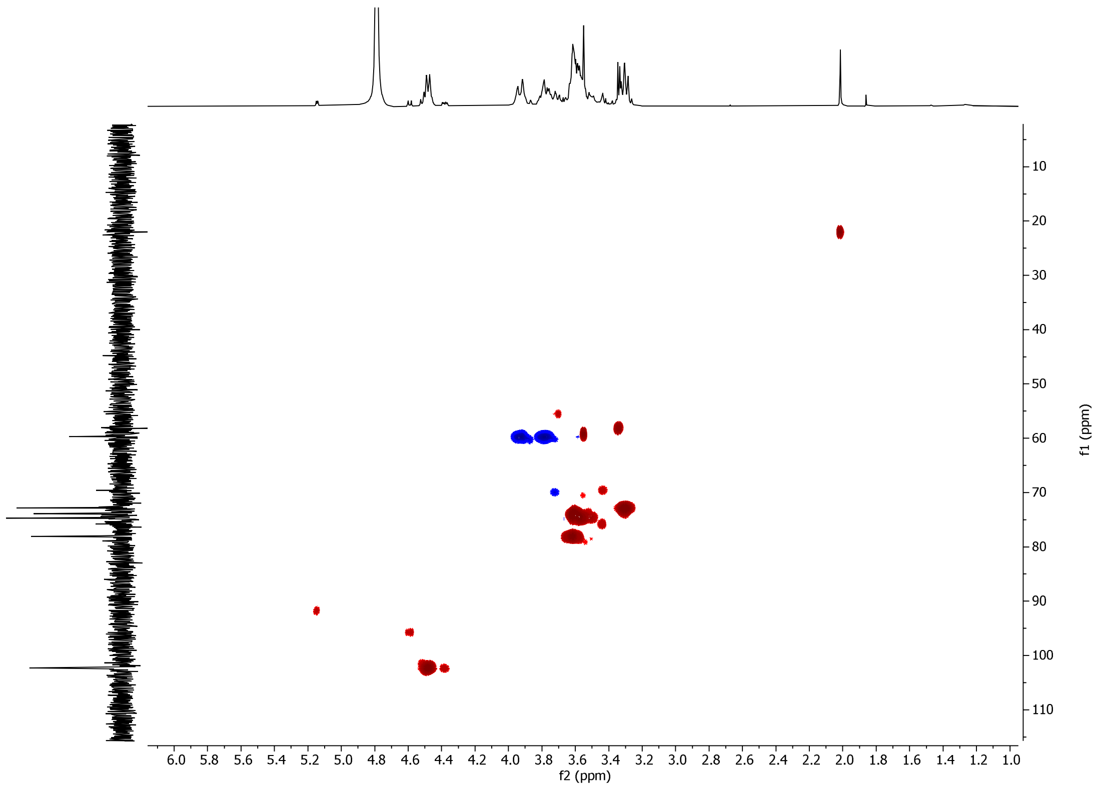

### 1.4.15 Synthesis of $QA_8C$

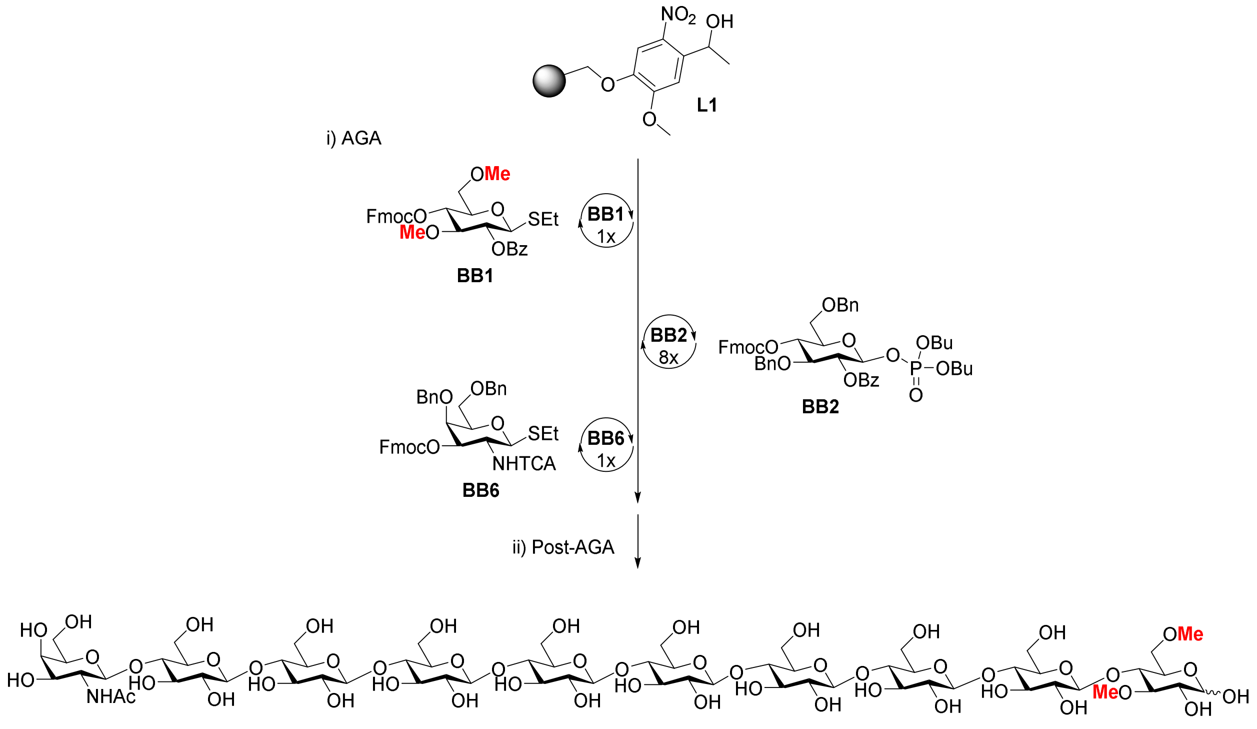


QA8C

| Step | BB | Modules | Notes |
|---|---|---|---|
| AGA | | A | L1 swelling |
| | BB1 | B, C1, D, E1 | C1: (BB1, -20°C for 5 min, 0°C for 20 min) |
| | (BB2)x8 | (B, C1, D, E1)x8 | C2 (BB2, -30°C for 5 min, -10°C for 40 min) |
| | BB6 | B, C1, D, E1 | C1: (BB6, -20°C for 30 min, 0°C for 50 min) |
| Post-AGA | | F, F1, G, H, I | F: (24 h)<br>H: (16 h)<br>I: C |

Automated synthesis, global deprotection, and purification afforded compound $QA_8C$ as a white solid (3.1 mg, 15% overall yield).

Analytical data for $QA_8C$:

$^1H$ NMR (600 MHz, $D_2O$) δ 5.15 (d, $J$ = 3.5 Hz, 0.5H, H1-α), 4.59 (d, $J$ = 7.9 Hz, 0.5H, H1-β), 4.52 - 4.43 (m, 8H), 4.38 (dd, J = 7.9, 5.4 Hz, 1H), 3.96 - 3.91 (m, 7H), 3.91 - 3.86 (m, 2H), 3.82 - 3.75 (m, 9H), 3.75 - 3.67 (m, 6H), 3.65 - 3.55 (m, 29H), 3.54 - 3.48 (m, 1H), 3.38 - 3.26 (m, 12H), 2.02 (s, 3H). $^{13}C$ NMR (HSQC, 100 MHz, $D_2O$) δ 102.39, 102.23, 95.95, 91.76, 78.22, 74.36, 73.07, 67.59, 59.85, 59.85, 59.21, 58.24, 22.14. (ESI-HRMS) m/z 1708.605 $[M + H]^+$ ($C_{64}H_{110}NO_{51}$ requires 1708.604).

RP-HPLC of $QA_8C$ (ELSD trace, Method A, $t_{R1}$ = 17.1 min, $t_{R2}$ = 17.3 min)
*Alpha/beta anomers at the free reducing end.

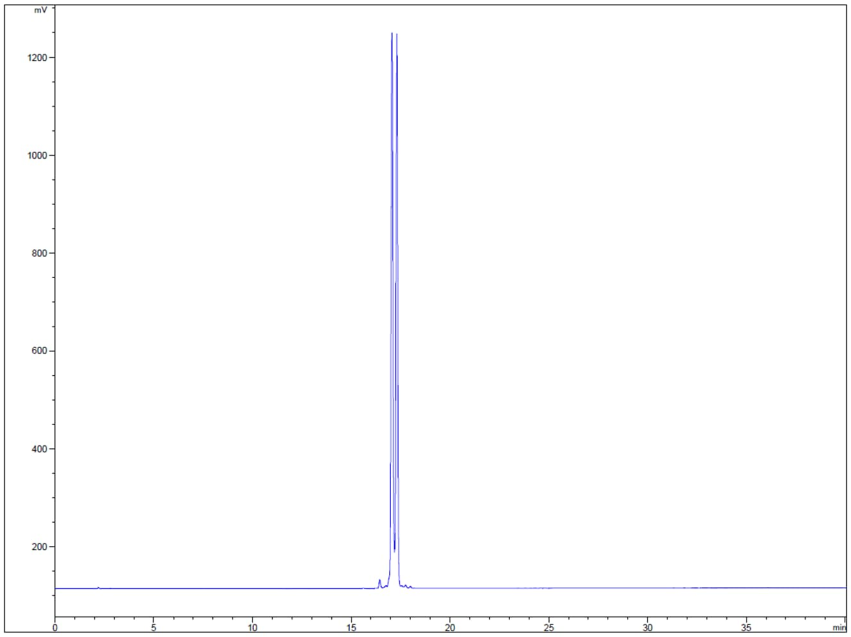


$^1H$ NMR of $QA_8C$ (400 MHz, $D_2O$)

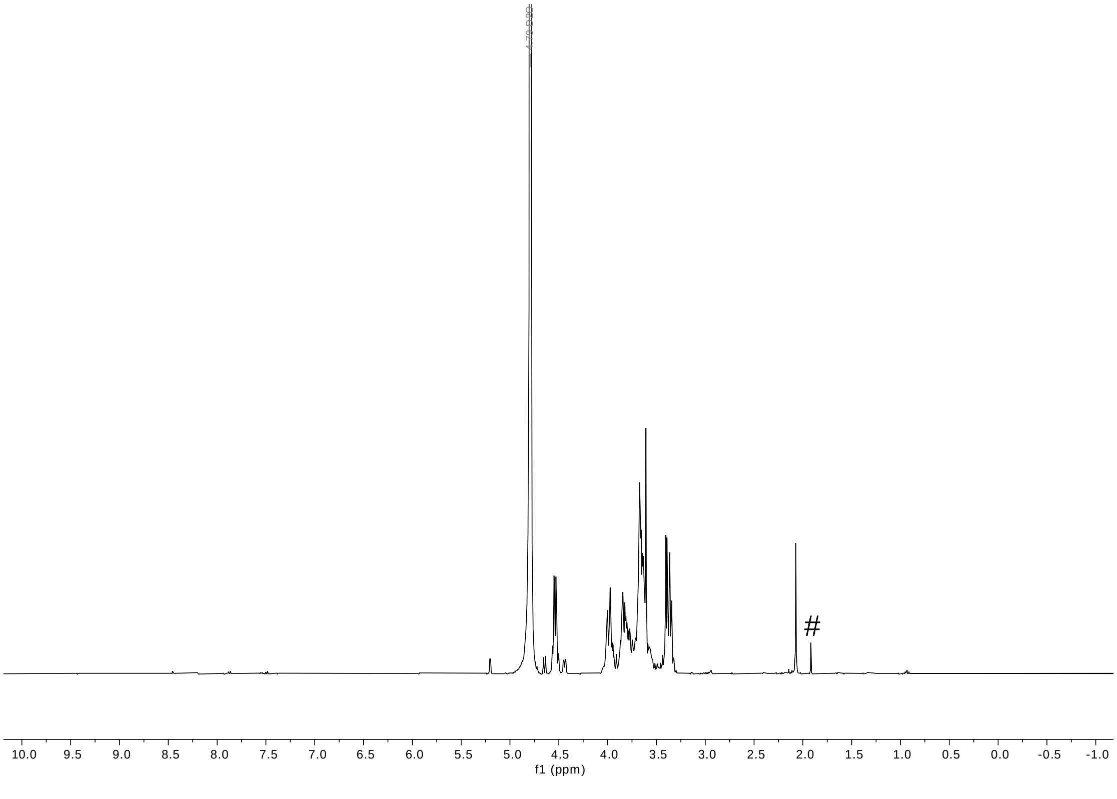


# Residual acetonitrile

HSQC NMR of $QA_8C$ ($D_2O$)

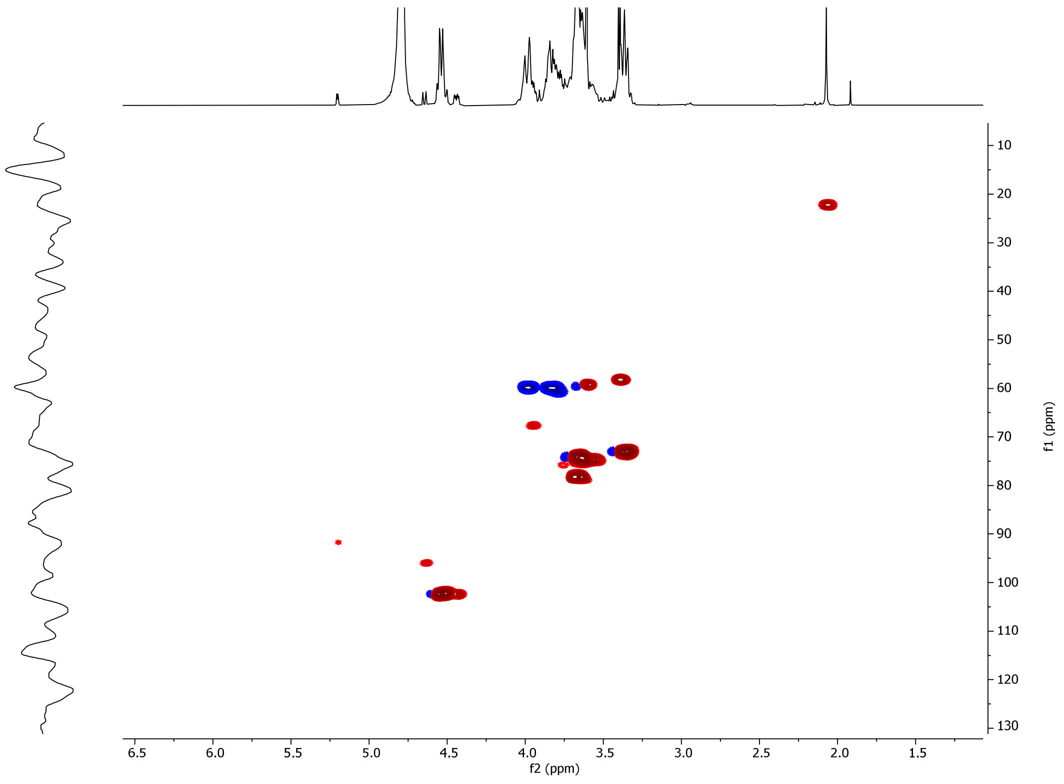

### 1.4.16 Synthesis of $EA_8C$

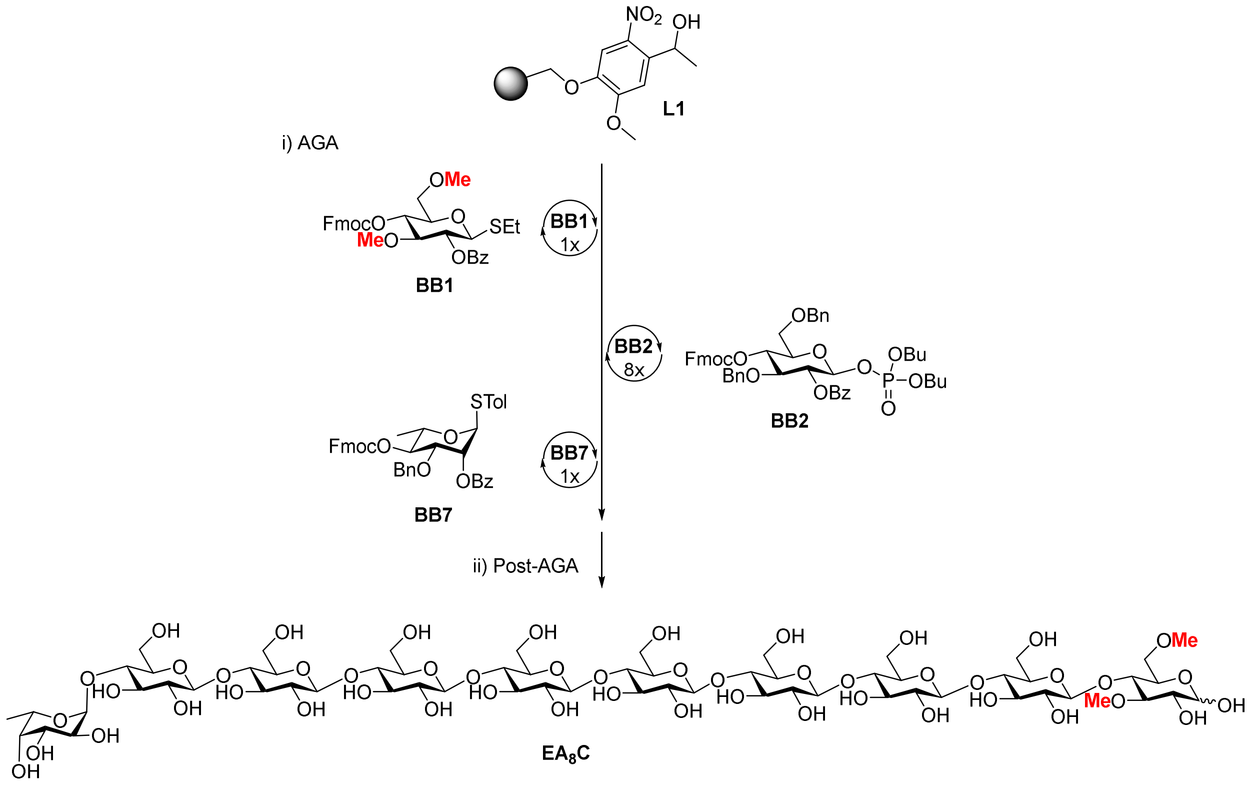


| Step | BB | Modules | Notes |
|---|---|---|---|
| AGA | | A | L1 swelling |
| | BB1 | B, C1, D, E1 | C1: (BB1, -20°C for 5 min, 0°C for 20 min) |
| | (BB2)x8 | (B, C1, D, E1)x8 | C2 (BB2, -30°C for 5 min, -10°C for 40 min) |
| | BB7 | B, C1, D, E1 | C1: (BB7, -40°C for 5 min, -20°C for 20 min) |
| Post-AGA | | F, G, H, I | F: (24 h)<br>H: (16 h)<br>I: C |

Automated synthesis, global deprotection, and purification afforded compound $EA_8C$ as a white solid (7.6 mg, 37% overall yield).

Analytical data for $EA_8C$:

$^1H$ NMR (600 MHz, $D_2O$) δ 5.15 (d, $J$ = 3.5 Hz, 0.4H, H1-α), 4.89 (d, $J$ = 4.1 Hz, 1H), 4.59 (d, $J$ = 7.9 Hz, 0.6H, H1-β), 4.51 - 4.45 (m, 7H), 4.38 (dd, $J$ = 7.9, 5.4 Hz, 1H), 4.33 (dd, $J$ = 6.8, 6.7 Hz, 1H), 3.93 (d, J = 11.6 Hz, 8H), 3.82 - 3.76 (m, 10H), 3.75 - 3.70 (m, 3H), 3.66 - 3.54 (m, 28H), 3.53 - 3.48 (m, 3H), 3.35 - 3.28 (m, 11H), 1.13 (d, J = 6.5 Hz, 3H). $^{13}C$ NMR (151 MHz, $D_2O$) δ 102.27, 99.45, 95.79, 91.60, 83.38, 78.12, 76.67, 75.23, 74.73, 73.89, 73.46, 72.84, 69.29, 67.97, 66.90, 59.73, 59.44, 58.18, 15.14. (ESI-HRMS) m/z 1651.584 $[M + H]^+$ ($C_{62}H_{107}O_{50}$ requires 1651.582).

RP-HPLC of $EA_8C$ (ELSD trace, Method A, $t_{R1}$ = 17.0 min, $t_{R2}$ = 17.2 min)
*Alpha/beta anomers at the free reducing end.

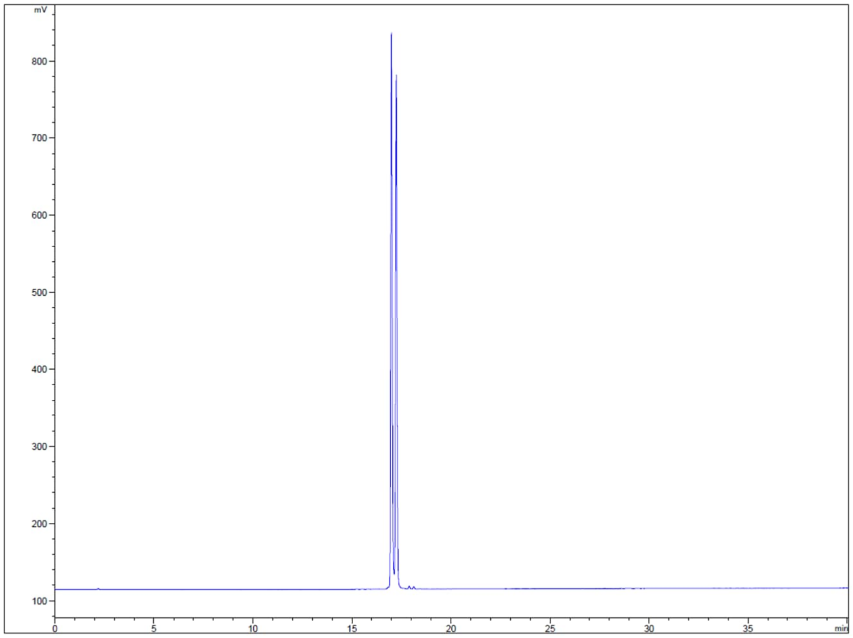


$^1H$ NMR of $EA_8C$ (400 MHz, $D_2O$)

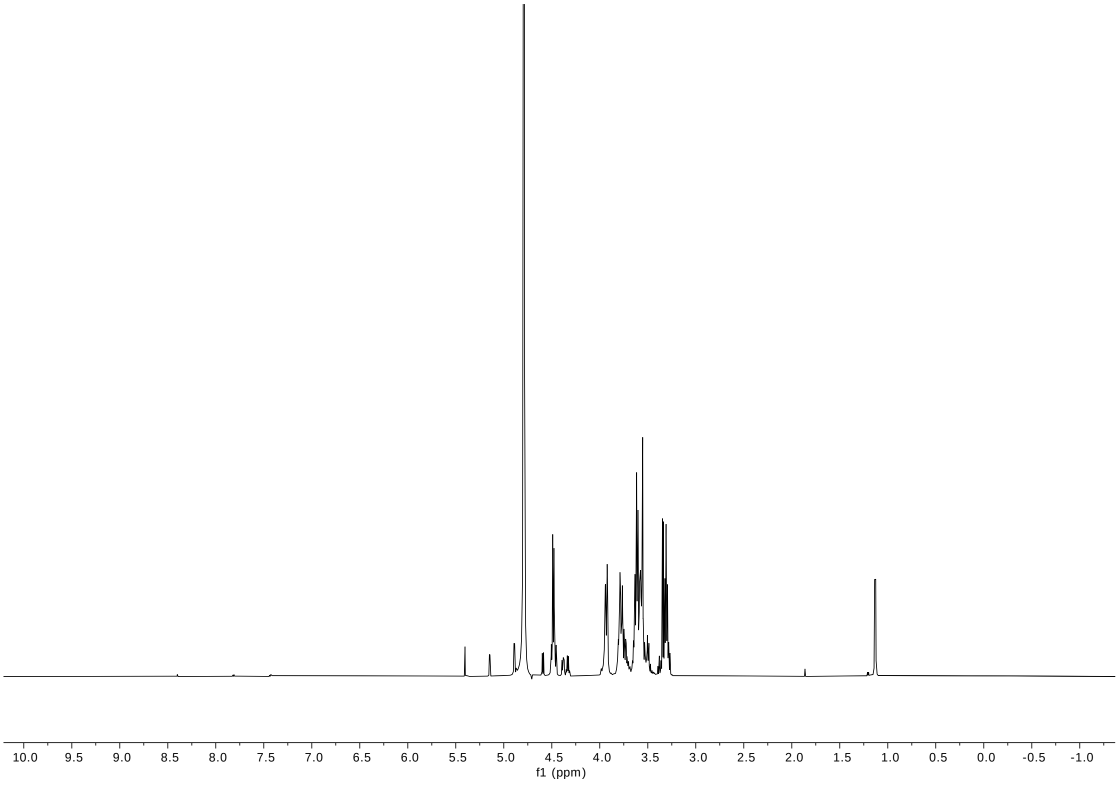

HSQC NMR of $EA_8C$ ($D_2O$)

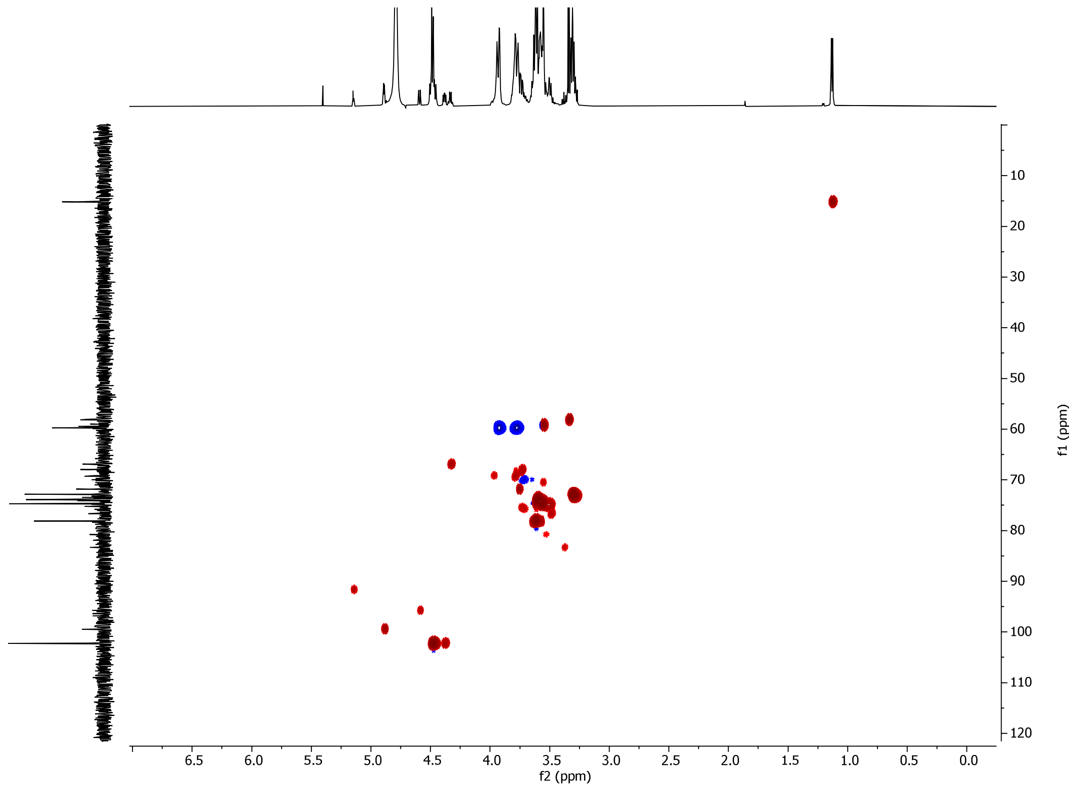

### 1.4.17 Synthesis of $RA_8C$

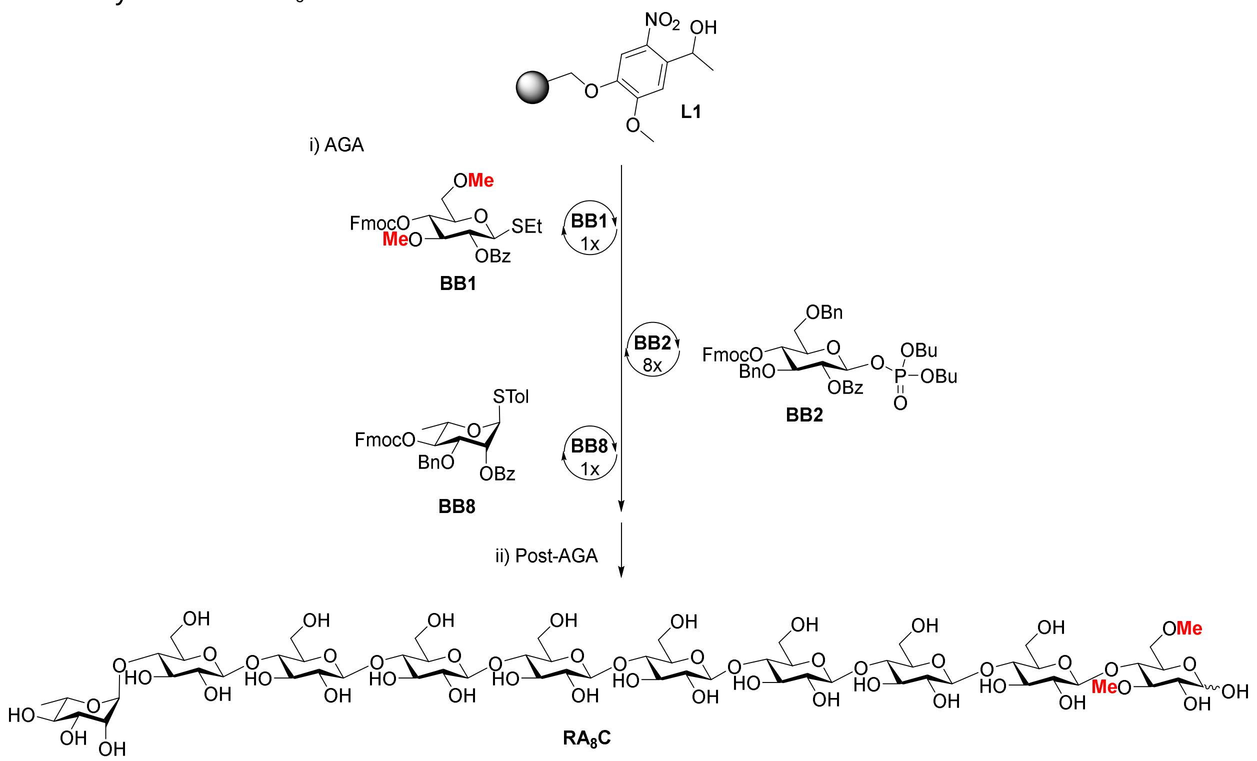


**$RA_8C$**

| Step | BB | Modules | Notes |
|---|---|---|---|
| AGA | | A | L1 swelling |
| | BB1 | B, C1, D, E1 | C1: (BB1, -20°C for 5 min, 0°C for 20 min) |
| | (BB2)x8 | (B, C1, D, E1)x8 | C2 (BB2, -30°C for 5 min, -10°C for 40 min) |
| | BB8 | B, C1, D, E1 | C1: (BB8, -20°C for 5 min, 0°C for 40 min) |
| Post-AGA | | F, G, H, I | F: (24 h)<br>H: (16 h)<br>I: C |

Automated synthesis, global deprotection, and purification afforded compound $RA_8C$ as a white solid (6.2 mg, 30% overall yield).

Analytical data for $RA_8C$:

$^1$H NMR (600 MHz, $D_2O$) δ 5.20 (d, *J* = 3.5 Hz, 0.4H, H1-α), 4.86 (d, *J* = 1.8 Hz, 1H), 4.65 (d, *J* = 7.9 Hz, 1H, H1-β), 4.57 - 4.50 (m, 7H), 4.44 (dd, *J* = 7.9, 5.1 Hz, 1H), 4.04 - 3.96 (m, 9H), 3.91 - 3.81 (m, 8H), 3.80 - 3.71 (m, 5H), 3.70 - 3.60 (m, 27H), 3.60 - 3.54 (m, 2H), 3.48 - 3.42 (m, 1H), 3.41 - 3.31 (m, 12H), 1.27 (d, J = 6.3 Hz, 3H). $^{13}$C NMR (151 MHz, $D_2O$) δ 102.42, 102.33, 100.89, 95.83, 91.78, 83.35, 80.82, 78.23, 77.07, 75.88, 75.48, 75.08, 74.80, 74.22, 74.13, 73.97, 73.68, 73.48, 73.23, 73.05, 72.91, 71.86, 70.61, 70.33, 70.12, 69.09, 60.06, 59.83, 59.46, 59.04, 58.26, 58.14, 16.42. (ESI-HRMS) *m/z* 1651.585 $[M+H]^+$ ($C_{62}H_{107}O_{50}$ requires 1651.582).

RP-HPLC of $RA_8C$ (ELSD trace, Method A, $t_{R1}$ = 17.5 min, $t_{R2}$ = 17.7 min)
*Alpha/beta anomers at the free reducing end.

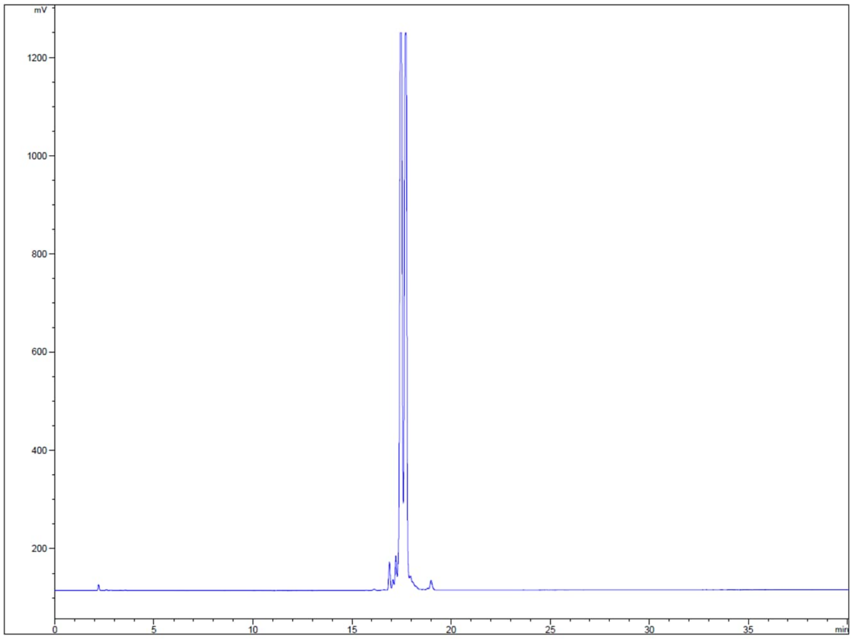


$^1H$ NMR of $RA_8C$ (400 MHz, $D_2O$)

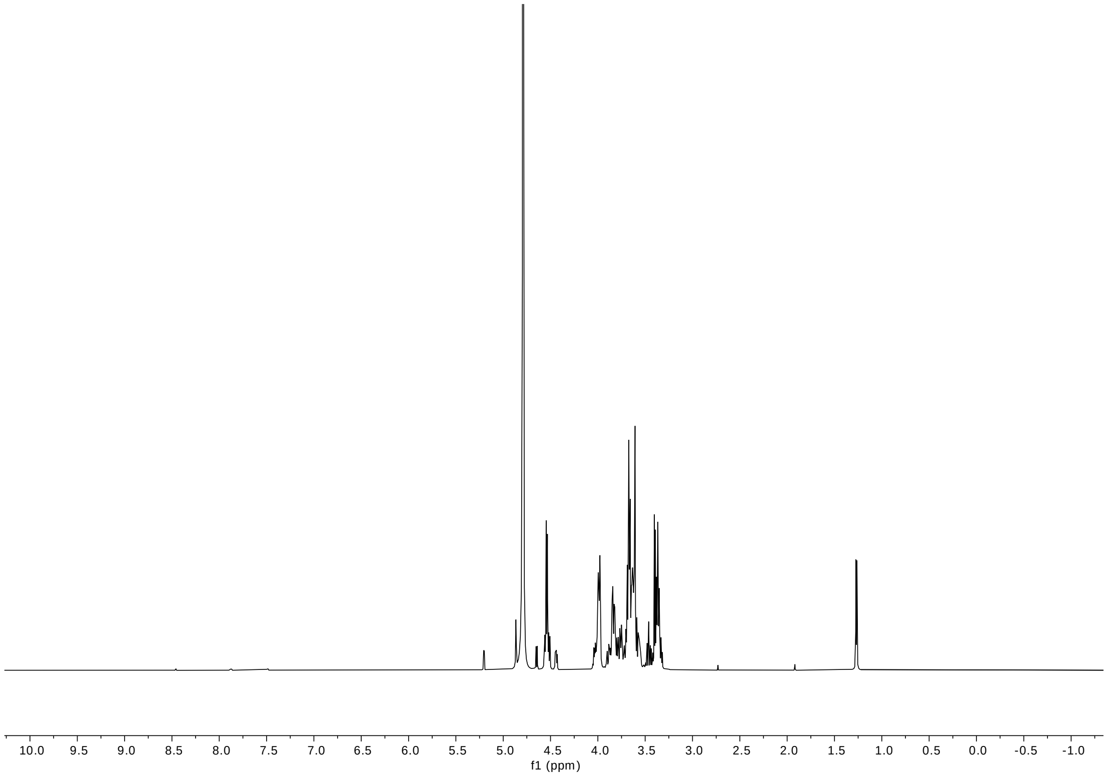

HSQC NMR of $RA_8C$ ($D_2O$)

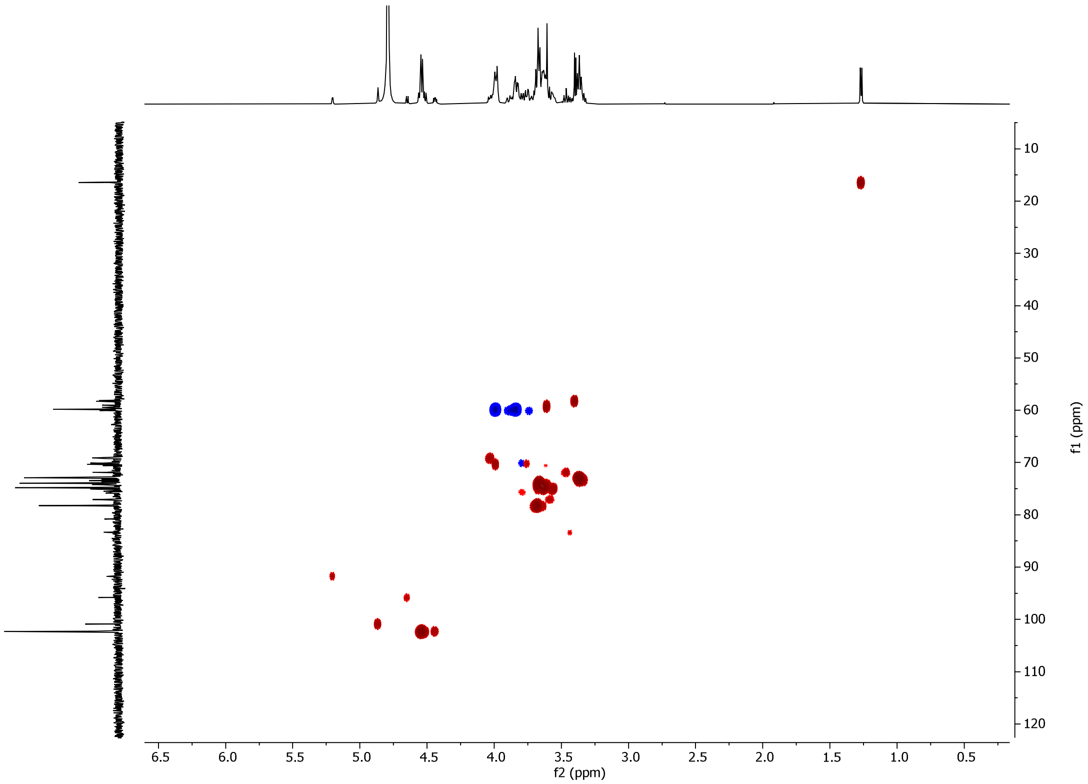

# 2 Self-assembly and hydrogel formation

## 2.1 General methods

### 2.1.1 XRD

X-ray diffraction experiments were carried out using a SmartLab high-resolution X-ray diffractometer (Rigaku Corporation, Japan). The scans were performed in the scattering angle range between 5°and 45° with a step of 0.05°and an accumulation time of 6 or 10 s. Raw XRD profiles were corrected by subtraction of the sample holder signal, smoothing, and baseline correction. The oligosaccharide samples were lyophilized prior to XRD measurement.

### 2.1.2 TEM imaging

Transmission electron microscopy was conducted using a JEOL JEM F200 (Jeol, Japan) (S)TEM equipped with a field emission gun and a TVIPS TemCam-F216 (2k x 2k) camera. The microscope was operated at 80 kV; a condenser aperture with a diameter of 200 μm was used. For specimen preparation, 2.5 μL of suspension (final conc 0.1% (w/w)) were deposited on glow-discharged carbon-coated copper grids (Plano GmbH, Germany).

### 2.1.3 Cryo-TEM

Cryo-transmission electron microscopy (cryo-TEM) of $GA_6$ and $GA_8C$

A droplet (*ca.* 3 μL) of the suspension was deposited on a glow-discharged lacey carbon-coated grid. Blotting and plunge freezing of the grid in liquid ethane were carried out using an automatic plunge freezer (EM-GP, Leica Microsystems, Germany). Transmission electron microscopy was performed using a JEM-2100Plus (JEOL Ltd., Japan) operated at an accelerating voltage at 200 kV. An Elsa cryo-transfer holder was used for the observations under cryogenic conditions. All images were recorded on a Gatan Rio 16 camera (Gatan Inc., USA) using SerialEM[14] and ED patterns were recorded on a MerlinEM hybrid pixel detector (Quantum Detectors Ltd., UK). The images and ED patterns were analyzed using the Fiji program.[15]

Cryo-transmission electron microscopy (cryo-TEM) of $CA_8C$

Perforated carbon film-covered microscopical 200 mesh grids (R1/4 batch of Quantifoil, MicroTools GmbH, Jena, Germany) were cleaned with chloroform and hydrophilised by 60 s glow discharging at 8 W in a BAL-TEC MED 020 device (Leica Microsystems, Wetzlar, Germany) before 4 µl aliquots of the hydrogel solution were applied to the grids. The samples were vitrified by automatic blotting and plunge freezing with a FEI Vitrobot Mark IV (Thermo Fisher Scientific Inc., Waltham, Massachusetts, USA) using liquid ethane as cryogen. The vitrified specimens were transferred to the autoloader of a FEI TALOS ARCTICA electron microscope (Thermo Fisher Scientific Inc., Waltham, Massachusetts, USA). This microscope is equipped with a high-brightness field-emission gun (XFEG) operated at an acceleration voltage of 200 kV. Micrographs were acquired on a FEI Falcon 3 direct electron detector (Thermo Fisher Scientific Inc., Waltham, Massachusetts, USA).

### 2.1.4 AFM imaging

Atomic force microscopy was performed with a JPK NanoWizard 4 AFM in tapping mode (AC mode) using Arrow NCR tip (42 N/m, 285 kHz, Nano World). 5 µL of suspension (final conc 0.1% (w/w)) were deposited on freshly cleaved mica, dried at room temperature, and analyzed the same day. AFM images were collected with 1024 x 1024 or 512 x 512 pixels/frame and analyzed with the JPK Data Processing software.

### 2.1.5 Rheology

Rheometric experiments were performed using a shear rheometer (MCR302, Anton Paar, Ostfildern-Scharnhausen, Germany). The measurements were performed with cone-plate geometry, using a diameter of 12 mm (CP-12, 0.025 mm gap, 1° cone angle; Anton Paar, Ostfildern-Scharnhausen, Germany). Each measurement was performed on a 20 μL sample at 25 °C. Water was filled in a trough around the sample table and a temperature-controlled hood was used. To determine the linear viscoelastic (LVE) region of the hydrogels, amplitude sweeps were performed. Strain amplitudes were varied from 1% to 100% while applying a constant angular frequency of 1 Hz. Frequency sweeps were subsequently conducted to study the frequency-dependent mechanical behavior of the network. The angular frequency was varied from 0.01 to 10 Hz while

applying a constant strain amplitude of 1% (value in the LVE region). To investigate the self-healing behavior of the hydrogels, a sequential step strain sweep test was carried out, where low and high amplitudes of oscillatory shear strains were alternated. $CA_8C$: After loading a sample, a constant strain amplitude of 1% with a constant angular frequency of 1 Hz was applied. Subsequently, a high strain amplitude of 100% was applied for 130 s, using the same angular frequency of 1 Hz. Recovery of G' was subsequently followed by applying oscillations with an amplitude of 1 % for 5 min.

## 2.2 XRD of monosaccharide functionalized oligomers

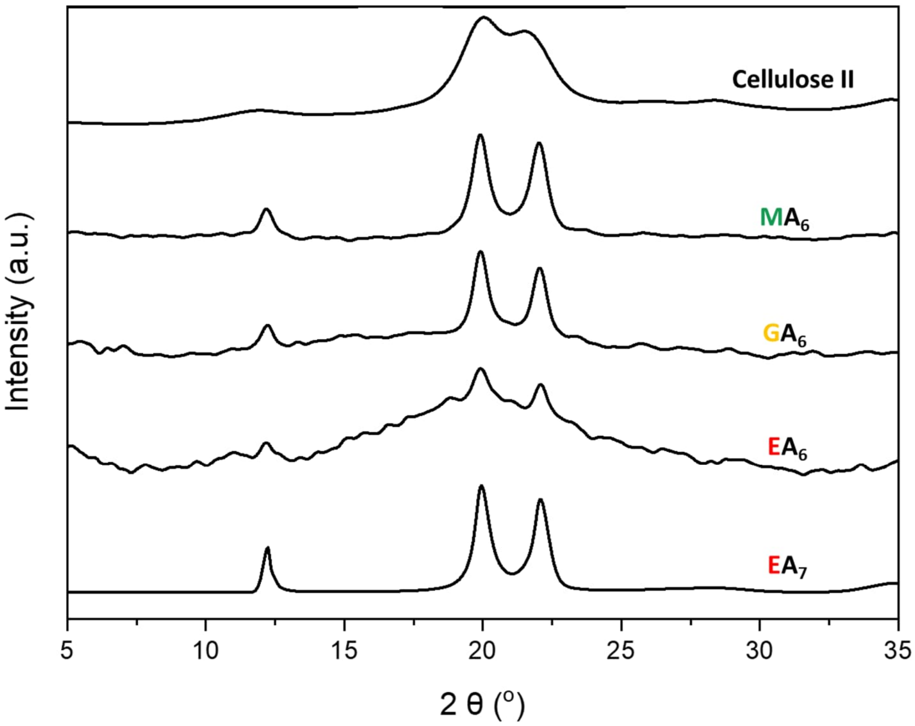


Figure S4 XRD analysis of oligomers $A_6$, $GA_6$, $MA_6$, $EA_6$ and $EA_7$.

## 2.3 TEM images of monosaccharide functionalized oligomers before and after annealing

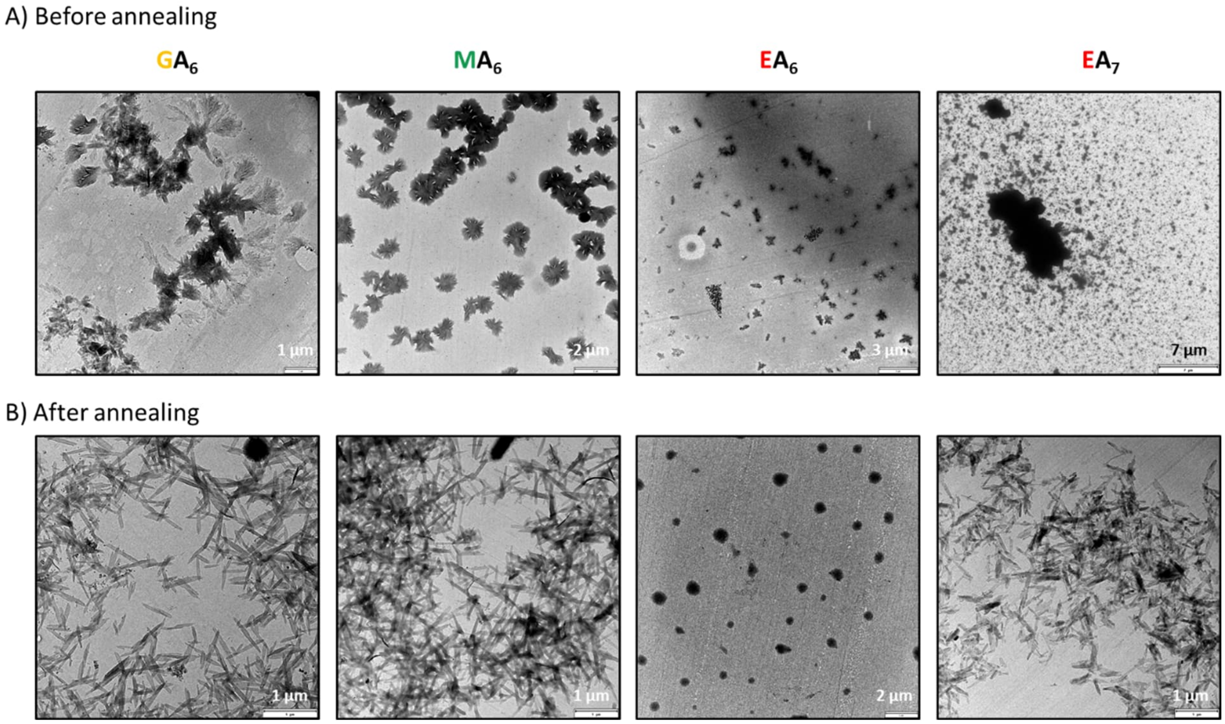


Figure S5 TEM imaging of $GA_6$, $MA_6$, $EA_6$ and $EA_7$ samples A) before and B) after annealing of 2.0 % (w/w) suspensions.

## 2.4 AFM imaging of monosaccharide functionalized oligomers after annealing

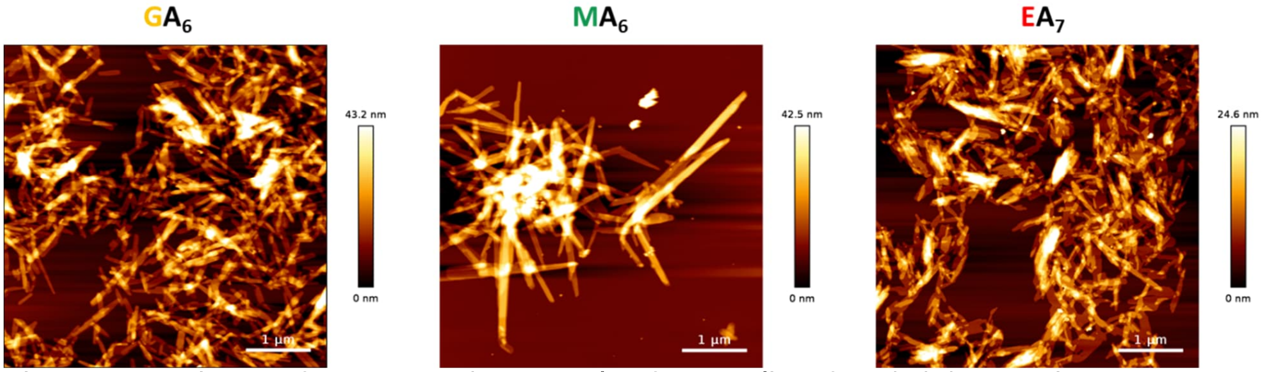


Figure S6 AFM images of $GA_6$, $MA_6$ and $EA_7$ samples after annealing of 2% (w/w) suspensions.

2.5 XRD of oligomers modified with C unit

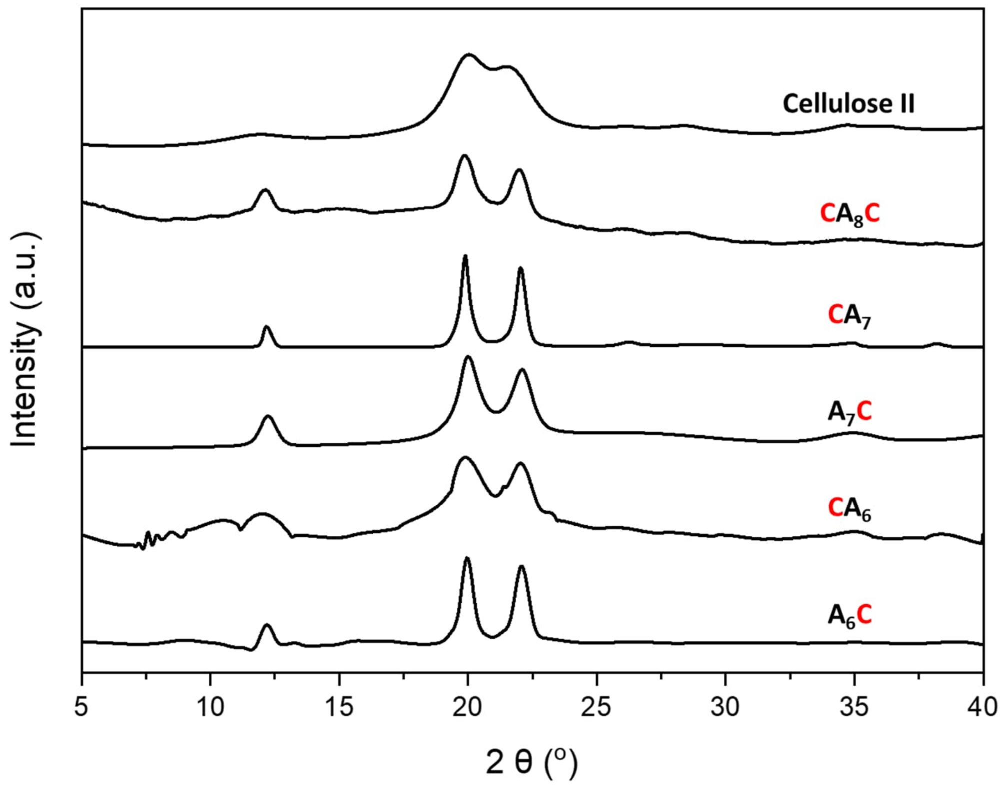


Figure S7 XRD analysis of oligomers $CA_8C$, $CA_7$, $A_7C$, $CA_6$ and $A_6C$.

## 2.6 TEM of oligomers modified with C unit before and after annealing

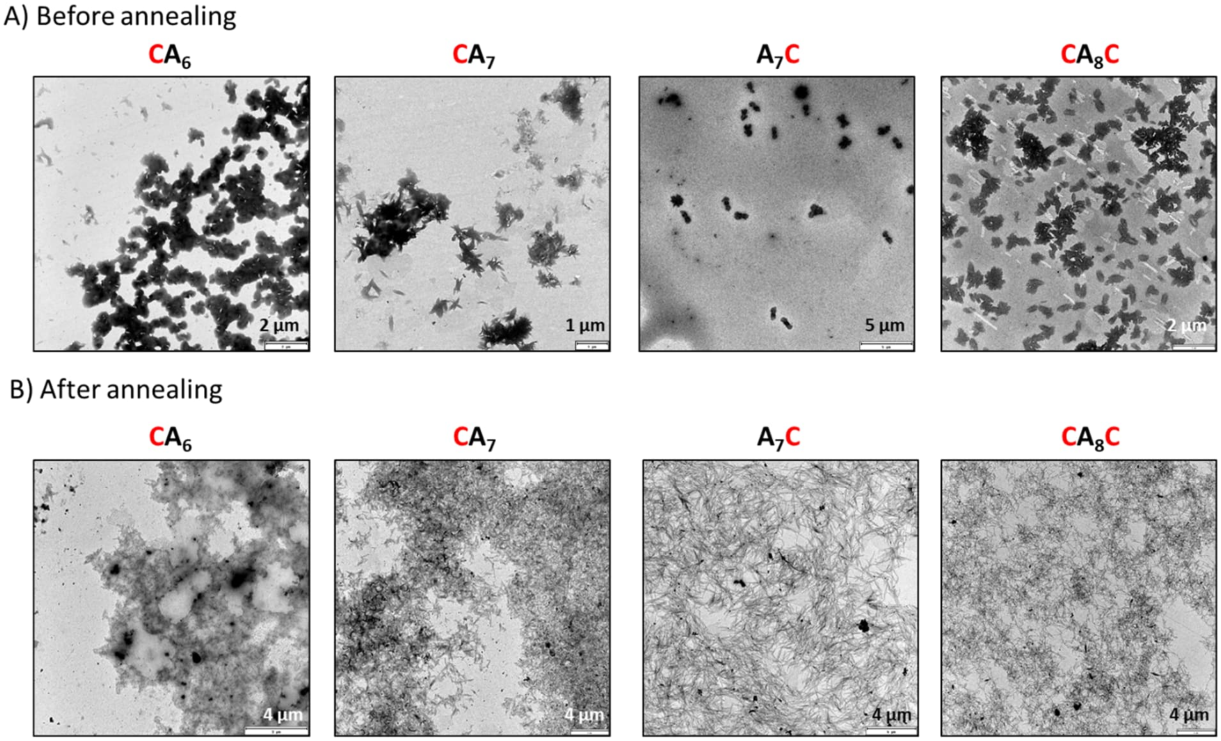


Figure S8 TEM imaging of $CA_8C$, $CA_7$, $A_7C$ and $CA_6$ samples A) before and B) after annealing of 2.0 % (w/w) suspensions.

## 2.7 AFM imaging of oligomers modified with C unit after annealing

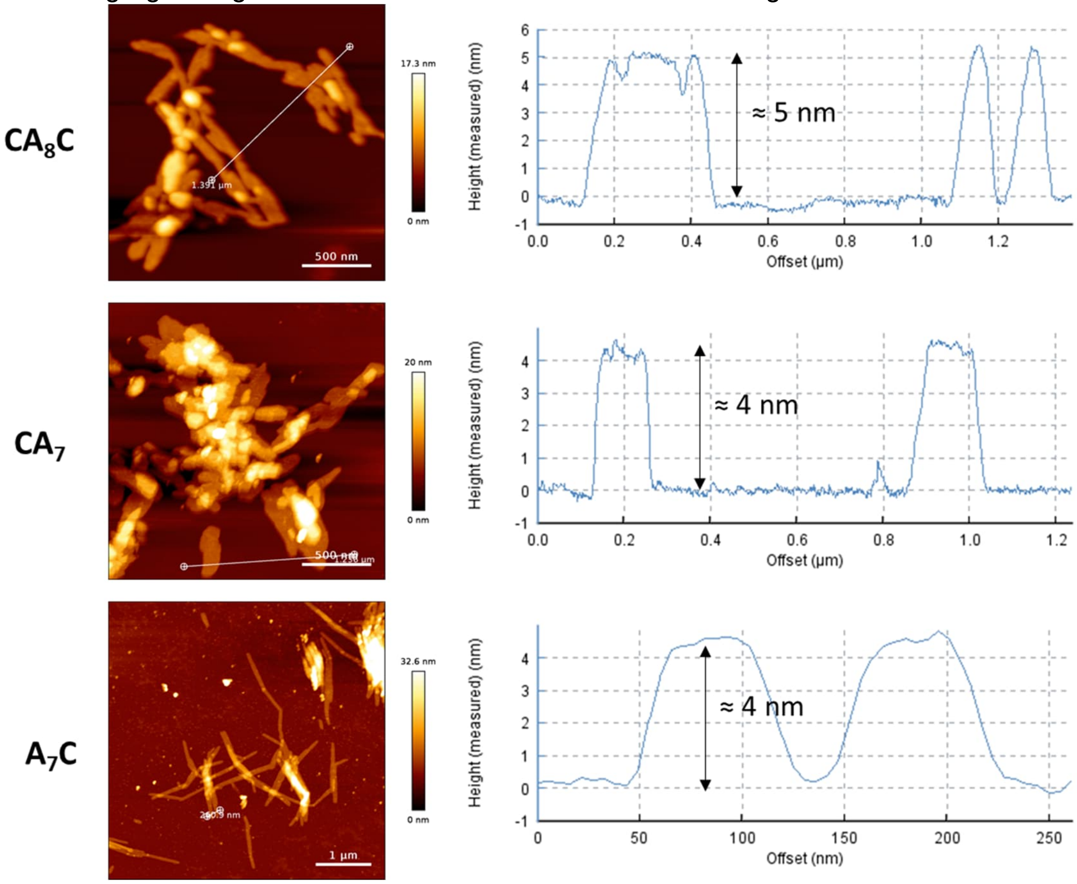


Figure S9 AFM imaging of $CA_8C$, $CA_7$, $A_7C$ samples after annealing 2% (w/w) suspensions.

## 2.8 Frequency sweeps of methylated cellulose oligomers

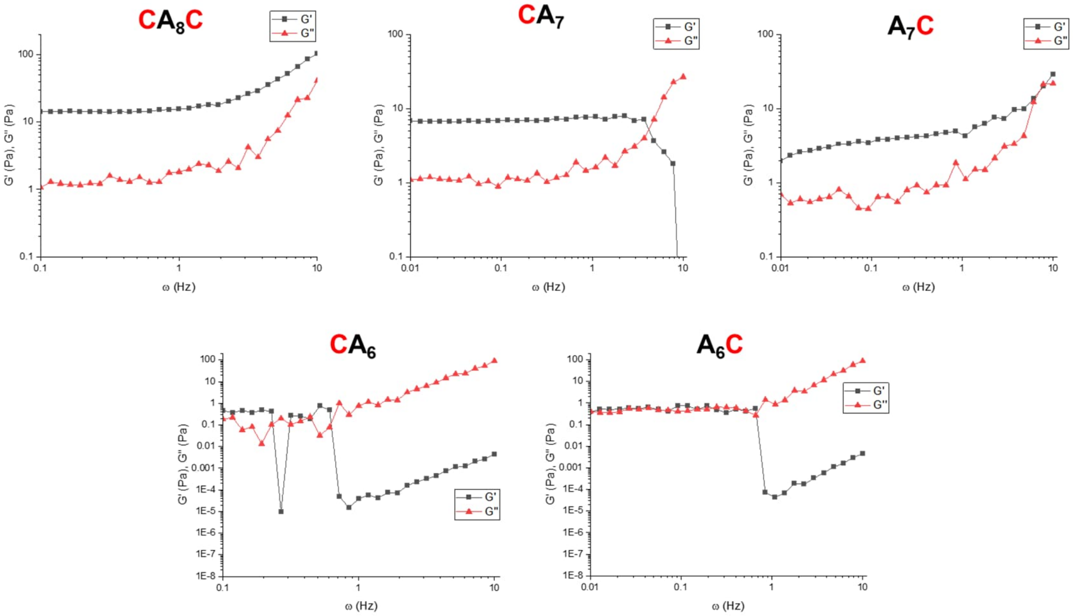


Figure S10 Frequency sweeps of 2.0 % (w/w) hydrogels from oligomers $CA_8C$, $CA_7$, $A_7C$ and $A_6C$.

## 2.9 Recovery test of CA$_8$C hydrogel

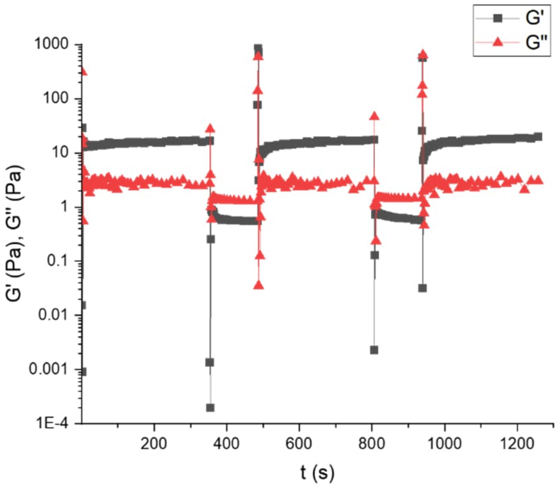


Figure S11 Recovery test of CA$_8$C 2.0 % (w/w) hydrogel.

## 2.10 Frequency sweeps of CA$_8$C hydrogels at different concentrations at different temperatures

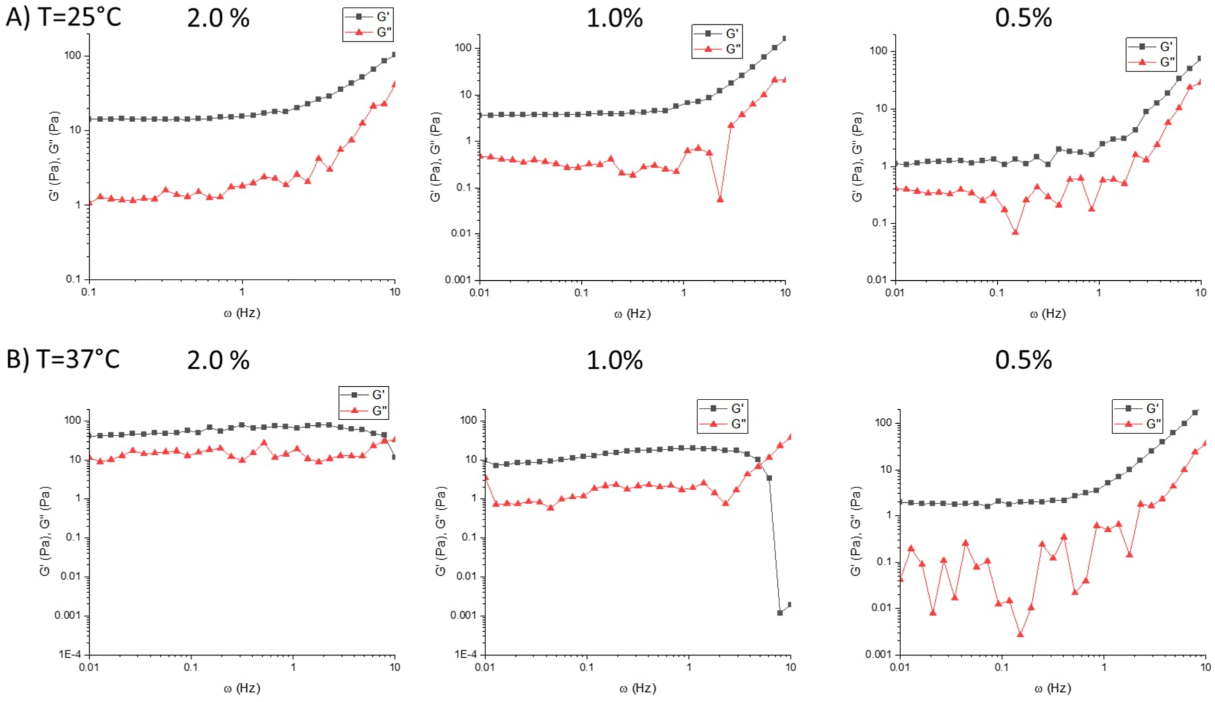


Figure S12 Frequency sweeps of CA$_8$C hydrogels at 2.0 %, 1.0 % and 0.5 % (w/w) concentration at A) 25°C and B) 37°C.

## 2.11 Comparison of storage and loss moduli of $CA_8C$ hydrogels at different temperatures

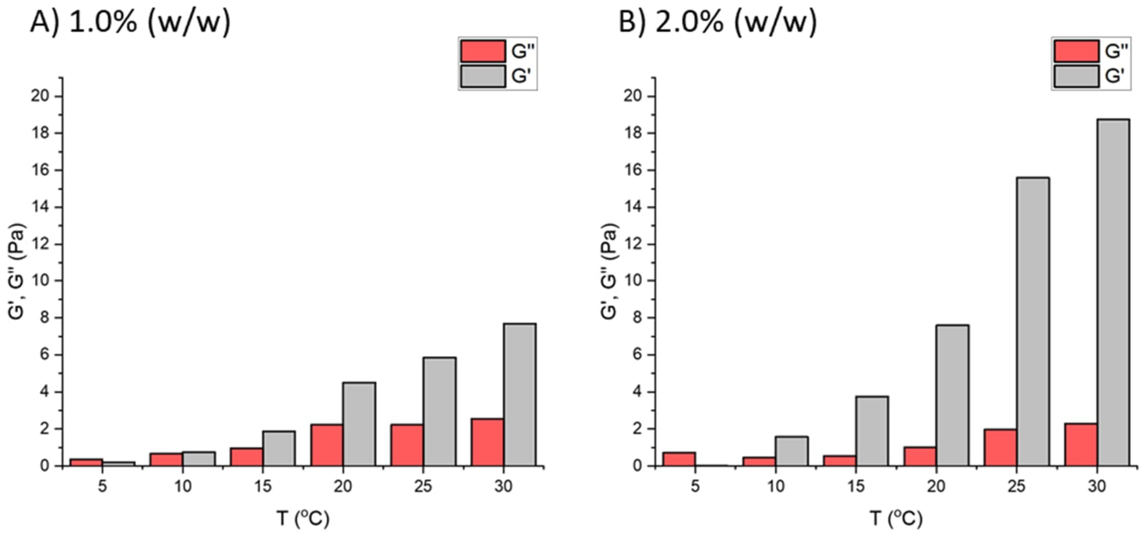


Figure S13 Comparison of storage and loss moduli of $CA_8C$ at A) 1.0 % and B) 2.0 % (w/w) hydrogels over a range of temperatures from 5°C to 30°C. The samples show liquid-like behavior at 5°C and transform into gel-like samples at 10°C. Storage (G') modulus significantly increases with an increase in temperature.

## 2.12 Rheology profiles of $CA_8C$ 1.0 % (w/w) hydrogel at different temperatures

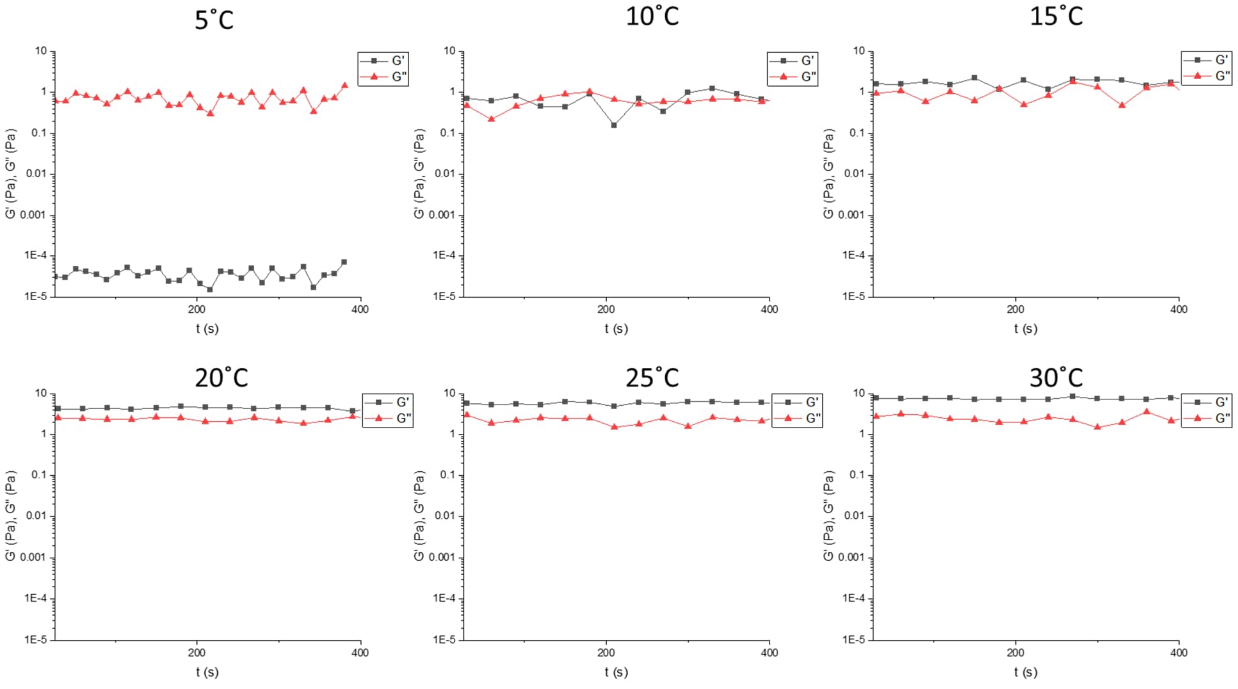


Figure S14 Rheology profiles of $CA_8C$ 1.0 % (w/w) hydrogel at different temperatures. (Shear strain 1%, frequency 0.5 Hz).

2.13 Rheology profiles of $CA_8C$ 2.0% (w/w) hydrogel at different temperatures

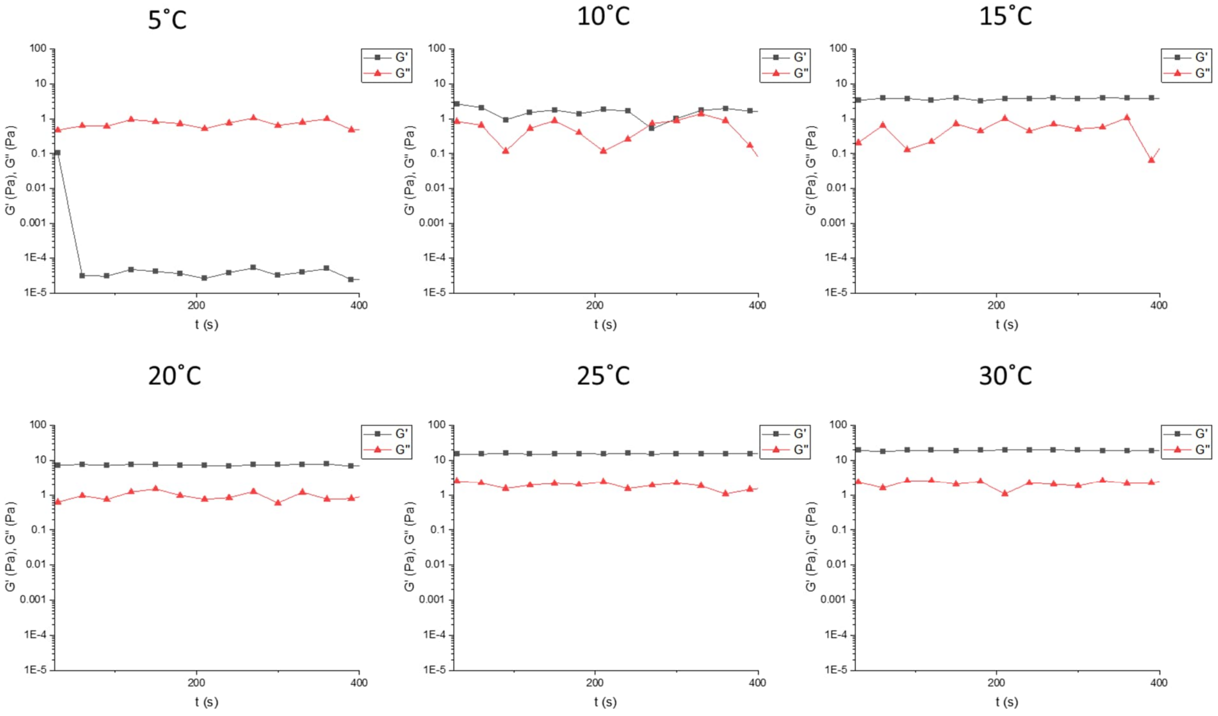


Figure S15 Rheology profiles of $CA_8C$ 2.0 % (w/w) hydrogel at different temperatures. (Shear strain 1%, frequency 1.0 Hz).

2.14 TEM of $CA_8C$ hydrogel at different concentrations.

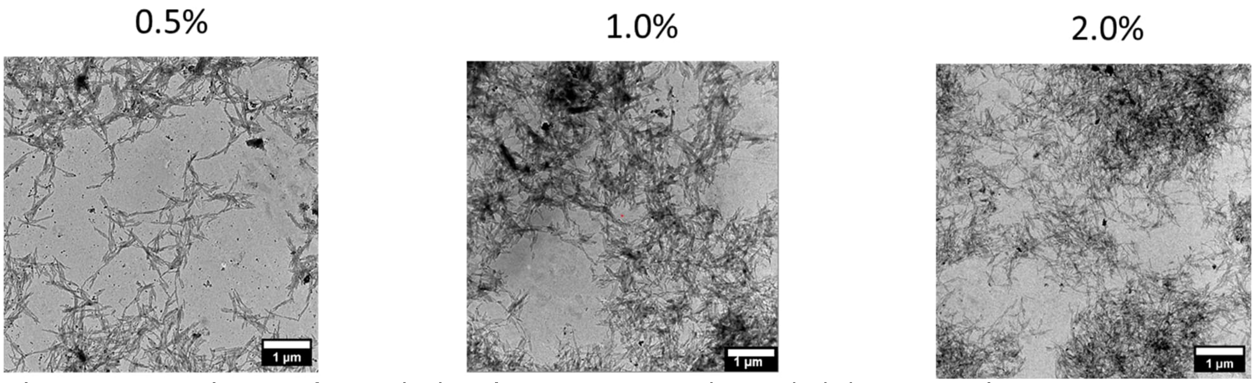


Figure S16 TEM images of $CA_8C$ hydrogel at 0.5%, 1.0% and 2.0% (w/w) concentration.

2.15 Cryo-TEM of $CA_8C$ hydrogel

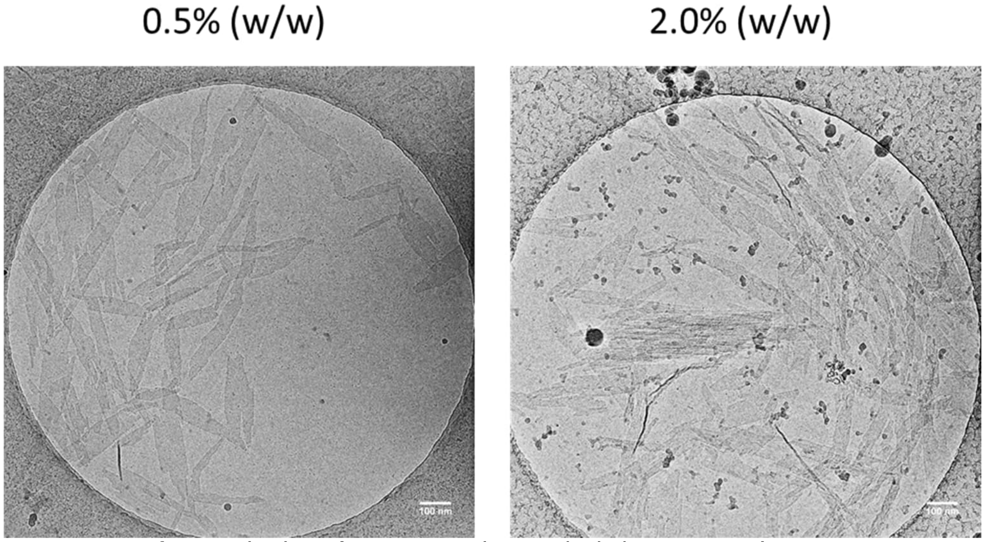


Figure S17 Cryo-TEM of $CA_8C$ hydrogel at 0.5% and 2.0% (w/w) concentration.

2.15a Cryo-ET of $CA_8C$ hydrogel

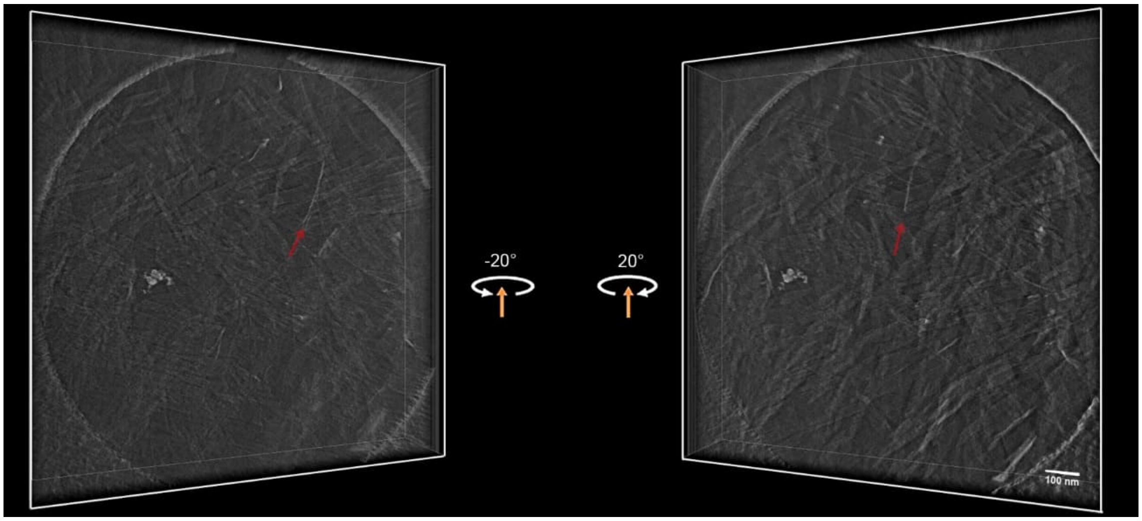


Figure S17a Cryo-electron tomogram of $CA_8C$ hydrogel at 2.0% (w/w) concentration, “voltex” presentation (note the inverted contrast) of the reconstructed 3D volume calculated from a tilt series (±64°, 2° increment) of the amorphous ice-embedded hydrogel, the corresponding rotation by ±20° around the y-axis provides a direct view on the edges of the nanocrystals (red arrows). Their measurement yielded a thickness of just under 6 nm.

2.16 XRD of $XA_8C$ oligomers

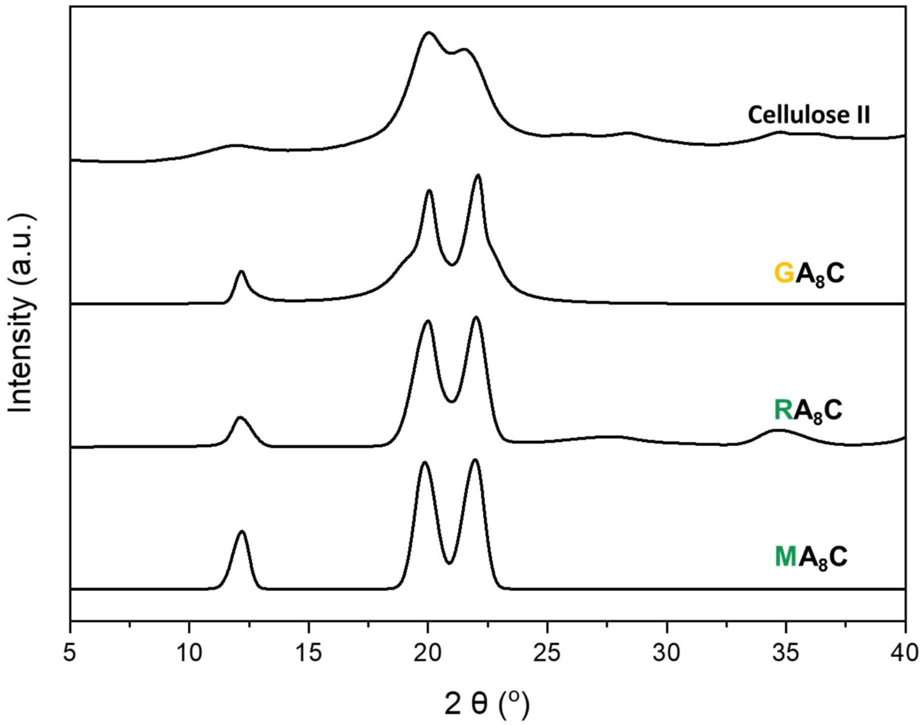


Figure S18 XRD analysis of oligomers $GA_8C$, $RA_8C$ and $MA_8C$.

## 2.17 AFM imaging of $XA_8C$ oligomers

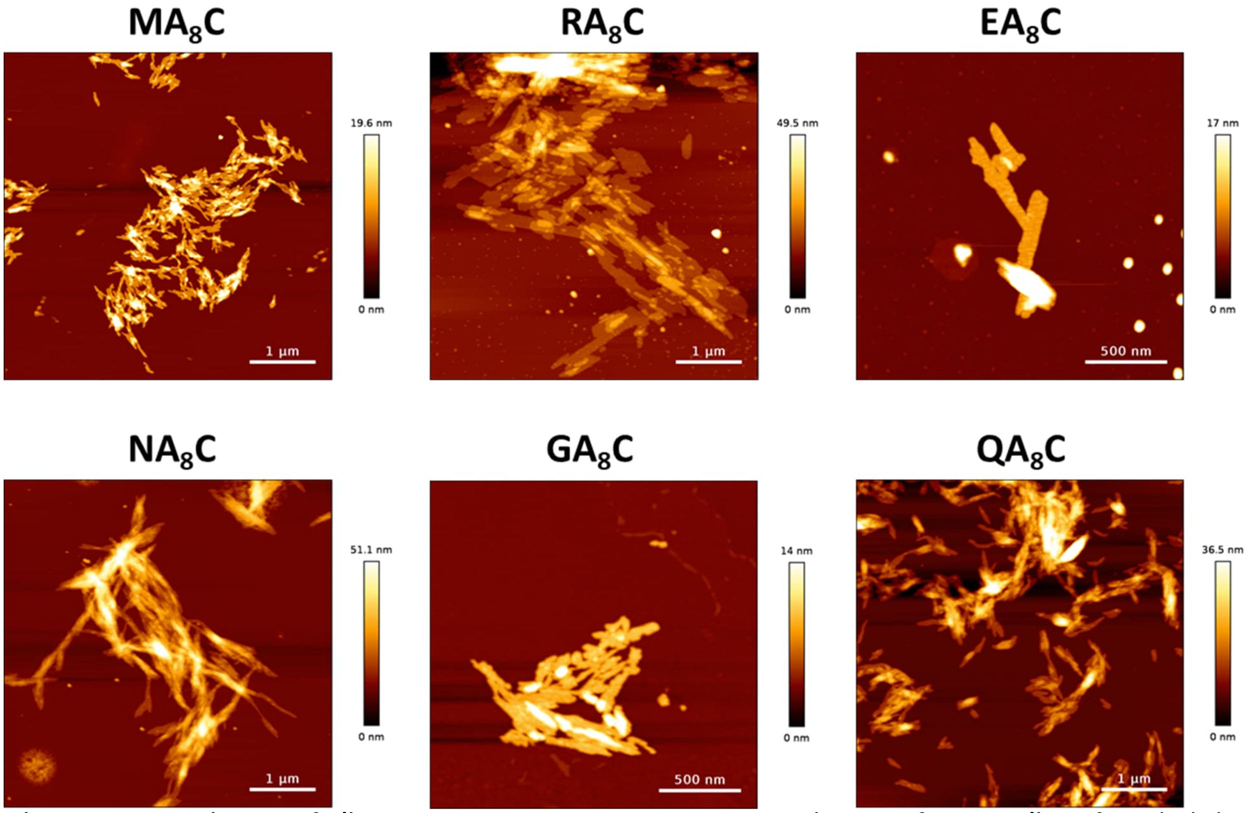


Figure S19 AFM images of oligomers $MA_8C$, $RA_8C$, $EA_8C$, $NA_8C$ and $GA_8C$ after annealing of 2% (w/w) suspensions.

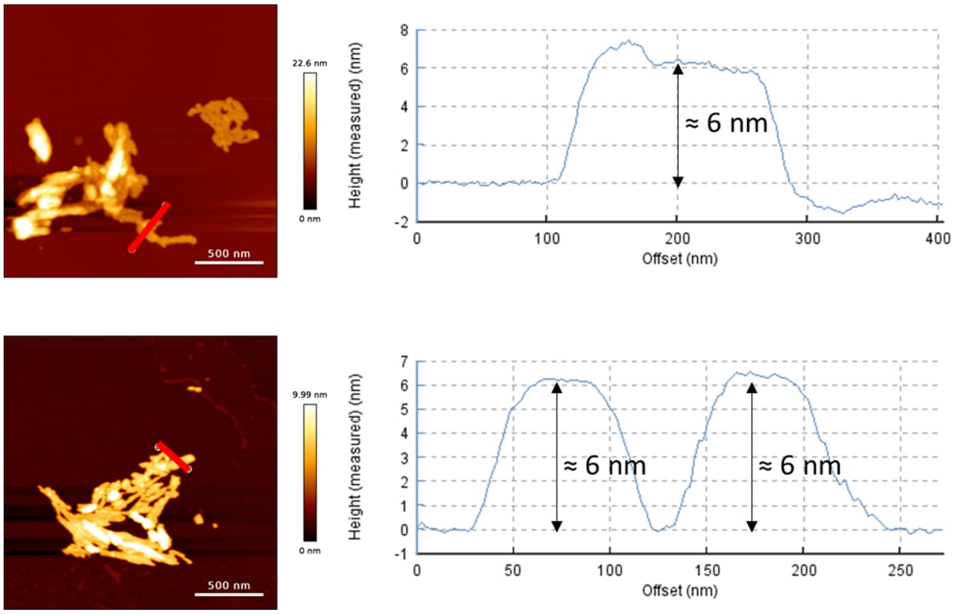


Figure S20 AFM imaging of oligomer $GA_8C$ after annealing of 2% (w/w) suspensions.

## 2.18 Frequency sweep experiments of functionalized hydrogels

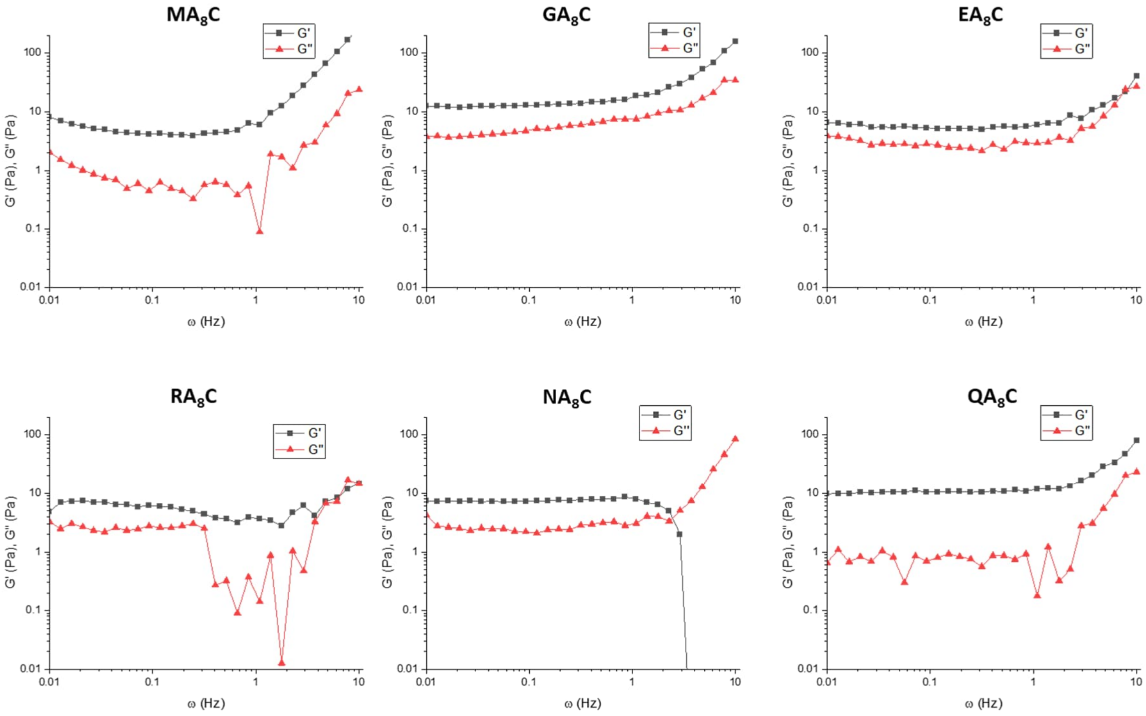


Figure S21 Frequency sweeps of 2% (w/w) hydrogels of oligomers MA8C, GA$_8$C, EA$_8$C, RA$_8$C, NA$_8$C and QA$_8$C at 25°C.

# 3 Biological assays

### 3.1.1 *C. albicans* strains and media

Strains were maintained on YPD agar (2% Bacto peptone, 2% glucose, 1% yeast extract, 2% agar) and grown at 30°C. Single colonies were inoculated into YPD broth and grown with shaking overnight at 30°C prior to each experiment. Experiments were performed with YPD medium with 10% fetal bovine serum (FBS). The *C. albicans* reference strain used in this study was SC5314.

### 3.1.2 Filamentation assay

Hyphal growth of *C. albicans* was induced by diluting cells to $OD_{600}$=0.05 into pre-warmed hyphae-inducing medium as indicated and incubating at 37°C in a glass-bottom, 384-well plate. Cells were grown in a mixture of hyphae-inducing medium (YPD+10% FBS) and hydrogels (2% (w/w)) for several hours, as described in the figure legends. Images were acquired with a confocal laser scanning microscope (LSM 800; Zeiss) equipped with a x63/1.4 NA oil-immersion. Images were analyzed using Zeiss ZEN 3.1.

### 3.1.3 Viability assay

Cell viability of *C. albicans* was determined from counting the colony forming units (CFU/mL) grown on YPD agar plates. Diluted *C. albicans* cells ($OD_{600}$=0.05) were inoculated into a mixture of hyphae-inducing medium (YPD+10% FBS) and hydrogels (2% (w/w)) and grown for 6 h or 24 h after which they were diluted and spotted on agar plates. The colonies were left growing at 30°C and counted after 24h.

## 3.2 Filamentation assay (1-3 h)

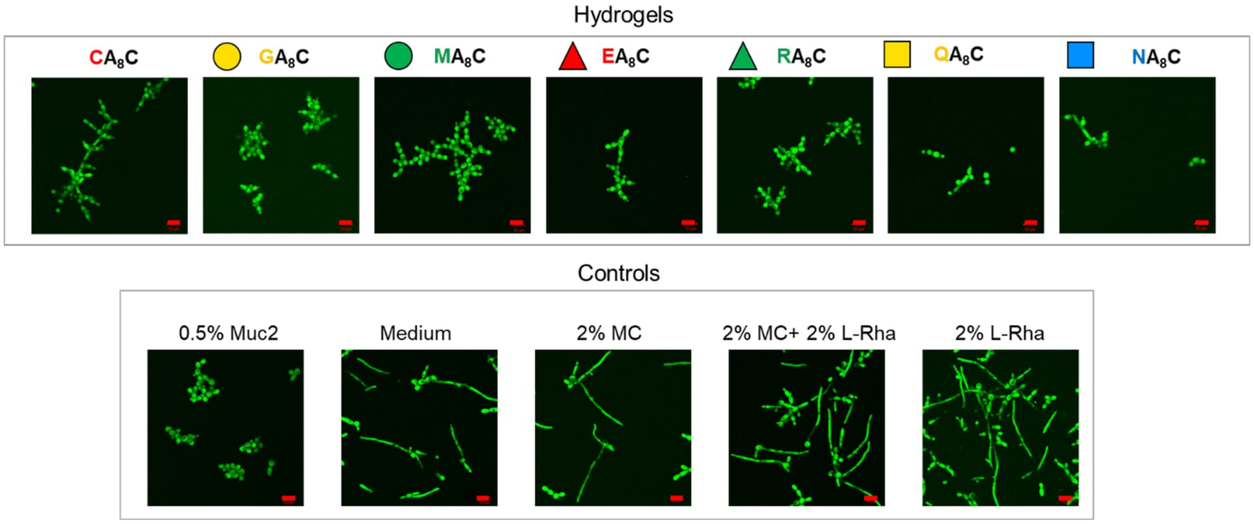


Figure S22 *C. albicans* filamentation assay in the presence of 2% (w/w) hydrogels ($CA_8C$, $GA_8C$, $MA_8C$, $EA_8C$, $RA_8C$, $QA_8C$ and $NA_8C$) and controls (0.5% (w/w) Muc2, medium, 2% (w/w) methylcellulose, 2% (w/w) methylcellulose + 2% (w/w) L-rhamnose, 2% (w/w) L-rhamnose) after 1-3 h. Scale bar 10 µm.

## 3.3 Filamentation assay (6-8 h)

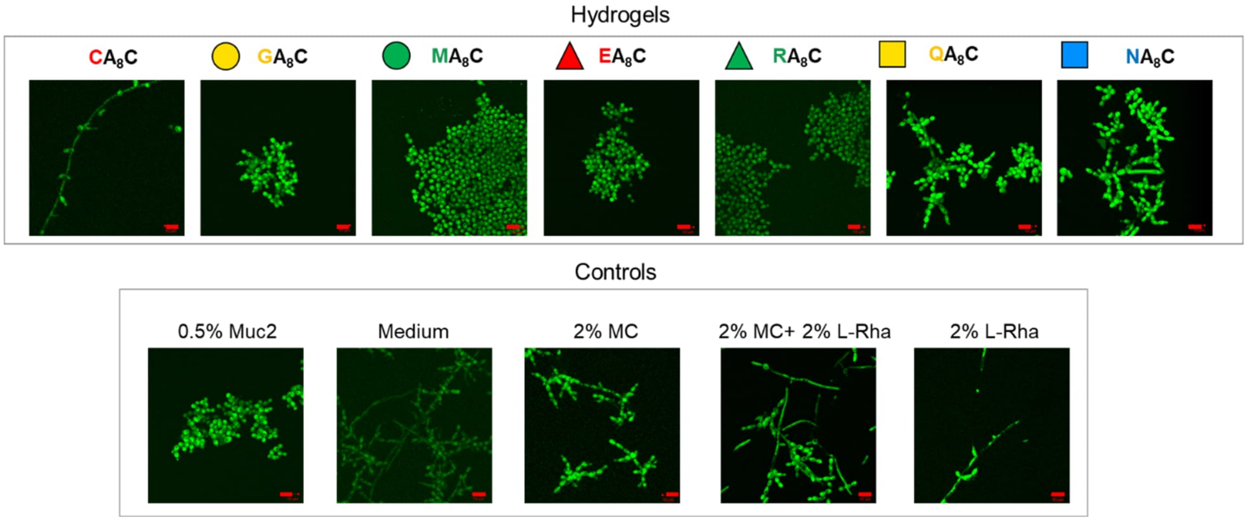


Figure S23 *C. albicans* filamentation assay in the presence of 2% (w/w) hydrogels ($CA_8C$, $GA_8C$, $MA_8C$, $EA_8C$, $RA_8C$, $QA_8C$ and $NA_8C$) and controls (0.5% (w/w) Muc2, medium, 2% (w/w) methylcellulose, 2% (w/w) methylcellulose + 2% (w/w) L-rhamnose, 2% (w/w) L-rhamnose) after 6-8 h. Scale bar 10 µm.

## 3.4 Filamentation assay 24 h

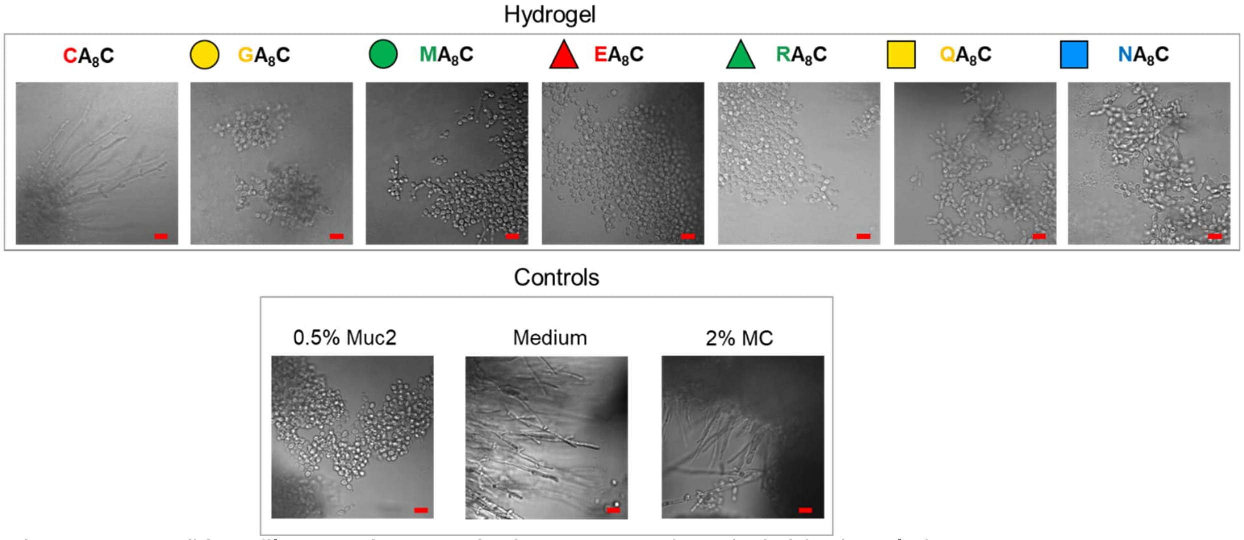


Figure S24 *C. albicans* filamentation assay in the presence of 2% (w/w) hydrogels ($CA_8C$, $GA_8C$, $MA_8C$, $EA_8C$, $RA_8C$, $QA_8C$ and $NA_8C$) and controls (0.5% (w/w) Muc2, medium, 2% (w/w) methylcellulose) after 24 h. Scale bar 10 µm.

### 3.5 Filamentation assay (48 h)

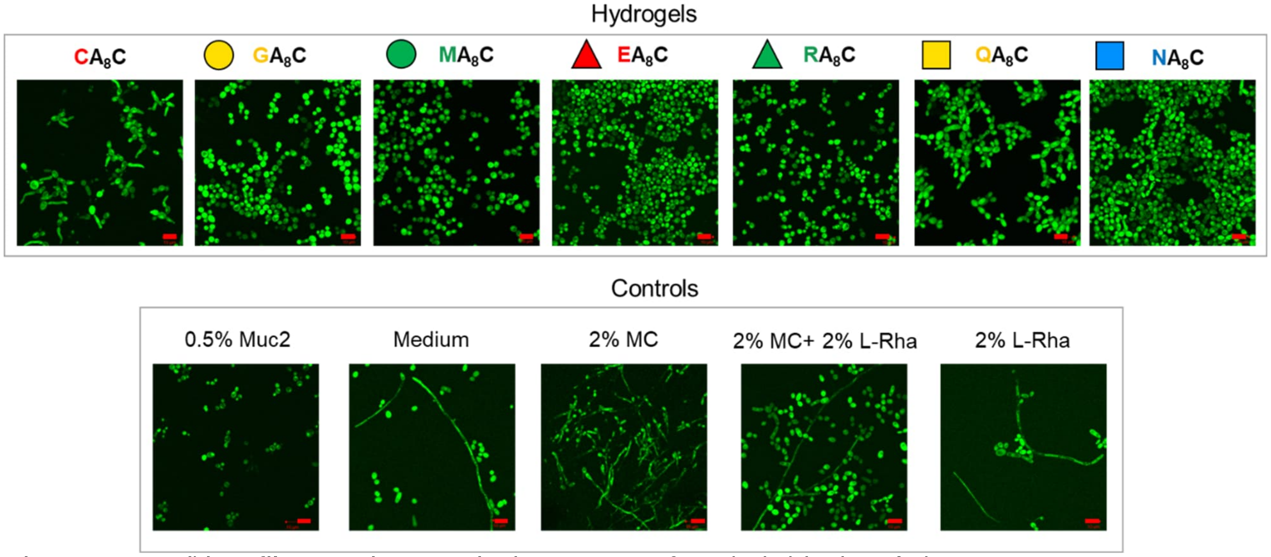


Figure S25 *C. albicans* filamentation assay in the presence of 2% (w/w) hydrogels ($CA_8C$, $GA_8C$, $MA_8C$, $EA_8C$, $RA_8C$, $QA_8C$ and $NA_8C$) and controls (0.5% (w/w) Muc2, medium, 2% (w/w) methylcellulose, 2% (w/w) methylcellulose + 2% (w/w) L-rhamnose, 2% (w/w) L-rhamnose) after 48 h. Scale bar 10 µm.

### 3.6 Viability assay

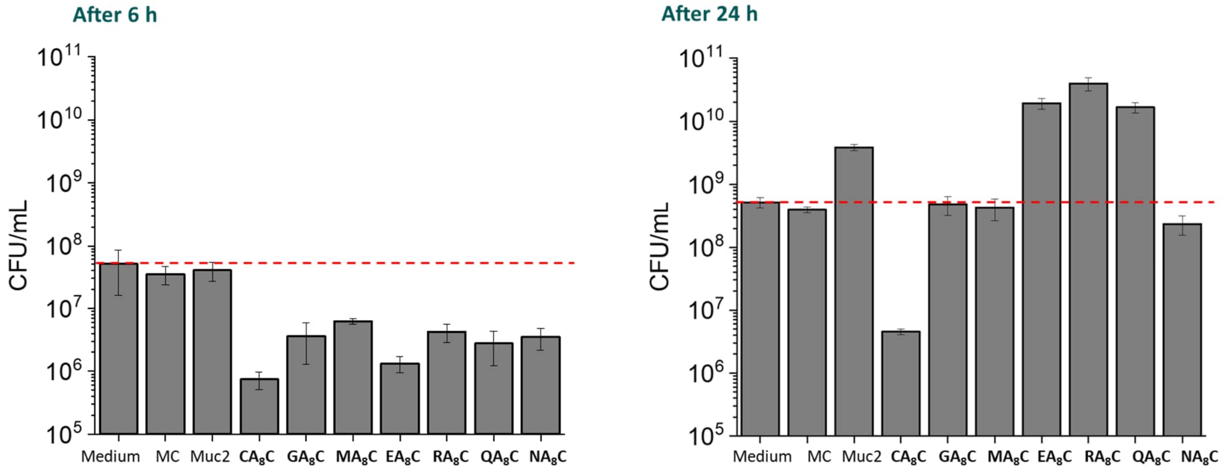


Figure S26 *C. albicans* viability assay after 6 h and 24 h (n=3).

# 4 References


[1] S. Eller, M. Collot, J. Yin, H. S. Hahm, P. H. Seeberger, *Angew. Chem., Int. Ed.* **2013**, *52*, 5858-5861.
[2] Y. Yu, S. Gim, D. Kim, Z. A. Arnon, E. Gazit, P. H. Seeberger, M. Delbianco, *J. Am. Chem. Soc.* **2019**, *141*, 4833-4838.
[3] P. Dallabernardina, F. Schuhmacher, P. H. Seeberger, F. Pfrengle, *Org. Biomol. Chem.* **2016**, *14*, 309-313.
[4] T. Tyrikos-Ergas, V. Bordoni, G. Fittolani, M. A. Chaube, A. Grafmüller, P. H. Seeberger, M. Delbianco, *Chem. – Eur. J.* **2021**, *27*, 2321-2325.
[5] G. Fittolani, T. Tyrikos-Ergas, A. Poveda, Y. Yu, N. Yadav, P. H. Seeberger, J. Jiménez-Barbero, M. Delbianco, *Nature Chemistry* **2023**, *15*, 1461-1469.
[6] K. Le Mai Hoang, A. Pardo-Vargas, Y. Zhu, Y. Yu, M. Loria, M. Delbianco, P. H. Seeberger, *J. Am. Chem. Soc.* **2019**, *141*, 9079-9086.
[7] J. Danglad-Flores, E. T. Sletten, E. E. Reuber, K. Bienert, H. Riegler, P. H. Seeberger, *Device* **2024**, *2*, 100499.
[8] M. Gude, J. Ryf, P. D. White, *Lett. Pept. Sci.* **2002**, *9*, 203-206.
[9] a) M. Guberman, M. Bräutigam, P. H. Seeberger, *Chem. Sci.* **2019**, *10*, 5634-5640; b) Y. Yu, T. Tyrikos-Ergas, Y. Zhu, G. Fittolani, V. Bordoni, A. Singhal, R. J. Fair, A. Grafmüller, P. H. Seeberger, M. Delbianco, *Angew. Chem., Int. Ed.* **2019**, *58*, 13127-13132.
[10] T. Tyrikos-Ergas, E. T. Sletten, J.-Y. Huang, P. H. Seeberger, M. Delbianco, *Chem. Sci.* **2022**, *13*, 2115-2120.
[11] M. Hurevich, J. Kandasamy, B. M. Ponnappa, M. Collot, D. Kopetzki, D. T. McQuade, P. H. Seeberger, *Org. Lett.* **2014**, *16*, 1794-1797.
[12] G. Fittolani, D. Vargová, P. H. Seeberger, Y. Ogawa, M. Delbianco, *J. Am. Chem. Soc.* **2022**, *144*, 12469-12475.
[13] D. Cheng, X. Chen, W. Zhang, P. Guo, W. Xue, J. Xia, S. Wu, J. Shi, D. Ma, X. Zuo, B. Jiang, S. Li, N. Xia, Y. Jiang, V. P. Conticello, T. Jiang, *Angew. Chem., Int. Ed.* **2023**, *62*, e202303684.
[14] D. N. Mastronarde, *Microsc. Microanal.* **2003**, *9*, 1182-1183.
[15] J. Schindelin, I. Arganda-Carreras, E. Frise, V. Kaynig, M. Longair, T. Pietzsch, S. Preibisch, C. Rueden, S. Saalfeld, B. Schmid, J.-Y. Tinevez, D. J. White, V. Hartenstein, K. Eliceiri, P. Tomancak, A. Cardona, *Nat. Methods* **2012**, *9*, 676-682.
